# Seeing through the eyes of the sabertooth *Thylacosmilus atrox* (Metatheria, Sparassodonta)

**DOI:** 10.1038/s42003-023-04624-5

**Published:** 2023-03-21

**Authors:** Charlène Gaillard, Ross D. E. MacPhee, Analía M. Forasiepi

**Affiliations:** 1 Instituto Argentino de Nivología, Glaciología y Ciencias Ambientales, CCT-CONICET Mendoza, Av. Ruiz Leal s/n, Parque General San Martín, CP5500 Mendoza, Argentina; 2 Department of Mammalogy, American Museum of Natural History, 200 Central Park West, 10024-5102 New York, NY USA

**Keywords:** Evolution, Palaeontology

## Abstract

The evolution of mammalian vision is difficult to study because the actual receptor organs—the eyes—are not preserved in the fossil record. Orbital orientation and size are the traditional proxies for inferring aspects of ocular function, such as stereoscopy. Adaptations for good stereopsis have evolved in living predaceous mammals, and it is reasonable to infer that fossil representatives would follow the same pattern. This applies to the sparassodonts, an extinct group of South American hypercarnivores related to marsupials, with one exception. In the sabertooth *Thylacosmilus atrox*, the bony orbits were notably divergent, like those of a cow or a horse, and thus radically differing from conditions in any other known mammalian predator. Orbital convergence alone, however, does not determine presence of stereopsis; frontation and verticality of the orbits also play a role. We show that the orbits of *Thylacosmilus* were frontated and verticalized in a way that favored some degree of stereopsis and compensated for limited convergence in orbital orientation. The forcing function behind these morphological tradeoffs was the extraordinary growth of its rootless canines, which affected skull shape in *Thylacosmilus* in numerous ways, including relative orbital displacement.

## Abstract (web summary)

The unique orientation of the orbits in the extinct hypercarnivorous sabertooth *Thylacosmilus atrox* shows that it was able to achieve binocular depth perception even though its cranium evolved for the growth and accommodation of its enlarged canines

## Introduction

Vision is part of a complex neurobehavioral sensory system that is critically important in most terrestrial vertebrates. Among mammals, primates and most carnivorans exhibit visual systems evolutionarily designed for stereoscopy, or the perception of depth^1^. In addition to retinal, cortical, and other soft-tissue mechanisms for integrating visual sensory fields (e.g.,^2^ and references therein), certain cranial modifications are thought to enhance the ability to collect visual imagery. Foremost among these is the presence of forward-facing (or convergent) bony orbits^1–4^. A high level of orbital convergence enables significant visual field overlap, which is fundamental for neurological processing of depth information^1–5^.

Studies reveal that stereoscopy enhances the effectiveness of focus-and-follow behaviors in active predators, but to the detriment of wide-area perception due to close approximation of the eyes^1,3,5–9^. By contrast, prey species usually exhibit laterally diverging orbits, which are more appropriate for panoramic rather than 3D vision (e.g.,^1,9,10^). Stereoscopy is not limited to placental mammals. Marsupials have highly convergent orbits, and visually-directed predation occurs in both placental and marsupial carnivores^1,5,11–13^. Sparassodonta is an extinct clade of carnivorous, nonmarsupial metatherians that lived in South America through most of the Cenozoic until their extinction in the mid-Pliocene (e.g.,^14,15^). Their orbital orientations follow the expected pattern, with one egregious exception: *Thylacosmilus atrox*, the sparassodont famously called the “sabertooth marsupial”, presents an unexpected configuration of the bony orbits that differs not only from conditions in all other investigated metatherians, but also stands as a dramatic departure from more conventional patterns of stereoscopy that are traditionally thought to apply to predaceous mammals.

**Fig. 1:**
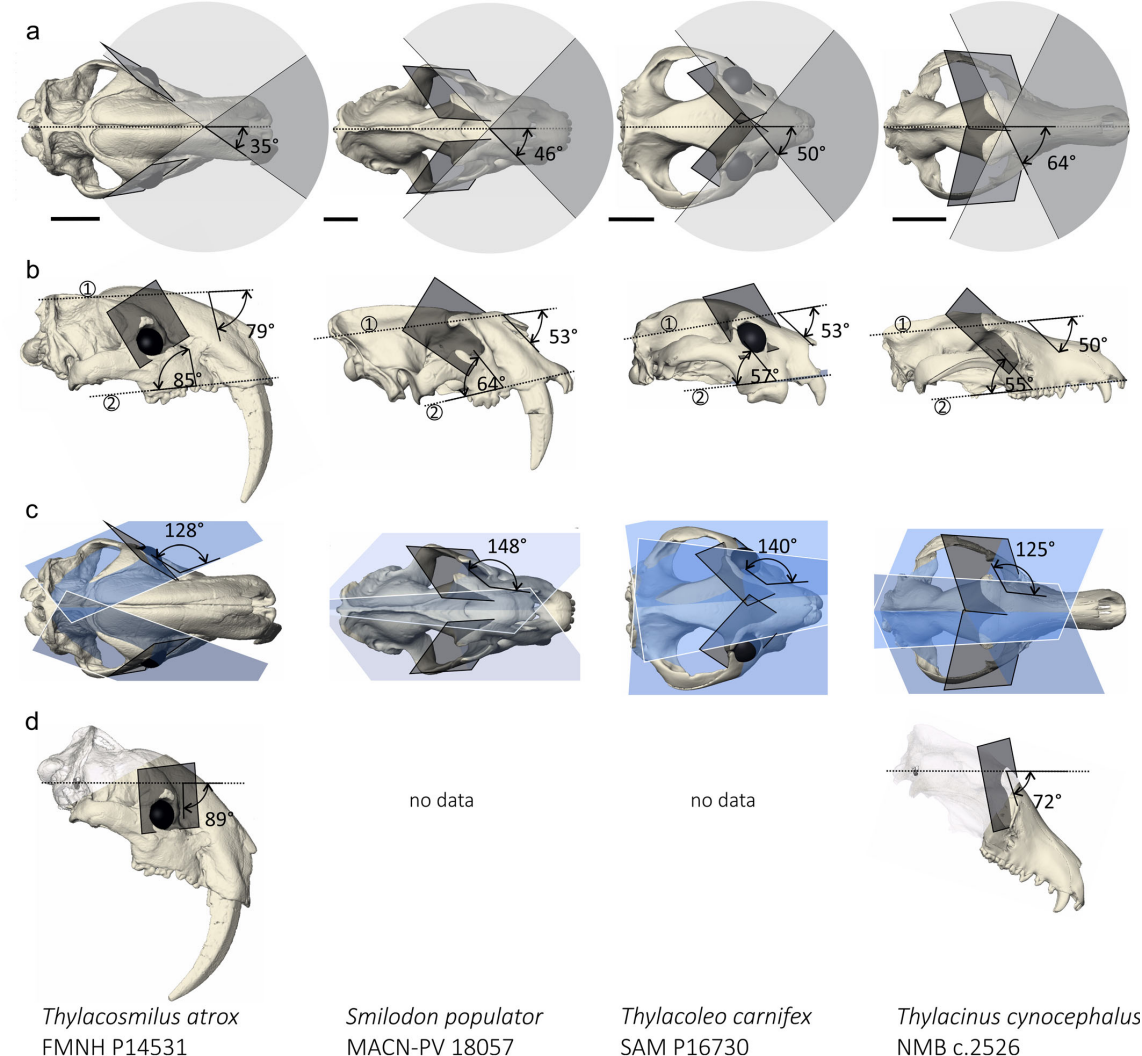
Orbital orientation following Heesy (11) and virtual eye reconstruction. **a** Convergence. **b** Orbital verticality (1) and frontation (2). **c** Orbitotemporal angle. **d** Orbitolabyrinth angle. The lateral view of *T*. *carnifex* is left lateral view mirrored. Scale bar is 5 cm.

The most conspicuous attributes of the skull of *Thylacosmilus* are its enlarged and hypsodont upper canines, but there are many other apomorphies unique to this taxon among sparassodonts and marsupials alike (Fig. 1). For example, the maxillae are massively overgrown, to such an extent that they almost completely cover the reduced nasals and overlap the top of the skull. The temporal area of the rostrocaudally foreshortened neurocranium is bounded by strong temporal, sagittal, nuchal, and supraglenoid crests, while the hyperobust occipital region displays large ventral protuberances for prevertebral muscles^16–19^. *Thylacosmilus* also developed a complete postorbital bar; although this structure is found in various placentals (e.g., primates, some carnivorans, horses, and artiodactyls^6,11,20^), with the exception of *Thylacoleo carnifex* it is not present in other metatherians^3,21^. Different combinations of these features are seen in other “sabertooths” (e.g., felids *Smilodon*, *Homotherium*; creodont *Machaeroides*; nimravid *Barbourofelis*^16,18,22–25^), but the sheer number of maxillofacial autapomorphies in *Thylacosmilus* suggests that they stem from a common cause—in this case, a developmental cascade, related to canine hypertrophy, that resulted in reorganization of its orbital region.

To test this possibility, we analyzed a suite of homologous orbital parameters in *Thylacosmilu*s and a range of other mammals. Our results are consistent with the hypothesis that, at some point in the evolution of the lineage of *Thylacosmilus*, the appearance of oversized, evergrowing canines affected cranial ontogeny in such a way that the orbital axes were progressively displaced laterally relative to conditions in other sparassodonts. In the absence of any compensatory changes, this growth trajectory would have resulted in eyes positioned for effective panoramic vision like that of a horse or a cow, not a predator. Yet its dental apparatus and related cranial features indicate that it was a true hypercarnivore (^26^; see Supplementary Note 1) and therefore presumably reliant on being able to optically assess crucial variables like prey distance and motion. As we discuss in Results, this bizarre combination of adaptations challenges the concept that adaptations for carnivory (such as stereopsis) are highly constrained to one or a few pathways.

## Results

Specimens used for this study are listed under Material and Methods. In the text, the following acronyms are employed for brevity of reference: *Thylacosmilus* H, holotype (FMNH P-14531); *Thylacosmilus* M (MMP 1433-M); NT sparassodonts, non-*Thylacosmilus* sparassodonts; BH, Borhyaenidae; HC, Hathliacynidae. Orbital orientation angles and other measurements for all specimens are given in Supplementary Table S3 and S4. Common names are used for other taxa referenced in the text; their binomials are listed in Supplementary Data 1.

For mensurational purposes, the bony orbit can be conceptualized as a plane defined by three landmarks (Supplementary Fig. S1 and Table S2). Previous methods vary in their choice of landmarks^11,12,27,28^. Heesy’s method^11^ (Fig. 1; Supplementary Methods) was chosen for utilization in the main text because the resulting orbital plane relates to biological significance and measurements of angles are easily acquired. (Results from other methods are detailed in Supplementary Results). The comparative database available for use with this method is extensive; very small mammals (e.g., tree shrews, up to 150–200 g;^29^) were excluded as biologically uninformative given the estimated body size of *Thylacosmilus* (~100 kg;^30^).

Quantified variables are defined as follows (see Supplementary Table S2 and Supplementary Discussion on the methods). Convergence represents a quantification of the amount of left/right orbital overlap. Frontation and verticality quantify the tilting of the orbits in relation to, respectively, the inion-nasion axis and the palatal plane^3,11^. Orbitotemporal angle is a measure of the orientation of the temporal fossae in relation to the orbits.

### Orbital convergence

Mean orbital convergence of *Thylacosmilus* H is 34.8° (Fig. 1a) and 30.7° for *Thylacosmilus* M. Values for NT sparassodonts range between 47.0 and 86.1° with mean value of 63.2° for HC and 56.0° for BH. *Thylacosmilus* exhibits one of the smallest values for orbital convergence recorded among metatherians, including sparassodonts as well as marsupials (41.0–65.2°; e.g., *Thylacoleo*: 49.8°), most extant carnivorans (34.2–71.2°), and sabertooth fossil cats (39.6–45.3°) (Supplementary Figs. S12 and S13). Compared to non-carnivorous placentals^11^, *Thylacosmilus* has orbital convergence values similar to some artiodactyls (e.g., red deer: 30.3°, Bohor reedbuck: 33.3°) (^3^; Supplementary Data 1).

### Orbital verticality

**Fig. 2:**
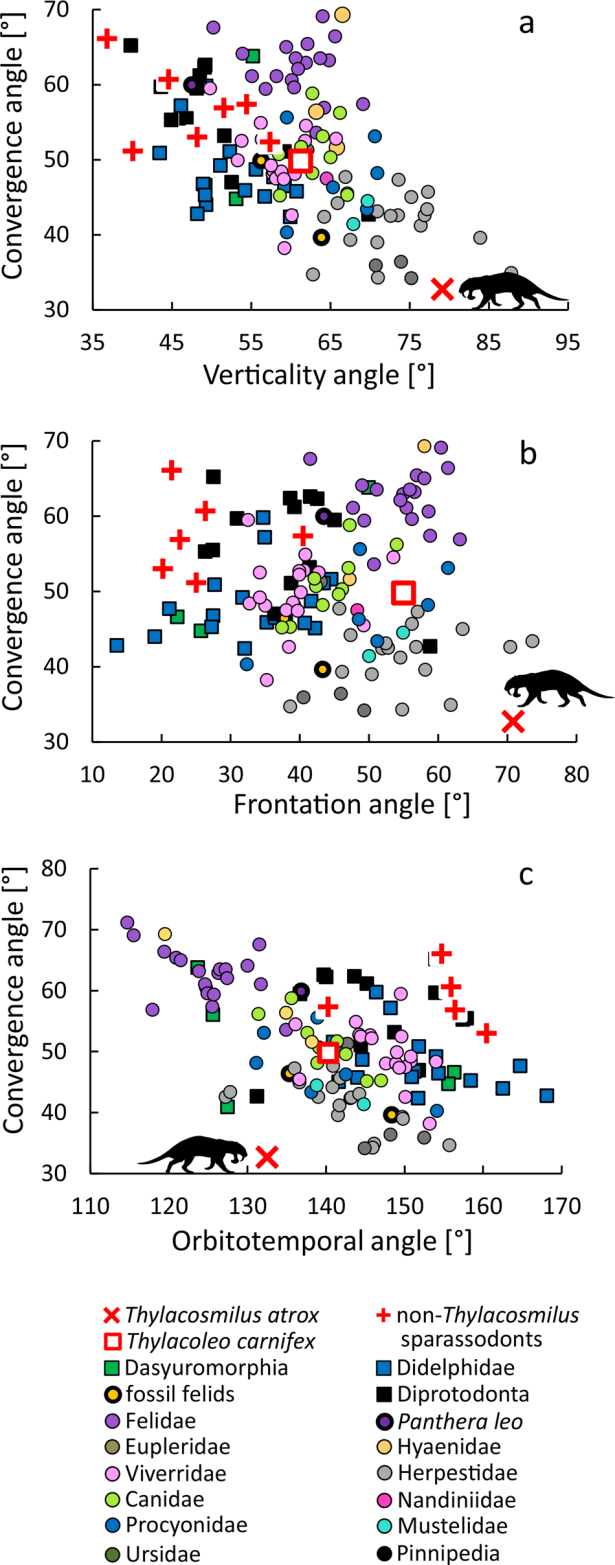
Comparative set, bivariate plots of selected angles following Heesy^11^. **a** Convergence and verticality angles. **b** Convergence and frontation angles. **c** Convergence and orbitotemporal angles.

*Thylacosmilus* H, 85.3° (Fig. 1b); *Thylacosmilus* M, 73.0°; NT sparassodonts range, 30.8–62.2°; HC, 42.3°; BH, 51.8°. Verticality values for *Thylacosmilus*, among the highest in our comparative set, are similar to those of equids, artiodactyls, hyracoids, folivorans, and herpestids^11^. When convergence is plotted against verticality (Fig. 2a and Supplementary Figs. S2 and S3), NT sparassodonts cluster with extant marsupials and *Thylacoleo* (61.2°), not far from sabertooth felids (*Smilodon populator:* 63.9°; *Homotherium serum*: 55.9°). By contrast, *Thylacosmilus* plots near bovids, far from other metatherians as well as carnivorans.

### Orbital frontation

Values for *Thylacosmilus* (H, 79.2°; M, 62.5°) are much higher than those for NT sparassodonts (16.4–43.2°) (Fig. 1b), as well as higher than the maximum values registered for marsupials (eg., *Sarcophilus*: 61.4°; *Thylacinus*: 55.2°; *Thylacoleo:* 54.9°) (Fig. 2b). Among placentals, only some artiodactyls, perissodactyls, and herpestids^11^ are similar, while sabertooth felids are lower (*Smilodon fatalis*: 37.8°; *S. populator*: 43.3°). When plotting convergence against frontation (Fig. 2b and Supplementary Figs. S4 and S5), *Thylacosmilus* plots closest to artiodactyls (red deer, oribi, Mongolian gazelle, and white-lipped peccary), at a considerable distance from NT sparassodonts and carnivorans. Notably, NT sparassodonts group together, near extant marsupials, with *Borhyaena* being an outlier; *Thylacoleo* by contrast lies considerably further away. *Smilodon gracilis* and *S*. *fatalis* do not cluster with felids but with herpestids, viverrids, euplerids, and some canids.

### Orbitotemporal angle

*Thylacosmilus* H, 128.3° (Fig. 1c); *Thylacosmilus* M, 141.0°; NT sparassodonts range, 139.6–169.7°; HC, 155.1°; BH, 150.3°. The orbitotemporal angle of *Thylacosmilus* is smaller than that of NT sparassodonts, with the exception of *Borhyaena* (140.3°). These values are among the lowest recorded for marsupials (123.7–168.1°; e.g., *Thylacoleo*: 140.3°; *Thylacinus*: 123.7°; *Sarcophilus harrisii*: 127.4°). *Smilodon populator* and *S*. *fatalis* have orbitotemporal angles (135.4° and 148.4°, respectively) close to the range covered by *Thylacosmilus*, *Borhyaena*, and *Thylacoleo* (Supplementary Figs. S18 and S19). When compared to non-carnivorous placentals, *Thylacosmilus* is once again most similar to extant artiodactyls, especially cervids, bovids, tragulids, and tayassuids (^3^, Supplementary Data 1). However, when orbital convergence is plotted against orbitotemporal angle (Fig. 2c; Supplementary Figs. S6–7 and S11), *Thylacosmilus* is isolated from all other taxa in this survey. NT sparassodonts group near marsupials, *Borhyaena* again being an exception. *Thylacoleo* is surrounded by canids, but not far from other marsupials. *Thylacinus* is situated proximate to felids, away from other marsupials, while sabertooth felids fall within the herpestid cluster.

### Orbitolabyrinth angle

*Thylacosmilus* exhibits an almost vertical (88.6°) orientation of the orbital plane relative to the lateral semicircular canal (Fig. 1d; Supplementary Fig. S34). This configuration is very similar to the one found in the placental sabertooth *Smilodon fatalis* (85.0°). In comparison, metatherians have lower values (*Thylacinus*, 71.7°; *Dasyurus hallucatus*, 59.7°; *Didelphis virginiana*, 74.7°), with the NT sparassodont *Sipalocyon gracilis* having the lowest orbitolabyrinth angle (36.0°).

## Discussion

The notably trenchant cheekteeth of sparassodonts mark them as hypercarnivores, a morphological category today occupied by specialized carnivorans such as cats and hyaenas^15,26^. As expected, NT sparassodonts analyzed for this study exhibit high orbital convergence, a characteristic feature of extant felids and hyaenids. Among extant marsupials, orbital orientation parameters in NT sparassodonts are similar to those found in non-caluromyine didelphids (high convergence and low verticality angle;^3,12^), although the former show slightly lower values for verticality (Fig. 2a). Differences detected between scansorial hathliacynids (*Cladosictis*, *Sipalocyon*) and the more terrestrial borhyaenids (*Borhyaena*, *Arctodictis*) could be explained by their contrasting locomotor styles (e.g.,^18,19,22,31–35^), as arboreality is thought to correlate with greater orbital convergence^10^ (but see ref. ^36^). Alternatively, differences among sparassodont families might reflect the effect of snout proportions on orbital orientation^12^. However, in this scenario borhyaenids would be expected to display higher convergence values than hathliacynids, which is not the case. The relationship between orbital orientation and snout proportions in metatherians seems to be more complex than previously thought based on the pattern observed in didelphids^12^ and in placentals^20^.

Unlike other sparassodonts, *Thylacosmilus* displays low convergence of visual fields (Fig. 1a). While this is not incompatible with a carnivorous diet (e.g., spotted linsang, mongooses), the only examples among extant carnivorans are taxa having small body sizes and possibly related physical constraints due to their relatively larger eyes (^1^; see^5^), which is not the case in *Thylacosmilus*^18,30,33–35^. Potentially, the estimated field of binocular vision in *Thylacosmilus* might have been 40–80°, but this is actually far beneath the range expected for an active predator (e.g., *Canis*, 78–116°; *Felis*, 120°; *Dasyurus*, 125°;^5^). A greater degree of binocularity might have been achieved by *Thylacosmilus* if its eyes were orientated in their sockets in such a way as to enhance convergence of their visual axes. This possibility has been hypothesized for the predaceous marsupials *Dasyurus* and *Sminthopsis* (orbital convergence < 42°, but with actual overlap of visual fields of 125–140°,^5^). However, true visual axes cannot be ascertained in fossils. Another way to compensate for low convergence is to increase eyeball diameter, which was relatively greater in *Thylacosmilus* than in other metatherians (SI Appendix, Table S3). Increased eyeball size has been correlated with greater visual acuity in primates^36^. In mammals, however, intraspecific differences in eyeball size plays a role in visual acuity^37^; such data are impossible to collect for fossil mammals lacking a postorbital bar.

**Fig. 3:**
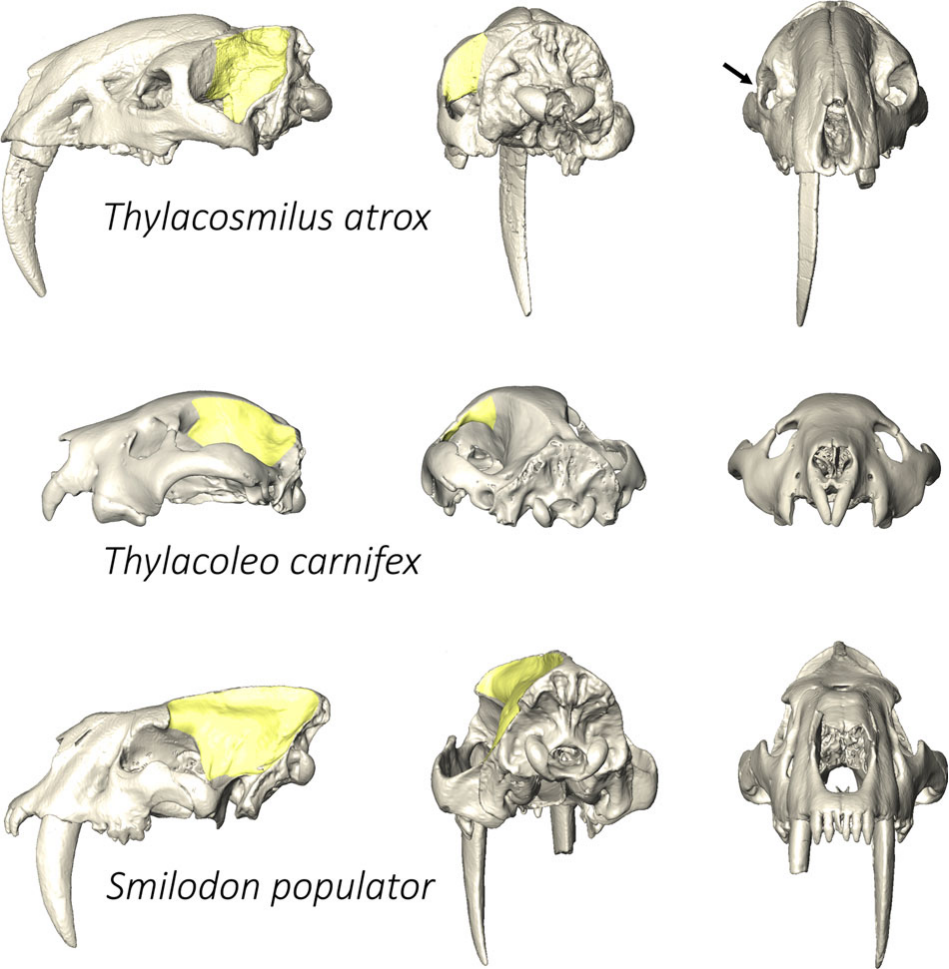
Postorbital bar surface (yellow) in *Thylacosmilus atrox* (FMNH P14531), *Thylacoleo carnifex* (SAM P16730), and *Smilodon populator* (MACN-PV 18057). Arrow indicates inward crushing of right orbit of FMNH P14531, not present on other side (artificially reconstructed).

Similarity in orbital orientation of *Thylacosmilus* and artiodactyls deserves special comment. This is very unexpected because, apart from the presence of an ossified postorbital bar, they have no other distinctive similarities in terms of cranial anatomy, phylogenetic relations, diet, or locomotor category. Some other mammals (e.g., felids, herpestids, primates, extinct *Thylacoleo*; e.g.,^11,38^) possess a postorbital bar that acts as a baffle, helping to prevent deformation of the eyeballs by isolating them from contraction of the temporalis muscles on the adjacent cranial sidewalls^1,6,38^. These taxa share presence of highly convergent orbits, unlike *Thylacosmilus* and artiodactyls. Nonetheless, the need for an ossified postorbital bar is not solely dependent on the structure of the orbits, but rather the orientation of the temporal fossa and its contained muscle: at a certain angle, temporalis contraction may induce deformation of the eyeball in the absence of a rigid osseous barrier^11,24^. The postorbital bar of *Thylacosmilus*, unlike that of *Thylacoleo* (Fig. 3), is high, wide, pneumatized, and mainly formed by the frontals rather than by the frontals and jugals in equal proportion as in *Thylacoleo*. These taxa are the only two metatherians having a postorbital bar, but their bars differ in design. In *Thylacosmilus*, not only does the postorbital bar contribute to the surface for temporalis muscle attachment (^39,40^; but see^38^ for contrary view), but it also provides a robust posterior orbital wall for resisting deformation and the effects of head movements in general.

Low convergence is not the only unexpected feature of the visual system of *Thylacosmilus*. Other measurable aspects of its orbital configuration (degree of verticality, frontation, orbitotemporal, and orbitolabyrinth angles) are not seen in this specific combination in any other therian carnivores (Fig. 2). A possible explanation is that these attributes are actually compensatory, a mechanism for retaining as much binocularity as possible in an otherwise severely modified cranium. Thanks to its exceptionally vertical and frontated gaze, *Thylacosmilus* would have been able to achieve a higher degree of binocularity than would ordinarily be possible for a species having such low orbital convergence. Compared to the probable condition of the skull in NT sparassodonts, *Thylacosmilus* sacrificed a primitively significant degree of convergence by laterally displacing the eyes, but made up for it by reorienting the bony orbits within the cranium whilst retaining the habitus of a hypercarnivore. Other thylacosmilids such as *Patagosmilus* exhibit less extreme versions of the evergrowing sabertooth canine: sockets are limited to the premolar section of the maxilla, and highly derived cranial modifications are absent^41^.

The factors that prompted orbital reorganization in *Thylacosmilus* are unknown, but some logical cause-effect connections can be made by comparing *Thylacosmilus* to other sabertooth placentals (*Smilodon*, *Homotherium*, *Barbourofelis*). These are the only other predaceous mammals in the comparative set that resemble *Thylacosmilus* (albeit to a less extreme degree) in exhibiting low orbital convergence coupled with high frontation and verticality angles, an almost orthogonal head posture in relation to orbital position (orbitolabyrinth angle), and hypertrophied canines. One possibility is that canine enlargement in sabertooth mammals imparts a physical constraint on the size and position of the orbits and the adjacent rostrum, thereby influencing the degree of orbital convergence and related angles (see also^42^). Conditions in *Thylacosmilus* may have been carried to an extreme over anything seen in sabertooth placentals because the canine was not only enlarged, but also evergrowing and structurally invasive in other areas of the skull. The root of the canine in *Smilodon populator* ends at mid-orbit and is closed, whereas in *Thylacosmilus* it extends to the back of the head, far beyond the orbit and is unrooted^16^. Although there are no quantitative data on snout growth in *Smilodon* (but see^43,44^), it is predictable that, as in other placentals, canine growth should have ended at or after complete eruption of the canine crown. Presumably, the maximum width of the snout was achieved at the same time. *Thylacosmilus* was under no similar constraint: continuing growth of the canines through early postnatal life could have further displaced the orbits laterally, producing a more divergent orbit orientation. This growth trajectory was compensated for by higher frontation and verticality angles, finally yielding a configuration quite unlike that of any other known mammal in which the eyes were positioned as in an artiodactyl but oriented in such a way that 3D vision was not lost.

In their combination, orbital traits of *Thylacosmilus*—an extinct South American sparassodont often called the “marsupial sabertooth”—have no equivalent in any known metatherian or eutherian. Because existing active predators exhibit highly convergent orbits, it has long been thought that this factor is basic to 3D binocularity. Although classified as a hypercarnivore, *Thylacosmilus* lacks highly convergent orbits; instead, it resembles perissodactyls and artiodactyls in having orbital positions fitted for panoramic vision. This is not as restrictive as it might seem, as the skull of *Thylacosmilus* exhibits countervailing adaptations (high orbital frontation and verticality) that partly compensate for lack of good convergence. These bizarrely contrasting adaptations in an active predator appear to be developmental: maxillofacial ontogeny in *Thylacosmilus* would have been dominated by the growth of its hypertrophied canines, the roots of which (unlike those of placental sabertooths) were evergrowing and, in the adult, extended over the top of the skull. Massive snout and canine growth during ontogeny would have resulted in relative lateral displacement of the orbits compared to the primitive sparassodont condition, in which this growth pattern did not occur. In compensation, the eyes and head posture were reoriented to preserve some degree of stereoscopy. Other correlated changes included the telescoping of the neurocranium to reduce the potential size of the temporal fossa, thereby inducing the development of a postorbital bar to increase attachment area for the temporalis muscle while at the same time preventing deformation of the highly frontated eyeballs.

Evergrowing canines do not occur in any other metatherian clade, and there is no evident explanation for its appearance in this single lineage. The fact that *Thylacosmilus* persisted from the Late Miocene through the mid-Pliocene suggests that, whatever its predatory behavior, it was not seriously impeded by reduced binocularity. Primates may have developed excellent stereoscopy at the expense of the olfactory apparatus^1^, but this is not the model followed by placental and marsupial carnivores, both of which possess good 3D vision and excellent olfaction.

## Materials and methods

The orbital regions of two *Thylacosmilus atrox*^17^ (FMNH P14531 and MMP 1433-M) were compared to the orbital regions of other sparassodonts, the extinct marsupials *Thylacinus cynocephalus*^45^ and *Thylacoleo carnifex*^46^, extant marsupials, and representative sabertooth mammals (*Smilodon populator*^47^, *S*. *fatalis*^48^, and *Homotherium serum*^49^) (list of material in Supplementary Methods). Species, collection numbers and Computed Tomography (CT) parameters for the specimen studied are listed in Supplementary Table S1. Some specimens were downloaded as CT scans or mesh models from Digital Morphology (http://digimorph.org), MorphoSource (http://morphosource.org), Digital Morphology Museum KUPRI (http://dmm.pri.kyoto-u.ac.jp), L. Witmer^23^ and S. Wroe^50^ (complete references in Supplementary Methods). Tomographies were segmented with 3DSlicer^51^ and some new mesh models are available through MorphoSource (project 000493868, morphosource.org) or from the museum in which the specimens are deposited.

To quantify orientation, the orbit is reduced to a plane defined by three landmarks (Supplementary Table S2). Landmarks are taken directly on the 3D models generated with 3DSlicer^51^. Angles and other measurements are calculated in Excel from landmark coordinates (equations in Supplementary Methods; landmarks in Supplementary Data 2) and verified by 3D measurements in 3-matics Research 13.0 (Materialise, Leuven, Belgium). All results are given in Supplementary Table S3 and S4; see also Figs. 1 and 2 and Supplementary Figs. S2–35. Fossil deformation was quantified as the angle between the sagittal plane and the palatal, frontal, and basal planes (see Supplementary Methods). Specimens with residual angles >10° (one orbit only) and 25° (both orbits) were excluded from the analysis.

When both orbits can be measured in one specimen, the mean values of angles are reported as a procedure to correct for fossil deformation. The two specimens of *Thylacosmilus* (FMNH P14531 and MMP 1433-M) exhibit notable differences in mean values. Because the holotype is exquisitely preserved, its values are regarded as being somewhat more accurate. However, some deformation is certainly present (arrow, Fig. 3), and we acknowledge that reconstruction of the right zygomatic arch added error to measurements.

To quantify the orientation of the orbit in relation to the labyrinth, the orbitolabyrinth angle was introduced to measure the dihedral angle between the orbital plane and the ipsilateral lateral semicircular canal of the labyrinth. The lateral semicircular canal was chosen because its orientation is thought to be related to habitual head posture and gaze (e.g.,^52–55^; but see^56^), and for that reason is used as a proxy for head orientation in fossil taxa (e.g.,^57–59^; but see^60^). Landmarks defining the lateral semicircular canal are taken at the center of the lumen (see Supplementary Fig. S1 and Methods). Because the CT scan of the holotype of *Thylacosmilus atrox* (FMNH P14531) was too coarse to allow accurate reconstruction of the semicircular canals, we used the reconstruction of the labyrinth of the paratype (FMNH P14344) as a proxy, superimposing it on the holotype (see also^59^) to permit an estimate of the orbitolabyrinth angle.

### Reporting summary

Further information on research design is available in the Nature Portfolio Reporting Summary linked to this article.

## Supplementary information

Supplementary Information

Description of Additional Supplementary Files

Supplementary Data 1

Supplementary Data 2

Reporting Summary

## Data Availability

The dataset analyzed during this study is available in Supplementary Data 1 and 2. Some new mesh models are available through MorphoSource (project 000493868, morphosource.org) or from the museum in which the specimens are deposited.
