# Supplementary material for: Seeing through the eyes of the sabertooth Thylacosmilus atrox (Metatheria, Sparassodonta)
Source: Commun Biol. 2023 Mar 21;6:257. doi: 10.1038/s42003-023-04624-5 (PMC10030895; doi:10.1038/s42003-023-04624-5)
Supplement: Supplementary file 1 — Supplementary Information [file 42003_2023_4624_MOESM1_ESM.pdf]

Supplementary Information for
Seeing through the eyes of the sabertoooth *Thylacosmilus atrox* (Metatheria, Sparassodonta).

Charlène Gaillard, Ross D. E. MacPhee, and Analía M. Forasiepi

This PDF file includes:

- Supporting text
- Figures S1 to S35
- Tables S1 to S4
- Legends for Supplementary Data 1 and 2
- References

Other supporting materials for this manuscript include the following:

- Supplementary Data 1
- Supplementary Data 2

Supplementary Note 1

Sparassodonta. Sparassodonta is an extinct group of native predaceous metatherians from South America. There are more than 60 currently valid species, with taxa covering different body sizes (from less than 1kg, such as *Pseudonotictis chubutensis,* to more than 200kg, such as *Proborhyaena gigantea;* [1–3]) and locomotor types (from scansorial, such as *Sipalocyon gracilis* and *Prothylacynus patagonicus,* to terrestrial with incipient cursoriality, as suggested for *Borhyaena tuberata* and *Thylacosmilus atrox*; [4–10]). Craniodental features of all sparassodont species are consistent with general carnivory; most of them are consistent with hypercarnivory[1,3,11–16]. Together with other non-mammalian taxa, such as crocodiles (Sebecidae), "terror birds" (Phorusrhacidae), and some snakes (Madtsoiidae), as well as with placental carnivores and didelphimorphian marsupials, sparassodonts occupied a major role in the predatory guild of the South American terrestrial ecosystems through the Cenozoic[1,2,12,16,17].

Early discoveries of sparassodonts date to the late 19th century[18], when Cenozoic outcrops in Patagonia were first being exploited. Among the first species to be characterized was *Borhyaena tuberata,* a taxon that later became iconic for the group, and *Cladosictis patagonica*, the most abundant sparassodont species in collections (e.g.,[19–21]). *Borhyaena* spp. and about 20 other closely related species compose Borhyaenoidea. This includes medium to large-sized sparassodonts with marked disparity in the cranial, dentition, and postcranial morphology. Some taxa were slender and fox-like, such as *Lycopsis longirostrus* (from the middle Miocene of La Venta, Colombia; [22]), others were massive and robust, bear-like in appearance, such as *Arctodictis munizi* (from the early Miocene of Patagonia, Argentina; [23]). Others had outlier morphologies, such as *Thylacosmilus atrox* with its hypertrophied sabertooth canines, the species that is the core of this contribution. Species of *Cladosictis* and 20 other species represent Hathliacynidae. These were small to medium-sized sparassodonts, with long and slender skulls and dentaries, and fox- or weasel-like appearance (e.g.,[14,20,24,25]). These two major taxonomic groups (Borhyaenoidea and Hathliacynidae) in

addition to some stem taxa (see below) comprise Sparassodonta.

The term Sparassodonta was coined by Ameghino[26] and classically it has been considered a natural group (e.g.,[27–30]). Early controversies concerned interpretation of its phylogenetic position within metatherians (e.g.,[11–14,24,28,31–36]); however, currently there is agreement in treating them as a group of stem marsupials (e.g.,[37–46]).

The definition of the Sparassodonta has recently been affected by some controversial claims. Species of *Patene* (from Eocene deposits of Argentina, Brazil, and Peru) have been traditionally considered as possessing the most primitive morphology found in the group (e.g.,[14,47–50]). In line with this, recent phylogenetic analyses define Sparassodonta as the group that includes the common ancestor of *Patene* species, *Borhyaena* (Borhyaenoidea) and *Cladosictis* (Hathliacynidae) and all its descendants (e.g.,[41–46]; see[51] for discussion).

Mayulestes ferox and *Allqokirus australis* from the Early Paleocene of Tiupampa, Bolivia (e.g.,[35,36,45,52]) have recently been recovered in the same monophyletic clade as *Patene* at the base of Sparassodonta[45,46]. However, at least in the case of *Mayulestes*, alternative phylogenetic positions have been also proposed[39–44,53–55]), and despite its stem position to the common ancestor of *Patene* and other sparassodonts, the Tiupampan species has nevertheless been named by some authors as a sparassodonta (e.g.,[55]).

Similarly, species of *Varalphadon* from the Late Cretaceous of Utah and Alberta were claimed by Carneiro[56] to be members of Sparassodonta (alternative phylogenetic interpretations presented by Johanson[57]; [58]), which requires the conclusion that sparassodonts have a biochron that extends to the Mesozoic and a distribution that includes North America. The result of Carneiro´s[56] phylogenetic analysis unambiguously shows *Varalphadon* in a stem position relative to a clade formed by *Allqokirus australis, Patene simpsoni,* plus other sparassodonts[56], while the name Sparassodonta was attached to a node that includes all those taxa. Carneiro[56] used a selection of characters to define Sparassodonta (but see [45]); however, definitions based on characters are ambiguous since they are likely to rapidly change with new discoveries and the inclusion of new taxa and data in

phylogenetic analyses (e.g.,[59–61]). To maintain stability, we utilize a node-based definition of Sparassodonta that concurs with the traditional conception of its bounds. Based on this and current knowledge, Sparassodonta is restricted to South America with a fossil record that extends from the early Paleocene or early Eocene to the mid-early Pliocene[11–14,24,36,41,45,52,62–65].

Thylacosmilus atrox. Among sparassodonts, *Thylacosmilus atrox* is remarkable for its numerous differences from other members of its group (see main text). It resembles sabertooth felids (particularly *Barbourofelis*; [66]) in having large hypertrophied upper canines, this being regarded as a classic example of convergent evolution (e.g.,[11,67–72]; but see [73]). Other distinctive features include, in the skull, a massive snout, anteorbital fossa, ossified postorbital bar, strong sagittal, temporal, nuchal, and supraglenoid crests, ossified auditory bulla, and small nasals lacking contact with frontals in dorsal view and partially covered by the maxilla; mandibles with a subvertical symphyseal flange, shallow ramus, small but deep masseteric fossa, poorly inflected angle, low condyle in relation to the alveolar plane; upper canine saber-like, ever-growing, and deeply anchored in the maxilla, premolars reduced in size and number, single rooted first upper premolar (homologous P2), jugal tooth series bowed, and simplified molar structures favoring shearing function, which suggest a highly specialized hypercarnivorous diet ([11,51,65,71,74–76], see also main text). Hypercarnivory applies to species that feed mostly on other vertebrates (cf.[77]). For dietary reconstruction in fossil mammals, morphometric indexes are used; one measures the relative grinding area (RGA) of the most carnassial molar (e.g.,[78]; see also [1,3,15,16]). For *Thylacosmilus*, the RGA index has a minimum of 0 since molars lacks grinding areas (talonids lacking in lower molars). Other dietary reconstructions for *Thylacosmilus* have been made by using geometric morphometric and allometric and discriminant analyses of mandibular shape[79] and molar morphology[16], both providing similar conclusions. *Thylacosmilus* was a hypercarnivorous predator, with a large maximum gape angle (>100°; [80]) but with a bite force extremely low compared to other sparassodonts and lower than other placental hypercarnivores[80–82].

The locomotor behavior of *Thylacosmilus* has been interpreted as terrestrial with incipient cursoriality[6,7,10,68], which as an ambush predator may have facilitated its pursuit strategy[6,8,65]). There is a marked size difference among the known specimens of the species that likely suggest intraspecific variation (e.g.,[51,65]). Estimations of body mass are broad and depend on the specimens analyzed and methods. The range, probably involving both over- and underestimation, is from 150 kg[83] to less than 30kg[84], with a mean for the species of ~100kg[85].

Several studies have attempted to construct a functional predation model for *Thylacosmilus atrox* in comparison to sabertooth cats. Currently, most studies support a stabbing model, in which the primary force applied to the canines is neck driven (e.g.,[6,8,70,72,80,81]), rather than the primary force coming from the jaw adductors as in sabertooth cats (i.e., the canine-sharing bite model following[80,81]). The unique paleobiology of *Thylacosmilus* has no clear counterpart among living or fossil predators[73], as we also demonstrate here in relation to the analysis of its vision (see main text).

Thylacosmilus atrox is represented by a number of specimens collected from a range of late Miocene localities: Paraná (Entre Ríos Province, Argentina); Salinas Grandes de Hidalgo, Quehué and El Guanaco (La Pampa Province, Argentina), Carhué (Buenos Aires Province, Argentina), Chiquimil (Catamarca Province, Argentina), Arazatí (Uruguay), and the early Pliocene: Monte Hermoso (Buenos Aires Province, Argentina), Puerta del Corral Quemado (Catamarca Province, Argentina); Barranca de Los Lobos and Chapadmalal (Buenos Aires Province, Argentina), Valle de Nono (Córdoba Province, Argentina)[11,51,65,67,68,75,86–90]. Most of these specimens consist of fragmentary material and isolated teeth. The best-preserved remains are the holotype (FMNH P14531) and paratype (FMNH P14344), originally described by Riggs[67,68] and specimen MMP 1443-M first described by Goin and Pascual[65].

The family Thylacosmilidae ([91]; see also [67,68] ranked as subfamily) was originally created to include only the genus *Thylacosmilus* (by then including two species *Thylacosmilus atrox* and *Thylacosmilus lentis*; but see [65]). Later Goin[74] described *Anachlysictis gracilis* from middle Miocene outcrops of La Venta

(Colombia), and Forasiepi and Carlini[76] named *Patagosmilus goini* from middle Miocene Rio Chico locality (Argentina). *Anachlysictis* is known only from a lower jaw with dentition, a very fragmentary frontal bone, and a partial postcranial skeleton, while *Patagosmilus* is represented by a partial cranium and a few postcranial elements. Both species have a morphology that is more primitive than that of *Thylacosmilus.* Derived features shared between *Thylacosmilus* and *Anachlysictis* are as follows: large, subvertical symphyseal flange with radially-oriented lingual bony striations; alveolar and ventral edges of the dentary ramus subparallel and straight; low and poorly developed masseteric crest; poorly inflected angle; low condyle in relation to the alveolar plane; bowed jugal series, and two lower premolars (likely homologous to p2-p3)[74,92]. Derived features shared between *Thylacosmilus* and *Patagosmilus* are: short braincase length, bowed postcanine tooth row, two upper premolars (likely homologous to P2-P3), last upper premolar with complex morphology (interpreted as retained DP3 in the adult molar series), and hypertrophied ever-growing upper canine[76]. Phylogenetic analysis has recovered *Patagosmilus*[42,43,55] or *Patagosmilus* plus *Anachlysictis*[92] in the same monophyletic group as *Thylacosmilus.* For the node-based definition of Thylacosmilidae, we claim the group that includes the common ancestor of *Thylacosmilus atrox*, *Patagosmilus goini*, and *Anachlysictis gracilis* plus all its descendants (e.g.,[2]). Thylacosmilidae is known from the middle Miocene to mid-early Pliocene of South America.

In addition to the records already considered, one or two other putative thylacosmilids have been discovered in the middle Miocene of La Venta[74,92], with a much more generalized morphology than other thylacosmilids. This Laventan taxon could either represent a stem or basal thylacosmilid[92], or alternatively a different sparassodont lineage with incipient and convergent sabertooth architecture[74]. To this record can be added an isolated upper molar from the early Miocene of Patagonia[93], but this specimen has not yet been included in a phylogenetic framework.

In a recent contribution, Engelman et al.[55] suggested a major systematic modification, reducing *Thylacosmilus* and its kin to subfamily level

(Thylacosmilinae, instead of family Thylacosmilidae), and re-defining the group using a stem-based definition. Under this proposal, the group includes all sparassodonts more closely related to *Thylacosmilus atrox* than to *Proborhyaena gigantea*, *Borhyaena tuberata*, *Prothylacynus patagonicus*, *Lycopsis torresi*, *Cladosictis patagonica*, or *Sipalocyon gracilis*[55]. On this basis their new taxon *Eomakhaira molossus* from the early Oligocene of Cachapoal locality, central Chile, was included in Thylacosmilinae[55], but this is also the case of other sparassodonts (e.g., *Callistoe vincei*, *Paraborhyaena boliviana*) traditionally considered Proborhyaenidae and which in some phylogenetic arrangements (e.g.,[94]; [43]: Figure 7; [44]: Figure 3; [45]: Figure 29; [46]: Figure 49) are closer to *Thylacosmilus* than to any other sparassodont included in those studies. The phylogenetic relationships of the taxa originally named Proborhyaenidae are still unsettled with several alternative propositions according to different authors and data sets: not only they are shown as monophyletic, but also paraphyletic close to thylacosmilids (e.g.,[40,43–46,55,92,94]; for discussion see also [2,9,64]). In view of these uncertainties regarding the arrangement of proborhyaenids, and in order to avoid discrepancies for the sabertooth group, we continue to follow the more conservative node-based definition of Thylacosmilidae.

Supplementary Materials

In this study the orbital region of three crania of the non-marsupial metatherian *Thylacosmilus atrox*[67] (FMNH P14531, MMP 1433-M, and MLP 35-X-4-1) is compared to the orbits of several other sparassodonts: *Cladosictis patagonica*[18] (MACN-A 5927, MPM-PV 3645, MPM-PV 4323, MPM-PV 4326), *Sipalocyon externus*[95] (MACN-CH 1911), *Sipalocyon gracilis*[18] (AMNH VP-9254), *Sipalocyon* sp.[18] (MPM-PV 4316), *Pharsophorus* sp.[96] (MCNAM 4957), *Arminiheringia* sp.[97] (MLP 82-V-1-1), *Arctodictis sinclairi*[12] (MLP 85-VII-3-1), and *Borhyaena tuberata*[18] (MPM-PV 3625, MPM-PV 4380). In addition, we studied the orbital orientation of the extant didelphid *Didelphis virginiana*[98] (TMM M-2517) and *Chironectes minimus*[99] (MACN-Ma 24300), the dasyurid *Dasyurus hallucatus*[100]

(TMM M-6921), *Dasyurus maculatus*[98] (UMZC A6.10/3, ark:/87602/m4/M68239), *Sarcophilus harrisii*[101] (IMNH R-1368, ark:/87602/m4/M118878), the extinct thylacinid *Thylacinus cynocephalus*[102] (NMB c.2526), the extinct diprotodont *Thylacoleo carnifex*[103] (SAM P16730), as well as the extant felids *Panthera leo*[104] (MVZ 117849, PRICT 1434, and PRICT 1435) and the saber-toothed fossil felids *Smilodon populator*[105] (MACN-PV 18057), *Smilodon fatalis*[107] (AMNH FAM14349, ark:/87602/m4/M9729), and *Homotherium serum*[106] (TMM 933-3444).

Supplementary Methods

Tomographies. Scanning of AMNH VP-9254 was performed on the GE Pheonix vtomex s240 µCT scanner housed at the AMNH facility in New York, USA. MACN-CH 1911 digitalization was performed on the GE Phoenix vtomex L240-180 µCT scan housed at the X-Ray Tomography Imagery Platform AST-RX MNHN in Paris, France. NMB c.2526 was scanned on the microCT Bruker Skyscan 1275 housed at the Biomaterials Science Center of the University of Basel in Switzerland. Digitalization of MACN-A 5927, MACN-PV 18057, MPM-PV 3625, MPM-PV 3645, MPM-PV 4316, MPM-PV 4323, MPM-PV 4326, MPM-PV 4333, MPM-PV 4380, MLP 35-X-4-1, MLP 82-V-1-1, MLP 85-VII-3-1 were performed on the Philips Brillance 64 tomograph of the Equipo de Neurocirurgía Endovascular Radiología Intervencionista housed at the Clínica La Sagrada Familia in Buenos Aires, Argentina. MMP 1433-M was scanned on the Philips Brilliance 64 tomograph house at the Instituto Radiológico in Mar del Plata, Argentina. Scanning of MACN-Ma 24300 was performed on the microtomograph assembled in house at the Facultad de Matémática, Astronomía, Física y Computación of the University of Córdoba in Argentina. MCNAM 4957 was scanned at the tomograph housed at the Fundación Escuela de Medicina Nuclear in Mendoza, Argentina. Other specimens were downloaded as Computed Tomography (CT) scan or mesh models from Digital Morphology (http://digimorph.org; TMM M-2517[108], TMM M-6921[109], TMM M-3444[110], MVZ 117849[111]), MorphoSource (http://morphosource.org; UMZC A6.10/3[112], IMNH R-1368, AMNH FAM14349[113]), Digital Morphology Museum

KUPRI (http://dmm.pri.kyoto-u.ac.jp; PRICT 1434, PRICT 1435), L. Witmer (FMNH P14531, FMNH P14344; see [80]), and S. Wroe (SAM P16730; see [114]). The tomographies were segmented with 3DSlicer[115].

Orbital orientation. To quantify orbital orientation, the orbit is reduced to a plane defined by three landmarks. There are inconsistencies in previous methods regarding the choice and location of the landmarks, which vary according to the orbital anatomy of the group studied and the quality of the material available. To be able to compare our measurements to the data in the literature, we built four orbital planes for each orbit, using landmarks provided by four methods (Table S2). The landmark coordinates for the 28 specimens studied are given in Supplementary Data 2. The 20 species of this study are included for graph representation to the 214 species from the databases of Heesy[116,117], 68 species from Finarelli and Goswami[118], 17 species from Pilatti and Astúa[119], and 109 species Casares-Hidalgo et al.[120].

The issue of using only three landmarks to build a plane when studying fossils is not new (e.g., Finarelli and Goswami[118] used 6 landmarks to construct the sagittal plane in order to be able to find at least three of them preserved on a given fossil). Regarding the sagittal plane, we chose not to define three homologous landmarks. Because mammals have bilateral symmetry, any point on the sagittal crest, or the mid-sagittal sutures of the premaxilla, maxilla, nasals, palatine, and basicranium, can be substituted in order to construct the midsagittal plane when one or more of the defined landmarks are not available.

Anatomical landmarks developed from the primate literature are frequently adapted for use with for other mammals, although they are not always truly homologous (see Discussion on the methods). In the case of *Thylacosmilus atrox*, for example, the nasion (midsagittal point of contact between the frontals and nasals) is obstructed by maxillary overlap of the nasals. The midsagittal point of contact between nasal and maxilla was taken as an analogous landmark because of its similar position to the nasion of other metatherians.

For specimens that do not have visible sutures, all landmarks defined by sutural contact are obviously problematic. To deal with this we searched for applicable sutures on other specimens of the same species or descriptions in the literature; this enabled translation of landmarks to the 3D mesh models of problematic specimens (e.g., *Cladosictis patagonica* MPM-PV 4323; *Sipalocyon* sp. MPM-PV 4316; *Chironectes minimus* MACN-Ma 24300, *Panthera leo* MVZ 117849). Other problems related to landmark definitions are addressed below in Discussion of the methods.

Landmarks are taken directly on the 3D model in 3DSlicer [115] and their coordinates imported, together with the 3D model, in 3-matics Research 13.0 (Materialise, Leuven, Belgium). Angles are automatically calculated in Excel (see equations below) and later verified by 3D measurements in 3-matics Research 13.0. Using equations previously provided in the literature (e.g.,[116,119]), each plane is expressed by the equation $n_1x + n_2y + n_3z + d_n = 0$, where

$$\overrightarrow{n_1} = \begin{pmatrix} n_1 \\ n_2 \\ n_3 \end{pmatrix} = \begin{pmatrix} y_A(z_B - z_C) + y_B(z_C - z_A) + y_C(z_A - z_B) \\ z_A(x_B - x_C) + z_B(z_C - z_A) + z_C(z_A - z_B) \\ x_A(y_b - y_C) + x_B(z_C - z_A) + x_C(z_A - z_B) \end{pmatrix}$$ is the normal vector

of the plane and A(x_A, y_A, z_A), B(x_B, y_B, z_B), and C(x_C, y_C, z_C) are three landmarks belonging to the plane. All dihedral angles between two planes are calculated as the angle between the normal vectors of the two planes involved (see Table S1) in the equation:

$$\alpha = \cos^{-1}\left(\frac{\vec{n}_1 \cdot \vec{n}_2}{\|n_1\|\|n_2\|}\right) = \cos^{-1}\left(\frac{n_1m_1 + n_2m_2 + n_3m_3}{\sqrt{{n_1}^2 + {n_2}^2 + {n_3}^2}\sqrt{{m_1}^2 + {m_2}^2 + {m_3}^2}}\right)$$

where $\overrightarrow{m_1} = \begin{pmatrix} m_1 \\ m_2 \\ m_3 \end{pmatrix}$ is the normal vector of the second plane expressed as

$m_1x + m_2y + m_3z + d_m = 0.$

For orbital frontation as measured by Casares-Hidalgo et al.[120], the dorsal plane is built as a plane perpendicular to the sagittal plane[120] and passing through the inion I(x_I, y_I, z_I) and nasion N(x_N, y_N, z_N). In the absence of a third landmark,

the normal vector of the dorsal plane is defined as the cross product of the normal vector of the sagittal plane and the direction vector of the inion-nasion,

$$\vec{d} = \begin{pmatrix} d_1 \\ d_2 \\ d_3 \end{pmatrix} = \begin{pmatrix} s_2 NI_3 - NI_2 s_3 \\ s_3 NI_1 - NI_3 s_1 \\ s_1 NI_2 - NI_1 s_2 \end{pmatrix}, \text{ where } \overrightarrow{NI} = \begin{pmatrix} NI_1 \\ NI_2 \\ NI_3 \end{pmatrix} = \begin{pmatrix} x_N + x_I \\ y_N + y_I \\ z_N + z_I \end{pmatrix} \text{ and } \overrightarrow{s_1} = \begin{pmatrix} s_1 \\ s_2 \\ s_3 \end{pmatrix}$$

(calculated as the normal vector of a plane, see equation above). The result for orbital frontation is calculated as the angle between the normal vector of the dorsal plane ($\vec{d}$) and one on the orbital plane ($\vec{o}$).

Orbit frontation measured by the method of Heesy[117] is calculated as the angle between the direction vector of the nasion-inion $\overrightarrow{NI}$ and the direction vector of the line of intersection between the orbital and sagittal plane ($\overrightarrow{so}$). This is found by resolving the system of equations of the two corresponding planes such as

$$\begin{cases} n_1 x + n_2 y + n_3 z + d_n = 0 \\ m_1 x + m_2 y + m_3 z + d_m = 0 \end{cases} \Rightarrow \begin{cases} x = -\left(\frac{n_3}{n_1} + \frac{n_2 m_3 n_1 - n_2 n_3 m_1}{n_1 (n_2 m_1 - m_2 n_1)}\right) t + \frac{n_2 d_n m_1}{n_1} - n_2 d_m - \frac{d_n}{n_1} \\ y = \left(\frac{-n_3 m_1 + m_3 n_1}{n_2 n_1 - m_2 n_1}\right) t - d_n m_1 + d_m n_1 \\ z = t \end{cases}.$$

Thus $\overrightarrow{so} = \begin{pmatrix} so_1 \\ so_2 \\ so_3 \end{pmatrix} = \begin{pmatrix} -\left(\frac{n_3}{n_1} + \frac{n_2 m_3 n_1 - n_2 n_3 m_1}{n_1 (n_2 m_1 - m_2 n_1)}\right) \\ \frac{-n_3 m_1 + m_3 n_1}{n_2 n_1 - m_2 n_1} \\ 1 \end{pmatrix}$. The angle between the two direction vectors is calculated following the equation as the angle between two normal vectors (equation given above).

Orbital convergence for *Thylacosmilus atrox* and *Thylacoleo carnifex* cannot be measured with the landmarks provided by Pilatti and Astúa[119] because of the presence of the postorbital bar (postorbital process of the frontal bone = tip of the frontal process of the jugal).

Orbitolabyrinth angle. The plane of the lateral semicircular canal is built by three landmarks taken at the center of the lumen (Figure S1): at the exit of the canal from the ampulla [1], at mid-curve of the canal [2], and at the connection of the canal to the vestibule, or, in cases where a secondary crus commune is present (e.g., *Didelphis*, Figure S1) before its junction to the posterior semicircular canal [3]. The orbitolabyrinth angle is calculated as the mean value of the dihedral angle

between the orbital plane and the lateral semicircular canal of the labyrinth on the same cranial side.

The CT scan of the holotype of *Thylacosmilus atrox* (FMNH P14531) does not allow reconstruction of the semicircular canals at the necessary level of resolution. The canals are however well seen on the CT scan of the paratype (FMNH P14344), for which labyrinths were reconstructed. The cranium of the paratype, together with the reconstructed labyrinths are scaled and superimposed to best fit with the cranium of the holotype[51], which permits calculation of the orbitolabyrinth angle of *Thylacosmilus atrox*. Such combined reconstruction of specimens implies a greater error but is the only way to measure orbitolabyrinth angle for *Thylacosmilus atrox* in the absence of better scan quality. In fact, among the 28 specimens studied, only six could have their labyrinth reconstructed virtually. Importantly, orbitolabyrinth angle gives the orientation of the gaze in relationship to head posture based on the horizontal position of the lateral semicircular canal.

Other measurements. Distances between two landmarks are calculated as $\sqrt{(x_2 - x_1)^2 + (y_2 - y_1)^2 + (z_2 - z_1)^2}$, with ($x_1$, y_1, z_1) and (x_2, y_2, z_2) corresponding to the coordinates of the two landmarks involved in the distance, following Heesy[117] for the interorbital width and Pilatti and Astúa[119] for skull length and snout length. The caudal landmark used to measure snout length is taken at the mid-point between the left and right orbitale anterius and its coordinates are calculated as $\begin{pmatrix} \frac{x_{leftOA}+x_{rightOA}}{2} \\ \frac{y_{leftOA}+y_{rightOA}}{2} \\ \frac{z_{leftOA}+z_{rightOA}}{2} \end{pmatrix}$. Snout width is calculated as the distance between the left and right most anterior point on the border on the medial wall of the infraorbital foramen. Relative rostrum length and rostrum proportional width follow Pilatti and Astúa[119].

In an attempt to quantify evident deformation, the angle between the sagittal plane and the palatal, frontal, and basal plane is calculated and subtracted from 90° (the expected angle between the respective planes in non-deformed

specimens). In extant specimens, all residual angles deviate less than 5°. In the fossils selected, the residual angles are generally less than 10°, with the exception of three specimens: *Thylacosmilus atrox* MMP 1433-M, *Cladosictis patagonica* MPM-PV 4326, and *Sipalocyon* sp. MPM-PV 4326. We arbitrarily selected specimens that have all residual angles less than 25° or less than 10° for specimens that only have one orbit preserved. With regard to *Thylacosmilus atrox* MMP 1433-M and FMNH P14531, there are large differences between the angle values of the two specimens, surely due to the larger amount of deformation in MMP 1433-M. FMNH P14531 is regarded as less deformed and better representing the values of the species, despite exhibiting some degree of deformation on the right side and artificial reconstruction on the other (arrow, Figure 3).

Orbital convergence, verticality, and frontation, as well as orbitotemporal angles are mostly taken as mean values between the left and right orbits (when possible) in order to balance lateral deformation of the skull. This ideal unidirectional deformation does not represent the real deformation of the fossils, but gives at least an estimate of the tolerated angle difference for selecting specimens (see above). Residual angle test could not be measured for Casares-Hidalgo et al.'s[120] method because the dorsal plane is constructed as perpendicular to the sagittal plane and hence has no residual values.

Although variation between the left and right orbit in fossil specimens is interpreted to come mainly from deformation, some degree of bilateral asymmetry cannot be dismissed. *Thylacoleo carnifex* is interesting in that regard because the left postorbital bar is complete, whereas the right one has a tiny gap (<2mm). In this case, with no sign of breakage or rugosity, variation can be interpreted either as an artefact of model reconstruction, as deformation, or as true biological variation. Orbit orientation variation at the species level is also present[116,119,121,122] but cannot be taken into account quantitatively because it is impossible to dissociate from deformation in fossil specimens.

Virtual eye reconstruction. Virtual eye size for *Thylacosmilus atrox* and *Thylacoleo carnifex* is estimated following Rosenberger et al.[123] using 3-matic Research 13.0 (Materialise, Leuven, Belgium), from which diameter is directly extracted from the software. Because other sparassodonts lack the postorbital bar, their eyeball size could not be estimated. Eye diameters for extant specimens come from the literature ([124,125]; Table S3). To compare relative eye size, we calculate the ratio between mean eye diameter over skull length, rostrum relative length, and snout width.

Supplementary Discussion of the methods

Vision in fossil taxa is hard to estimate because vision-related soft tissues are lacking and osteological indicia are limited in various ways. For example, although the optic nerve (CNII) has its own foramen (=optic foramen) in almost all eutherians, giving potential information on their visual capacity (see [126]), metatherians possess only one large gap, the sphenorbital fissure, for the optic (CNII), oculomotor (CNIII), trochlear (CNIV), ophthalmic (CNV_1) and abducens (CNVI) nerves (e.g.,[41,127,128]. The bony orbit provides the only accessible area where information on the vision of fossil taxa can be gathered.

The orbit is traditionally quantified by orbital orientation and orbital size. Orbital orientation is characterized by three angles: convergence, verticality, and frontation. Convergence is the degree to which the left and right orbit are orientated forward[129]. It is calculated as the dihedral angle between the sagittal or mid-sagittal plane and the orbital plane[121]. In the literature, there is confusion between the terms “verticality” (defined by Heesy[116]) and “frontation” (defined by Cartmill[129]). In Heesy’s view[116] (see also [117,130]), verticality measures orbital orientation in relation to the palate, whereas orbital frontation measures it in relation to the braincase. However, various authors only use one of the terms (verticality in Pilatti and Astúa[119]; frontation in Finarelli and Goswami[118], Casares-Hidalgo et al.[120]) to quantify the orbit orientation in relation to the posture of the cranium in a general manner (see Table S2). Interestingly, but also adding to the confusion, “frontation”

is used by[131] to quantify the angle between the lateral semicircular canal and a plane constructed by the extraocular muscles. Relating orbital orientation to the orientation of the labyrinth has also been undertaken by Simpson and Graf[132] and Graf and Bruken[133]. Unfortunately, these methods cannot be used in the absence of muscles preserved on dry skulls.

To measure orbital orientation in this study, the orbit is reduced to a plane defined by three landmarks. We used four orbital planes for each specimen, following the landmarks provided in [117–120] (see Methods). We do not distinguish better or worse methods because all of them serve the purpose of this study and bring valuable information on various parameters related to mammalian vision. We would however like to highlight some of the issues encountered with each method because they have influence on the interpretation of results. As an example, orbital orientation does not correlate with visual strategy and habitat in one study[120], but they do in others[130,134]. Similarly, correlation between orbital orientation and encephalization is established in one article[134], contrasted in another[118] and dismissed in a third[119]. Therefore, interpretation will depend on, and cannot be considered independent of, the method selected.

In the literature, correlations resulting from the application of these methods are based on very disparate datasets, each focusing on distinct taxonomic group (Didelphidae[119]; Carnivora[118,120]; Mammalia[117,130]) and containing different fossil information (none[117,130]; little[118]; much[120]). Each method reflects special features of its dataset and might not always be applicable to a different dataset, as in the case of Pilatti and Astúa's method[119] which does not allow the construction of the orbital plane in specimens possessing a postorbital bar.

The confusion between the two terms "verticality" and "frontation" (see above) make them impossible to compare if they come from two different methods, not only because the orbital orientation is not measured in the same way, but also because the terms refer to different anatomical notions. For example, the orbitotemporal angle of Casares-Hidalgo et al.[120] should correspond in principle to Heesy's supplementary orbitotemporal angle[117]. However, these angles are in fact

not intercomparable because the two studies do not use the same landmarks to build the orbital plane.

Furthermore, and as illustrated in Figure 2 and Figures S2-S34, orientation of the orbits will vary significantly depending on the selection of the three landmarks used to define the orbital plane. As a result, between any two methods angles of orbital orientation can differ by as much as 40°. Clearly, measurements that are not based on the same parameters are simply noncomparable (see also [118]).

Finally, the following issues were encountered with the definition of the landmarks:

- **Heesy's method**[117]: The majority of the landmarks are type II landmarks that do not refer to truly homologous anatomical points[135]. They are harder to locate and reproduce, whether for one person or two (see [136]). The intraobserver error can be reduced by utilizing the mean value for measurements of the left and right orbits in all specimens of the same species (when available) (see [117]). Interobserver error is more complicated to control and we did not generate a dataset to quantify it. Simply as an example, we noted that orbital convergence of *Panthera leo* is 60° in our dataset, but 68° in Heesy's[117]. Part of the difference is due to intraspecific variation, as Heesy[117] showed with regard to significant variance between mean values of individuals. In fact, the three *Panthera leo* specimens from the present study have quite disparate mean values (between 57° and 66°). However, such differences surely also come from interobserver error (see also [137]).

  Landmarks utilized in the primate literature are problematic for investigations of non-primates. This is the case with nasion, defined as the midsagittal point of contact between the internasal suture and the frontal suture. Although all nasions are homologous by definition, the definition itself may not be applicable to highly derived forms with unusual morphologies, which requires that an analogical approach be taken. As already noted, in *Thylacosmilus atrox* the nasion is obstructed

by the maxilla overlapping the nasals. Strictly speaking the nasion does not occur in *Thylacosmilus* and we used an analogous landmark—contact of the nasal and maxilla—as a proxy for the nasion.

A similar issue came up with the landmarks taken at the level of the first upper molar as a means for representing the palatal plane. In marsupials, the first upper molar is situated at the mid-level of the palate, whereas in felids, as well as *Thylacoleo carnifex*, it is located at the far caudal end of the palate.

- **Finarelli and Goswami's method**[118]: Two landmarks in the Finarelli and Goswami [118] are situated at bone sutures. By definition, these landmarks are type I[135] and recommendable for data reproducibility. However, their visibility on 3D models of specimens depends greatly on scan resolution of the scan and the quality of mesh renderings. Fossils are also prone to lack visible or easily traceable sutures (see list of specimens affected in Methods), although we agree this problem might be less frequent than having a broken frontal process of the jugal, as argued by Finarelli and Goswami[118].

The landmark taken on the ventral suture of the jugal and the maxilla is problematic in terms of its representativeness for orbital orientation. It is not located on the orbital rim in metatherians, but ventral to it at the level of the molar row. Because our intention is to reconstruct the orbital plane, we would expect relevant landmarks to be placed on the orbital rim, or at least within the orbital fossa. In this case the orbital plane constructed with these landmarks deviates from the intuitive plane one could qualitatively estimate, resulting in diverging estimates of its position (Figure S20, Figure S21, and Table S2). In short, the Finarelli and Goswami method does not render orbital orientation as such, but rather the plane of the rostral part of the zygomatic arch.

- **Pilatti and Astúa's method**[119]: Pilatti and Astúa's landmark "between the two lacrimal foramina of the lacrimal bone" is problematic because the number of lacrimal foramina varies intraspecifically in

extant didelphids[138]. When only one lacrimal foramen is present, as in all specimens of sparassodonts that we studied, we modified the landmark's definition: "between the two lacrimal foramina of the lacrimal bone or at external border of the lacrimal foramen". Although Pilatti and Astúa[119] did not mention this particular variation, they quantified the standard deviation for the 16 didelphid species they studied, giving an evaluation of the potential intraspecies variation in metatherians.

The landmarks "postorbital process of the frontal bone" and "tip of the frontal process of the jugal" are necessarily reduced to a single landmark in all mammals possessing a postorbital bar (e.g., *Thylacosmilus atrox*, *Thylacoleo carnifex*, primates, various felids, herpestids, scandentians, and artiodactyls; e.g.,[117,129,134]). Pilatti and Astúa's method can therefore not be used for these taxa.

Pilatti and Astúa[119] did not find a correlation between verticality and encephalization quotient. However, the plane that they used to calculate verticality is based on the position of the prosthion (most anterior midsagittal point on the alveolar process of the maxilla) and the condyles instead of landmarks directly related to the encephalic cavity. As a result, they in fact tested the correlation between encephalization and the orientation of the orbit to the horizontal cranial base, rather than the encephalic cavity as such.

- **Casares-Hidalgo et al.'s method**[120]: The same comments apply as for Heesy's method regarding the use of type II landmarks and primate anatomical terms (e.g., nasion).

The dorsal plane (for calculation of orbital frontation) is built perpendicular to the sagittal plane and goes through the nasion and inion. Building one plane perpendicular to another transmits the potential error in landmark location for the first plane to the second. In addition, this method of plane construction does not allow estimation of any deformation that might be present in the specimen.

Similar to Pilatti and Astúa's method, the correlation made between encephalization and orbit orientation is based on a plane passing through the basisphenoid and the premaxilla. This plane does not directly represent the orientation of the encephalic cavity, but rather that of the basicranium.

In conclusion, information on vision in mammalian fossils is hard to gather and relies on very few indicia. There are no better or worse methods among the four available in the literature, as they all have advantages as well as disadvantages depending on the research question at hand. Choice of method should be based on which approach best fits the dataset studied and the nature of the problem being investigated. Interpretation of results should then be made in light of the method utilized, as there is no universal standard. In the case of the present study, all four methods were used in order to determine how the unique orbital orientation of *Thylacosmilus atrox* compared to that of other mammals. Our main text focuses on results using Heesy's method[117] because the resulting orbital planes relate to biological significance, measurements of angles are easily acquired, and applicable data are already available for almost all the groups of interest (see main text).

Supplementary Results

All results are given in Tables S3 and S4, organized according to the four methods used to quantify orbital orientation ([117–120], see Methods). As expected, there are large intermethod differences in the values obtained, which stems from the fact that in each method orbital planes are determined using different landmarks (see Discussion of the methods). In consequence, values cannot be directly compared and must be considered separately.

Orbital orientation measured according to Heesy's method[117] is illustrated in Figures S2-S7, Figures S12-19 and Figure 34.

Orbital convergence of *Thylacosmilus* has mean value of 32.8°. Value for FMNH P14531 are 30.0° for the right orbit and 39.6° for the left orbit, whereas MMP 1433-M has narrower values of 27.7 (right orbit) and 33.7° (left orbit). Orbit convergence for non-*Thylacosmilus* sparassodonts ranges between 47.0 and 86.1° with mean value of 59.0°. Mean convergence values are 63.2° for Hathliacynidae and 56.0° for Borhyaenidae.

Thylacosmilus has the smallest orbital convergence recorded among sparassodonts and marsupials. Its orbits are more divergent than the dasyuromorph *Sarcophilus harrisii* (40.9°), the didelphimorph *Marmosa robinsoni* (42.4°; [116]), and the diprotodonts *Thylacoleo carnifex* (mean value of 49.8°) and *Petaurus australis* (42.7°; [116]). Angle values for *Thylacosmilus* are also among the smallest recorded for extant carnivorans (e.g., the euplerid *Galidictis fasciata* [34.2°], the herpestid *Herpestes semitorquatus* [34.3°], the viverrid *Prionodon pardicolor* [38.2°]; [116]), and smaller than the mean value for any of the fossil sabertooth cats (45.3°; *Homotherium serum*, *Smilodon fatalis,* and *Smilodon populator*). When compared to other non-carnivorous placentals, *Thylacosmilus* has an orbital convergence value very similar to some artiodactyls (e.g., *Cervus elaphus*, *Pelea capreolus*, *Procapra gutturosa*, *Redunca redunca*, *Tayassu tajacu*), rodents (*Anomalurus derbianus*, *Anomalurus fraseri*, and *Petaurista petaurista*) and some treeshrews (*Anathana ellioti* and *Tupaia belangeri*)[116].

Non-*Thylacosmilus* sparassodonts have higher degrees of orbital convergence than dasyurids (mean value of 44.1°), with the exception of *Thylacinus cynocephalus* (56.1°, [116]; 63.8°, NMB c.2526). They also have highly convergent orbits when compared to didelphids, but are similar to the mean value for diprotodont marsupials (57.7°; [116]). When compared to other carnivorans, non-*Thylacosmilus* sparassodonts share the highest values found in felids and hyaenids (62.7° and 59.1, respectively; [116]).

Orbital verticality of *Thylacosmilus* ranges between 62.9° and 88.2° with mean value of 79.1°. These values are among the highest registered among

mammals and are characteristic of horses (genus *Equus*, mean value of 78.6°; [116]), artiodactyls (75.0°; [116]), hyracoids (76.0°; [116]), some tupaiids (e.g., *Anathana ellioti*, 79.0° and *Tupaia minor*, 79.7°; [116]), sloths (e.g., *Bradypus variegatus*, 81.5°; [116]), and herpestids (e.g., *Herpestes ichneumon*, 87.8°; [116]).

Orbital verticality for non-*Thylacosmilus* sparassodonts ranges between 30.8 and 62.2° with mean value of 47.3°. Mean verticality is 42.3° for Hathliacynidae and 51.8° for Borhyaenidae. These values are similar to ones recorded for extant didelphids (e.g., 43.5°, *Didelphis virginiana*; 48.9°, *Chironectes minimus*) but smaller than dasyuromorphs (54.1°). *Thylacoleo carnifex* has a high orbit verticality (61.2°) in comparison to extant diprotodonts (49.1°; [116]), but not as high as *Thylacosmilus* and extant carnivorans. Among carnivorans, the sabertooth felids have values congruent with those for extant felids (*Smilodon populator*: 63.9°; *Homotherium serum*: 56.3°). The lowest values for felids are registered by *Panthera leo* (47.5°; 50.2° [116]), similar to the mean values for borhyaenids.

When orbital convergence is plotted against orbital verticality (Figure S2 and Figure S3), non-*Thylacosmilus* sparassodonts are clustered together with extant marsupials and *Thylaceoleo carnifex*, not far from fossil sabertooths. In fact, *Homotherium serum* is very near to *Pharsophorus* sp. By contrast, *Thylacosmilus* plots far away from all metatherians (nearest is the dasyurid *Sarcophilus harrisii*) and carnivorans (nearest is the euplerid *Galidictis fasciata*), and groups instead with the bovid *Redunca redunca* and the tupaiid *Anathana ellioti*.

Orbital frontation of *Thylacosmilus* ranges between 55.2° and 85.8°. With a mean value of 79.2°, the orbits of FMNH P-14531 are much more frontated than those of MMP 1433-M (mean value of 62.5°). These values are the highest registered for any marsupials, higher than *Thylacinus cynocephalus* (55.2 °; 60.7 [116]) and *Thylacoleo carnifex* (54.9°). *Thylacosmilus* also has among the highest frontation values registered for placentals, competing only with some artiodactyls (e.g., *Redunda*, *redunca*: 83.0°, *Cervus elaphus*: 69.4°; [116]), perissodactyls (e.g., *Equus zebra*: 66.6°; [116]), and the meerkat (*Suricata suricatta*: 72.0°; [116]). The high

orbital frontation of *Thylacosmilus* also stands out when compared to sabertooth felids (*Smilodon fatalis*, 37.8° and *Smilodon populator*, 43.3°).

Orbital frontation for non-*Thylacosmilus* sparassodonts ranges between 13.4 and 43.2° with mean value of 23.5°. Mean frontation is 19.4° for Hathliacynidae and 30.3° for Borhyaenidae. Because *Arctodictis sinclairi* is similar to other sparassodonts with a mean orbital frontation of 20.2°, the high frontation of borhyaenids is only represented by *Borhyaena tuberata* MPM-PV 3625 (mean value of 40.5°), similar to the mean value for extant diprotodonts (38.7°; [116]). Hathliacynids and *Arctodictis sinclairi* have lower orbital frontation than extant didelphids (34.7°; [116]) but are very similar to *Dasyurus hallucatus* and *Dasyurus maculatus* (22.3° and 25.7°, respectively). The other dasyurid *Sarcophilus harrisii* stands out for its higher orbit frontation (61.4°), similar to *Thylacinus cynocephalus* (50.0°; 60.7° [116]).

When orbital convergence is plotted against orbital frontation (Figure S4 and Figure S5), *Thylacosmilus* is situated at a notable distance from all other sparassodonts and carnivorans. Nearest to it are artiodactyls (*Cervus elaphus*, *Ourebia ourebi*, *Procapra gutturosa*, *Tayassu pecari*) and the tupaid *Anathana ellioti*. On the contrary, non-*Thylacosmilus* sparassodonts are grouped together near other marsupials. *Borhyaena tuberata*, positioned near the viverrid *Genetta angolensis*, is the most distant of non-*Thylacosmilus* sparassodonts. *Thylacoleo carnifex* is isolated with the tree shrew *Ptilocercus lowii*. Carnivorans clades are not well defined, although some families are more diffuse than others (e.g., felids at upper right corner, herpestids at lower right corner and viverrids at the center). However, *Smilodon gracilis* and *Smilodon fatalis* are not clustered together within the felid cloud but are instead closer to herpestids, viverrids, euplerids and some canids (*Speothos venaticus* and *Vulpes vulpes*).

Orbitotemporal angle of *Thylacosmilus* is 121.2° (left) and 135.3° (right) for FMNH P-14531 and 141.0° for the left side of MMP 1433-M. Mean orbitotemporal angle for non-*Thylacosmilus* sparassodonts is 153.4° and ranges

between 139.6-169.7°. Mean orbitotemporal angle is 155.1° for Hathliacynidae and 150.3° for Borhyaenidae, although *Arctodictis sinclairi* has higher mean values (160.4) than *Borhyaena tuberata* (140.3°).

Thylacosmilus has an orbitotemporal angle smaller than those of other sparassodonts, with the exception of *Borhyaena tuberata*. MPM-PV 3645 *Thylacoleo carnifex* (140.3°) is close to *Thylacosmilus*, but didelphids (151.2° [116]; e.g., *Didelphis virginiana*, 151.2° and *Chironectes minimus*, 151.8°), diprotodonts (145.2° [116]), and species of *Dasyurus* (*Dasyurus hallucatus*, 156.3°; *Dasyurus maculatus*, 155.6°) are all larger. Interestingly, *Sarcophilus harrisii* (127.4°) and *Thylacinus cynocephalus* (123.7° [this study]; 125.6° [116]) share lower values of the orbitotemporal angle than do *Thylacosmilus*, *Borhyaena tuberata*, and *Thylacoleo carnifex*. In fact, *Sarcophilus harrisii* and *Thylacinus cynocephalus* have orbitotemporal angles similar to extant felids (125.5°; calculated from [116]), whereas *Smilodon populator* and *Smilodon fatalis* have higher orbitotemporal angles (135.4° and 148.4°, respectively), similar to *Thylacosmilus*, *Borhyaena tuberata*, and *Thylacoleo carnifex*.

When orbital convergence is plotted against orbitotemporal angle (Figure S6 and Figure S7), *Thylacosmilus* is isolated from all other taxa. Non-*Thylacosmilus* sparassodonts are aligned near other marsupials, in particular the phalangerids *Trichosurus vulpecula* and *Phalanger vestitus*, with the exception of *Borhyaena tuberata* which is surrounded by *Dactylopsila palpator*, *Procyon lotor*, *Panthera leo*, *Genetta angolensis*, and *Crocuta crocuta*. *Thylacoleo carnifex* is surrounded by canids but near the grouping of marsupials. *Sarcophilus harrisii* is isolated from all other taxa with the exception of *Suricata suricatta* and *Petaurus australis*. *Thylacinus cynocephalus* stands in the vicinity of felids, away from any other marsupials. Interestingly, the sabertooth felids are not near the felids, but situated among the herpestids. When compared to non-carnivorous placentals, *Thylacosmilus* is similar to extant artiodactyls, in particular the red deer (*Cervus elaphus*, Cervidae), the bohor reedbuck (*Redunca redunca*, Bovidae), the greatest mouse-deer (*Tragulus napu*, Tragulidae), and the Chacoan peccary (*Catagonus*

wagneri, Tayassuidae). These values are also similar to those for the red giant flying squirrel (*Petaurista petaurista*, Rodentia, Sciuridae), the Madras tree shrew (*Anathana ellioti*, Scandentia, Tupaiidae) and the northern treeshrew (*Tupaia belangeri*, Scandentia, Tupaiidae).

Orbitolabyrinth angle of *Thylacosmilus* is 87.8° (left) and 89.4° (right) with mean value of 88.6°, slightly less than the mean value for *Smilodon fatalis* (85.0°). *Thylacosmilus* has higher values than the mean orbitolabyrinth angle of *Sipalocyon gracilis* (36.0°), *Dasyurus hallucatus* (59.7°), *Didelphis virginiana* (74.4°), and *Thylacinus cynocephalus* (71.7°).

Orbital orientation measured according to Finarelli and Goswami's method[118] is illustrated in Figure S8, Figures S20-23, and Figure 34.

Orbital convergence of *Thylacosmilus* has a mean value of 16.1° and ranges between 13.1° (left orbit of MMP 1433-M) and 18.9° (left orbit of FMNH P-14531). Values for non-*Thylacosmilus* sparassodonts range between 15.0-28.6° with mean value of 24.0°. Mean convergence values are 24.4° for Hathliacynidae and 22.1° for Borhyaenidae.

Thylacosmilus has the least convergent orbit among the marsupials included in the present study. It is followed by small values for *Thylacinus cynocephalus* (19.4°) and *Didelphis virginiana* (21.9°). *Dasyurus hallucatus* and *Dasyurus maculatus* have orbital convergence values of 26.0° and 25.4°, similar to Hathliacynidae and *Thylacoleo carnifex* (26.1°) but less than *Chironectes minimus* (28.7°). *Sarcophilus harrisii* has the highest orbital convergence among the marsupials sampled (31.1°). *Thylacosmilus* has higher orbital convergence than fossil *Panthera atrox* (12.4°, Finarelli and Goswami, 2009), but both species are smaller than the mean value of sabertooth felids and extant felids (respectively 21.8° and 32.1°, calculated from[118]). The orbital convergence of *Thylacosmilus* is slightly lower, but similar to the extant carnivorans *Canis lupus*, *Cerdocyon thous*,

Melursus ursinus and the fossil carnivorans *Carpocyon webbi*, *Osbornodon fricki*, and *Arctodus simus*).

Orbital frontation of *Thylacosmilus* is 83.7° for FMNH P-14531 and 79.1° for MMP 1433-M. The mean value of orbit frontation calculated for *Thylacosmilus atrox* (81.4°) is higher than the values for *Thylacinus cynocephalus*. (78.8°), *Sarcophilus harrisii* (71.8°), *Dasyurus maculatus* (66.8°), and *Dasyurus hallucatus* (64.7°). However, it is similar to the sabertooth nimravid (*Barbourofelis morrisi*: 86.6°; [118]) and the felids (*Panthera atrox*: 78.7° [118]; *Smilodon populator*: 77.5°; and *Smilodon fatalis*: 78.7° [this study], 76.6° [118]).

Orbital frontation in non-*Thylacosmilus* sparassodonts ranges between 62.4 and 82.1°. Their mean value is 71.4°, which is higher than the mean frontation value for the didelphids studied (*Didelphis virginiana*, 68.9° and *Chironectes minimus*, 62.0°), but within the range for *Thylacoleo carnifex* (73.5°) and dasyuromorphs. Mean orbital frontation is 76.4° for Hathliacynidae and 70.0° for Borhyaenidae.

When orbital convergence is plotted against orbital frontation (Figure S8), no grouping of clades can be detected. We note in particular the diffuse distribution of canids across the graph. *Thylacosmilus* is isolated from other sparassodonts. *Thylacosmilus* is not far from the extant canids *Cerdocyon thous* and *Otocyon megalotis*, the fossil canids *Aenocyon dirus*, *Osbornodon fricki* and *Carpocyon webbi*, as well as the fossil ursid *Arctodis simus*. *Thylacinus cynocephalus* is also near *Thylacosmilus*, as well as the two sabertooth felids *Smilodon populator* and *Smilodon fatalis*. The three other dasyuromorphs are near non-*Thylacosmilus* sparassodonts, *Didelphis virginiana* and *Chironectes minimus. Thylacoleo carnifex* is isolated further from *Thylacosmilus* and *Borhyaena tuberata*, in the direction of other marsupials.

Orbitolabyrinth angle of *Thylacosmilus* is 80.3° (left) and 87.4° (right) with mean value of 83.9°, which is almost equal to *Smilodon fatalis* (84.2°), *Thylacinus cynocephalus* (86.1°) and *Didelphis virginiana* (86.2°). The non-*Thylacosmilus*

sparassodont *Sipalocyon gracilis* has the lowest orbitolabyrinth value (26.6°), followed by *Dasyurus hallucatus* (69.2°).

Orbital orientation measured according to Pilatti and Astúa's method [119] is illustrated in Figure S9, Figures S24-27.

Orbital convergence of *Thylacosmilus* cannot be measured by this method because of the presence of the postorbital bar (same inapplicability for *Thylacoleo carnifex*; postorbital process of the frontal bone = tip of the frontal process of the jugal). Orbital convergence for non-*Thylacosmilus* sparassodonts ranges between 43.4-82.7° with mean value of 60.1°; 63.1° for Hathliacynidae and 57.8° for Borhyaenidae. These values are slightly higher than the ones for extant marsupials and fossil felids ([119]; this study). On the contrary, *Panthera leo* has the highest orbital convergence measured in our sample (mean values of 71.8°), particularly in comparison to the three fossil felids (41.5°). Orbital convergence for our specimen of *Didelphis virginiana* is 56.1°, similar to the value of *Didelphis albiventris*. Values for *Chironectes minimus* are also congruent with the ones measured by Pilatti and Astúa [119]. Interestingly, convergence as measured in our sample of dasyuromophs (mean value of 46.6°) is smaller than any values recorded for didelphids (mean value of 55.4°, [119]).

Orbital verticality of *Thylacosmilus* and *Thylacoleo carnifex* cannot be measured with this method because of the presence of the postorbital bar. Orbital verticality for non-*Thylacosmilus* sparassodonts ranges between 29.3° and 54.9° with mean value of 40.8°, similar to those for extant didelphids (values ranging between 28.7-65.0° with mean value of 43.1°; [119]). However, mean verticality is 37.9° for Hathliacynidae, less than the values for didelphids (e.g., 39.7° *Didelphis virginiana*; 42.3°, *Chironectes minimus*). Our specimen of *Chironectes minimus* has smaller values than the mean value for specimens of *Chironectes minimus*, but falls within the standard deviation for the species (41° ± 3.8°; [119]). Mean verticality value for borhyaenids is based on the single specimen of *Borhyaena*

tuberata (MPM-PV 3625; 50.8°), but might indicate a high orbital verticality for the family, near ranges in the genus *Dasyurus*. In fact, *Dasyurus hallucatus* and *Dasyurus maculatus* both have verticality values of 50.2°. The other two dasyuromorphs studied have higher values (*Sarcophilus harrisii*: 61.6° and *Thylacinus cynocephalus*: 57.6°), similar to the highest value registered for didelphids (57.3°, *Caluromysiops irrupta* [119]) and *Smilodon populator* (59.5°). On the contrary, *Panthera leo* has a low orbit verticality (36.8°), resembling the one for hathliacynids.

Orbitolabyrinth angle of the non-*Thylacosmilus* sparassodont *Sipalocyon gracilis* is 38.8°, the smallest recorded for metatherians. As a comparison, the mean value for *Thylacinus cynocephalus* is 44.6°, 72.8° for *Didelphis virginiana*, and 62.4° for *Dasyurus hallucatus*: 62.4°). *Smilodon fatalis* registers the highest orbitolabyrinth angle (82.4°).

Orbital orientation measured according to Casares-Hidalgo et al.'s method[120] is illustrated in Figure S10, Figure S11, Figures S28-34.

Orbital convergence of *Thylacosmilus* ranges between 14.6° (left orbit of MMP 1433-M) and 33.7° (left orbit FMNH P-14531) with mean value of 23.5°. *Thylacosmilus* has the smallest convergence measured among carnivorans, with the exception of the walrus (*Odobenus rosmarus*, 25.9°; [120]). Fossil sabertooth felids have higher orbital convergence than *Thylacosmilus atrox* (*Smilodon populator*: 38.2°; *Smilodon fatalis*: 40.7°, 38.2° [120]; *Homotherium serum*: 44.6°, 37.2° [120])

Orbital convergence for non-*Thylacosmilus* sparassodonts has mean value of 59.7° and ranges between 43.9° and 81.4°. Mean values are 62.8° for Hathliacynidae and 57.6° for Borhyaenidae, which are high in comparison to other terrestrial carnivorans such as red panda (*Ailurus fulgens*, 59.2°; [120]), jaguar (*Panthera onca*; 57.3°; [120]) and masked palm civet (*Paguma larvata*, 57.5°; [120]),

but are within the ranges recorded for phocids. *Didelphis virginiana* and *Chironectes minimus* have orbital convergence values similar to *Panthera leo* (55.3°, our study; 53.3°, [120]) but higher than the ones for *Thylacoleo carnifex* (45.6°), dasyurids (46.8°), and *Thylacinus cynocephalus* (50.2°).

Orbital frontation of *Thylacosmilus* is 88.6° for FMNH P-14531 and 82.5° for MMP 1433-M. These are the highest values recorded among carnivorans, the highest values for which are shared between some felids (e.g., *Acinonyx jubatus*, 72.4° and *Lynx rufus*, 72.5°; [120]), herpestids (e.g., *Herpestes javanicus*, 72.7° and *Suricata suricatta*, 71.2°; [120]), and ursids (*Helarctos malayanus*, 71.6° and *Ursus americanus*, 70.6°; [120]). Sabertooth felids, represented by *Smilodon populator* (65.7°), *Smilodon fatalis* (63.4°; 70.3°, [120]), and *Homotherium serum* (66.9°; [120]) have lower orbit frontation values than *Thylacosmilus*, but occupy the range for non-sabertooth felids (48.2°–72.5°; [120]).

Orbital frontation for non-*Thylacosmilus* sparassodonts ranges between 20.3 and 55.4° with mean value of 37.9°. Mean frontation is 33.7° for Hathliacynidae and 44.2° for Borhyaenidae. Although these values are smaller than the mean value for carnivorans (58.6°; calculated from [120]), they correspond to the ones for didelphids (*Didelphis virginiana*, 43.0°; *Chironectes minimus*, 44.2°). On the contrary, *Dasyurus hallucatus* (47.5°) and *Dasyurus maculatus* (49.1°) have orbits slightly more frontated than in borhyaenids, whereas *Sarcophilus harrisii* (69.1°), *Thylacinus cynocephalus* (61.2°), and *Thylacoleo carnifex* (64.0°) exhibit much higher values.

When orbital frontation is plotted against orbital convergence (Figure S10), *Thylacosmilus* is completely isolated from all other taxa. *Thylacoleo carnifex* is near *Thylacinus cynocephalus* and *Sarcophilus harrisii* and proximate to felids. Sabertooth felids are not aligned with the other felids, but rather with the canids. Didelphids are grouped with the non-*Thylacosmilus* sparassodonts and with the majority of pinnipeds. In fact, two of the pinnipeds (the South American fur seal *Arctocephalus australis* and the crabeater seal *Lobodon carcinophagus*) are not

grouped with non-*Thylacosmilus* sparassodonts, but are placed together with the two *Dasyurus* in our dataset. *Borhyaena tuberata* is positioned slightly apart from other sparassodonts and stands next to *Panthera pardus* and *Panthera leo*.

Orbitotemporal angle of *Thylacosmilus* is 55.5° (left) and 39.0° (right) for FMNH P-14531 and 23.4 for the left side of MMP 1433-M. Mean orbitotemporal angle for non-*Thylacosmilus* sparassodonts is 26.1° and ranges between 14.5 and 40.3°. Mean orbitotemporal angle for Hathliacynidae is 26.9°. *Arctodictis sinclairi* has a low mean value of 23.0°, whereas *Borhyaena tuberata* has the highest mean value, 40.0°. *Thylacosmilus* and *Borhyaena tuberata* have orbitotemporal values similar to *Homotherium serum* (37.4°; [120]), *Smilodon fatalis* (40.2°; 41.1° [120]), and *Smilodon populator* (30.7°). These values are slightly lower than the mean values for felids (46.6°; calculated from [120]), but similar to those for *Panthera leo* (39.4°; 40.0° [120]). Among felids, the serval *Leptailurus serval* has the lowest orbitotemporal angle (20.0°; [120]) but corresponds to the values seen in *Arctodictis sinclairi*. Hathliacynids have orbitotemporal values similar to those for *Dasyurus hallucatus* (23.7°) and *Dasyurus maculatus* (25.7°), although lower than *Didelphis virginiana* (31.4°) and *Chironectes minimus* (30.1°). *Thylacinus cynocephalus* and *Sarcophilus harrisii* have high orbitotemporal angle (48.0° and 54.8°, respectively), corresponding to the values seen in the ursids *Ursus arctos* and *Helarctos malayanus* (50.8° and 52.1°, respectively; [120]), the procyonid *Bassaricyon gabbii* (50.8°; [120]) and felids.

When orbital convergence is plotted against orbitotemporal angle (Figure S11), *Thylacosmilus* is situated far from other taxa. Non-*Thylacosmilus* sparassodonts are grouped together near pinnipeds, with the exception of *Borhyaena tuberata*, which is next to some felids, *Panthera leo* in particular. *Thylacoleo carnifex* stands in the middle of canids with the mustelid *Martes foina*. *Thylacinus cynocephalus* is situated among felids and not far from *Sarcophilus harrisii*, although the latter is outside of the felid cloud. The other two dasyurids are situated apart from canids, felids and sparassodonts.

Orbitolabyrinth angle of *Thylacosmilus* is 84.5° (left) and 90.0° (right) with mean value of 87.2°, similar to the mean value of the sabertooth *Smilodon fatalis* (85.0°). The non-*Thylacosmilus* sparassodonts *Sipalocyon gracilis* has the lowest mean value (40.1°). *Thylacinus cynocephalus* (77.9) and *Didelphis virginiana* (72.8°) have similar values, higher than that of *Dasyurus hallucatus* (59.4°).

Virtual eye reconstruction and ratio. The range in virtual eye diameters reconstructed for the three specimens of *Thylacosmilus* is 33.3–35.3 mm, with mean diameter of 34.0 mm (Figure 1a–d and Figure S35). The mean virtual eye diameter reconstructed for *Thylacoleo* is 19.4 mm. Eye-skull ratio of *Thylacosmilus* ranges between 0.16 and 0.20. With the exception of *Dasyurus hallucatus* (0.21), this ratio is higher than in any extant marsupials (0.09–0.14), and also higher than in *Panthera leo* (0.12–0.14). Except for *Dasyurus hallucatus* again, the eye-rostrum width of *Thylacosmilus* is higher than in any marsupials (1.47–1.56), and much higher than that of *Thylacoleo* (0.67). However, eye-rostrum length ratio for *Thylacosmilus* is only 0.37–0.44, which is within the range of extant marsupials and *Thylacoleo* (0.37).

Cranial proportion. *Thylacosmilus* has a higher rostrum relative length (0.43) in comparison to *Panthera leo* (0.20), *Smilodon populator* (0.24), non-*Thylacosmilus* sparassodonts (0.21–0.30), *Thylacoleo carnifex* (0.27), and marsupials (0.29-0.33), but smaller than *Thylacinus cynocephalus* (0.88). Rostrum proportional width in *Thylacosmilus* is 0.87 for FMNH P14531 and 0.79 for MMP 1433-M and corresponds to the smallest values recorded for sparassodonts (0.92–1.77). *Thylacosmilus* values are within range of the ones for didelphids (0.72, *Didelphis virginiana*; 0.77, *Chironectes minimus*, (40)). *Thylacinus cynocephalus* has the smallest value recorded in this study (0.34) and is much smaller than other dasyuromophs (1.09–1.30). *Thylacoleo carnifex* has a rather high value (1.39), but in the range of non-*Thylacosmilus* sparassodonts. *Panthera leo* (1.56–2.01) and *Smilodon populator* (1.45) have a higher range of rostrum proportional widths than do metatherians.

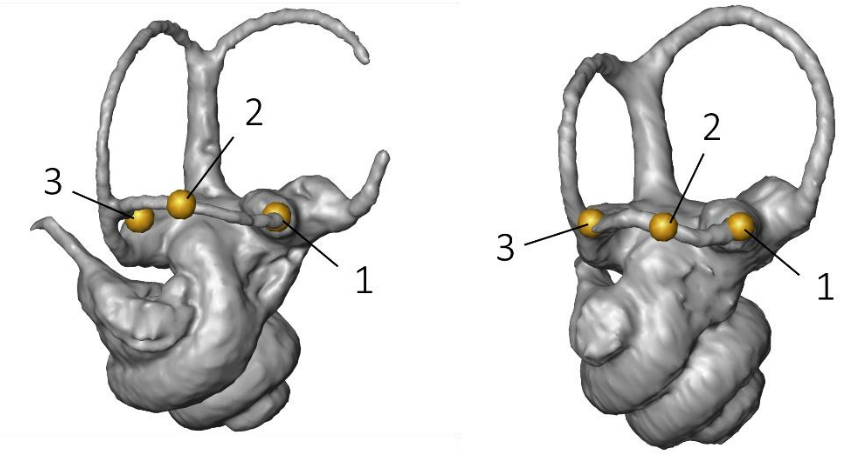

Figure S1. Landmarks on the lateral semicircular canal as seen on *Sipalocyon gracilis* AMNH-VP 9254 (left) and *Didelphis virginiana* TMM M-2517 (right). Annotations for landmarks taken: **1**, at the exit of the canal on the ampulla; **2**, at mid-length of the canal; **3**, at entry of the canal in the pars vestibularis (*Sipalocyon gracilis*) or at the contact of the secondary common crus (*Didelphis virginiana*)

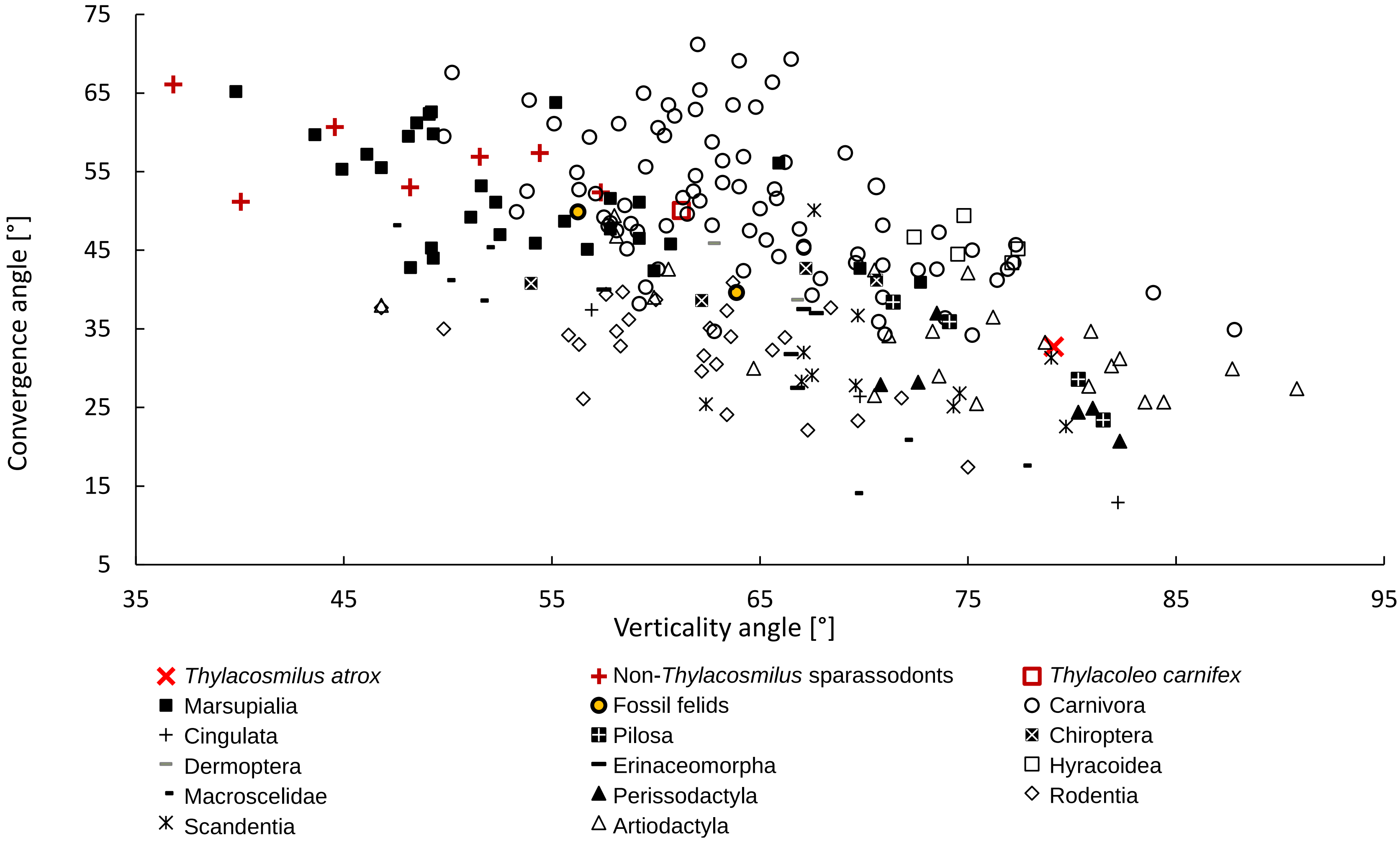

Figure S2. Orbital convergence and verticality relationship in non-primate mammals (modified from Heesy, 2005).

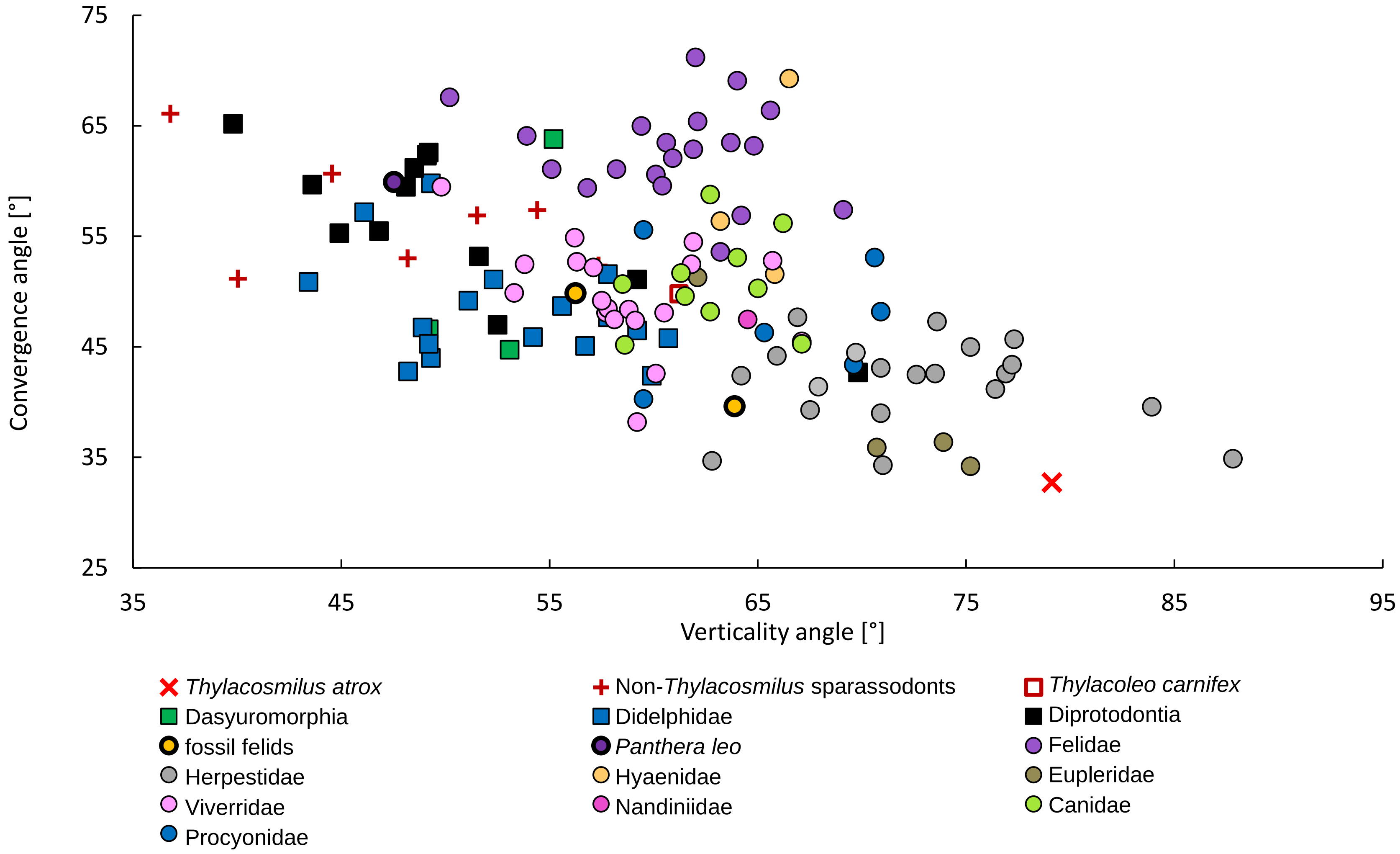

Figure S3. Orbital convergence and verticality relationship in carnivorans and metatherians (modified from Heesy, 2005).

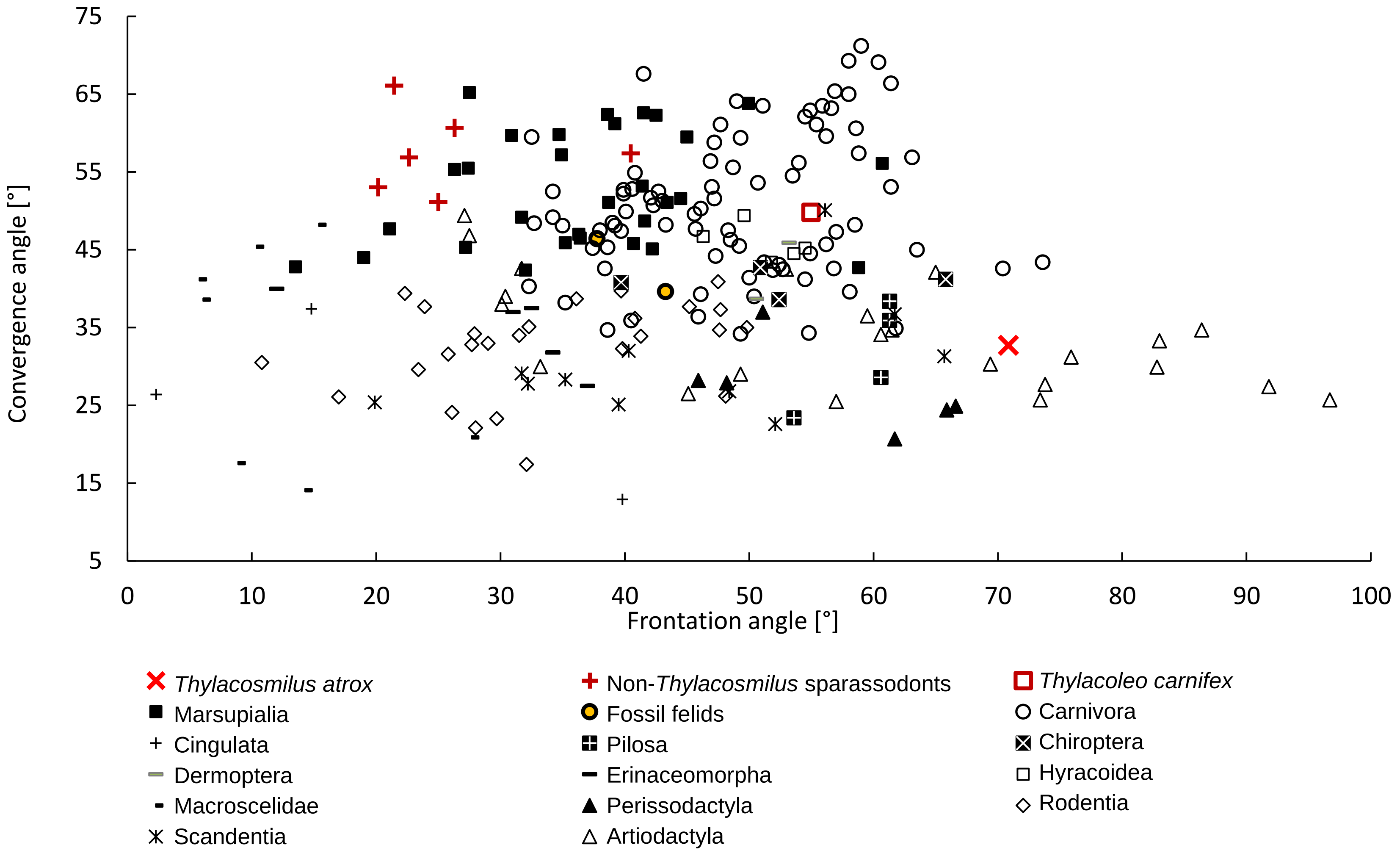

Figure S4. Orbital convergence and frontation relationship in non-primate mammals (modified from Heesy, 2005).

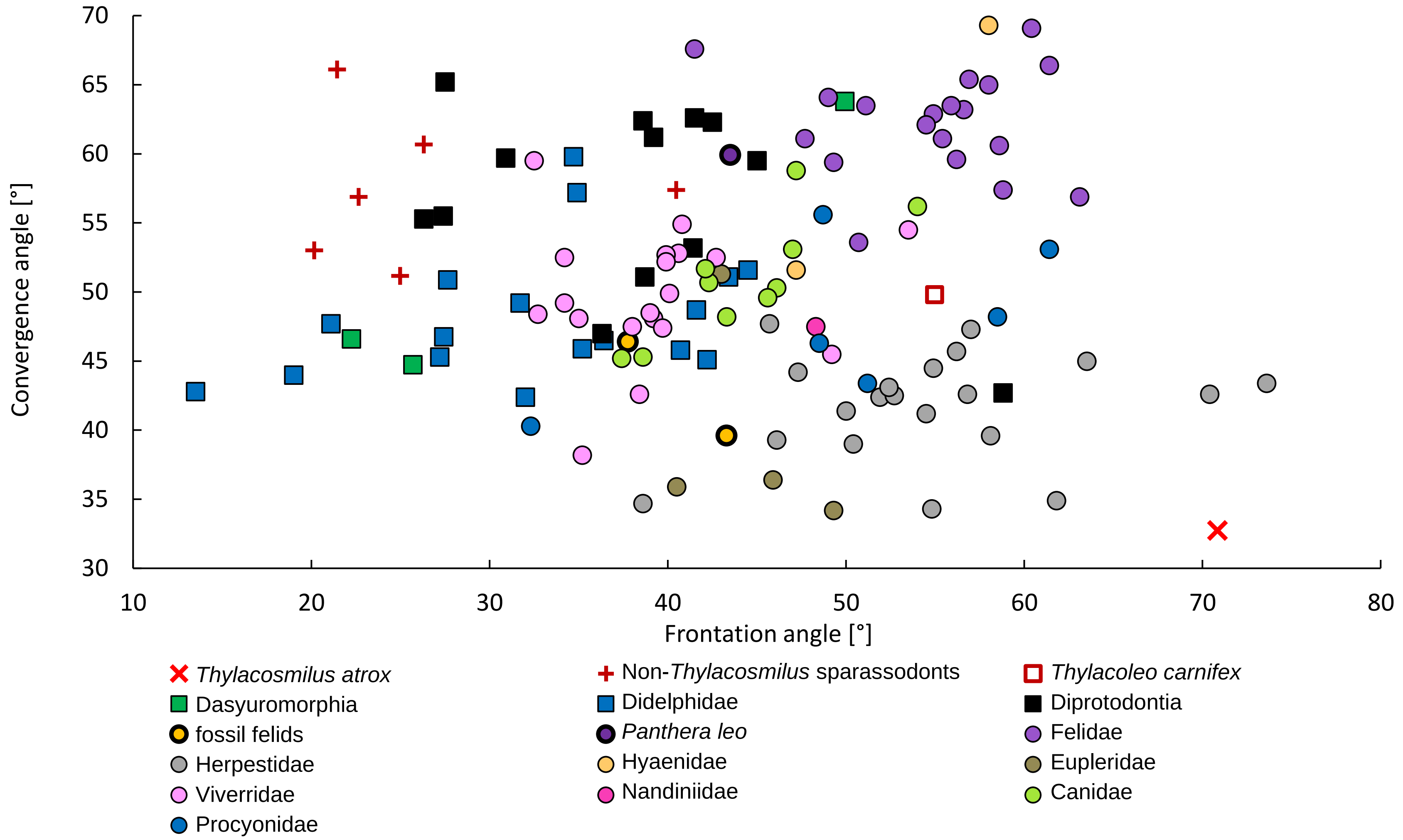

Figure S5. Orbital convergence and frontation relationship in carnivorans and metatherians (modified from Heesy, 2005).

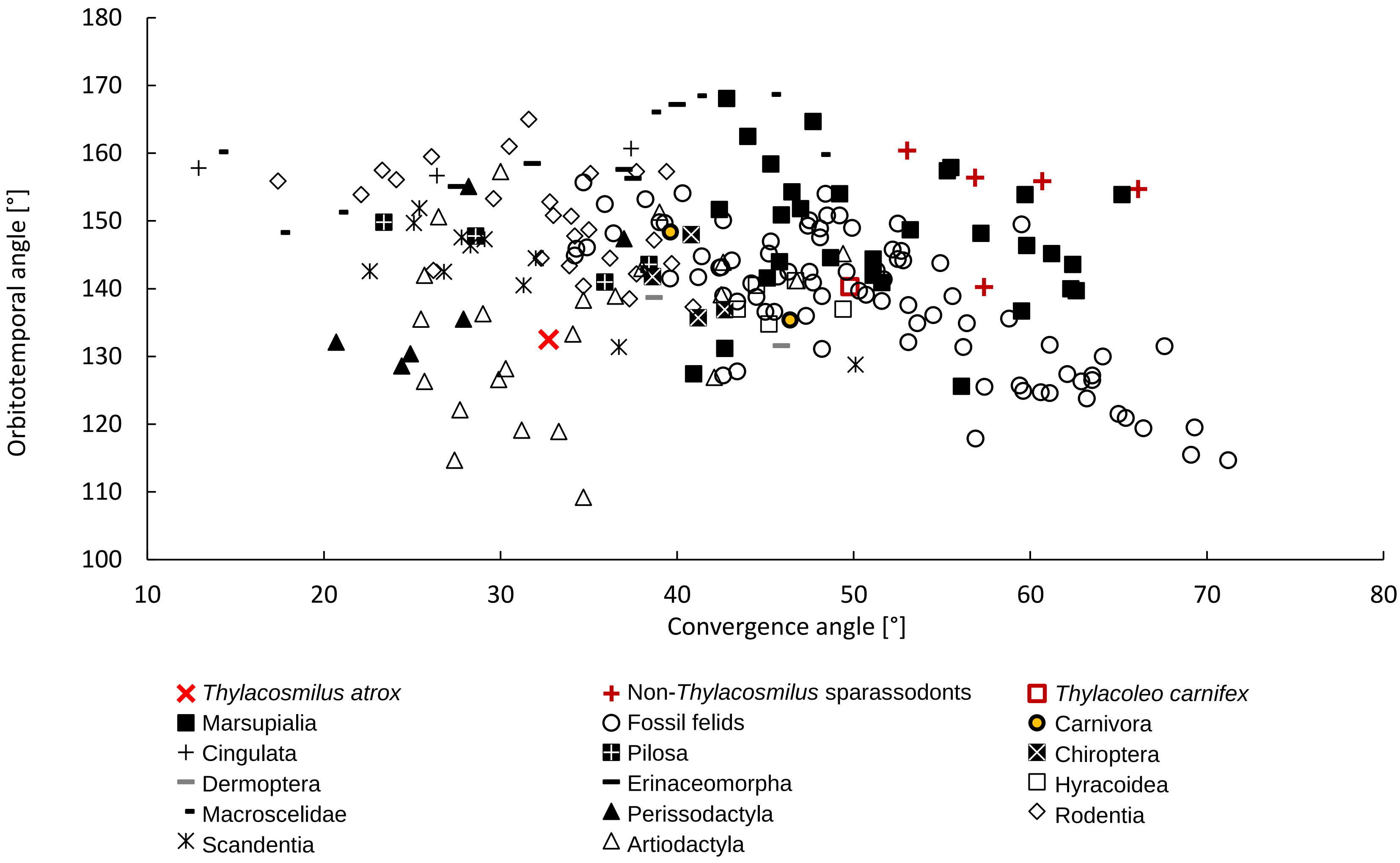

Figure S6. Orbital convergence and orbitotemporal relationship in non-primate mammals (modified from Heesy, 2005).

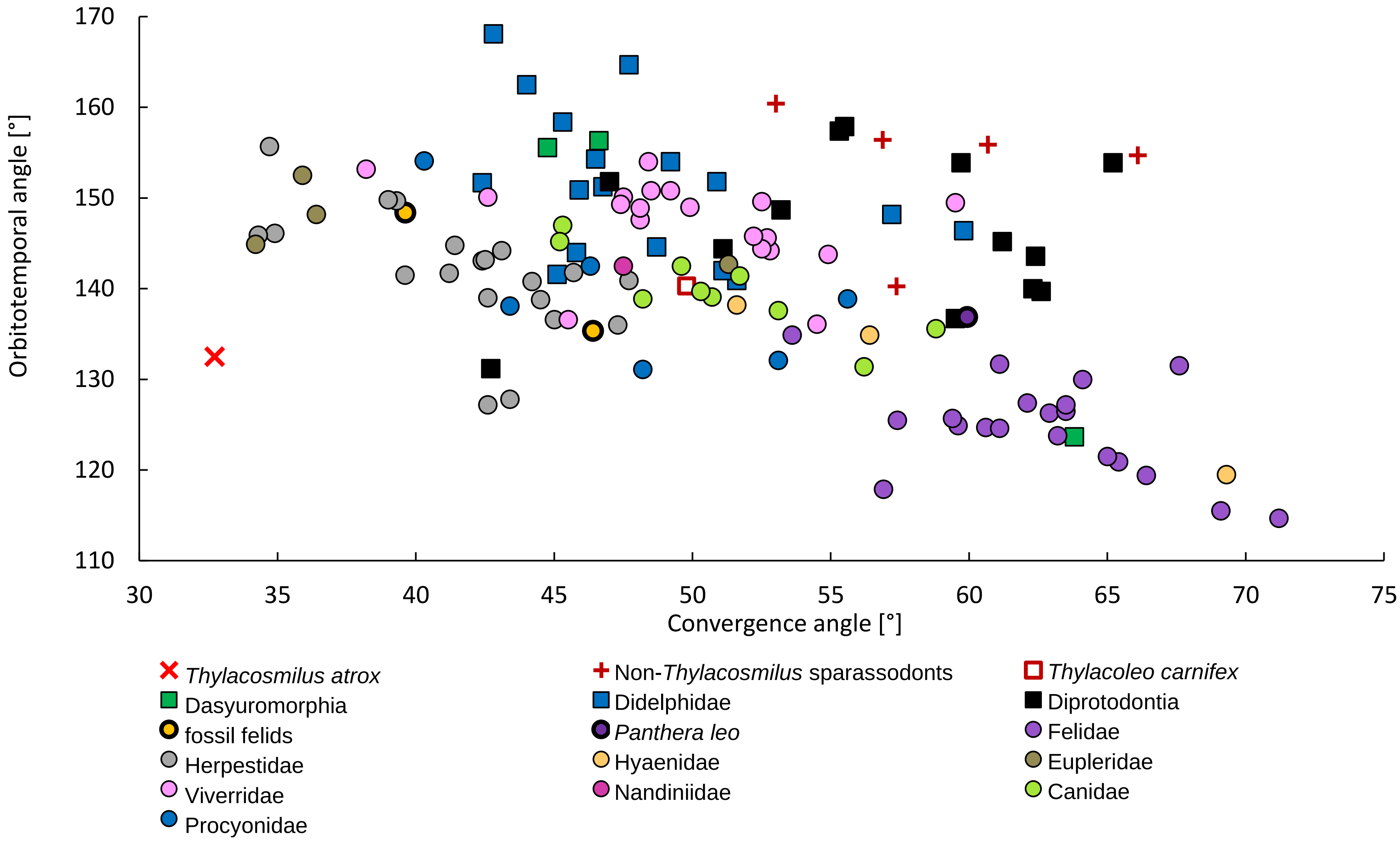

Figure S7. Orbital convergence and orbitotemporal relationship in carnivorans and metatherians (modified from Heesy, 2005).

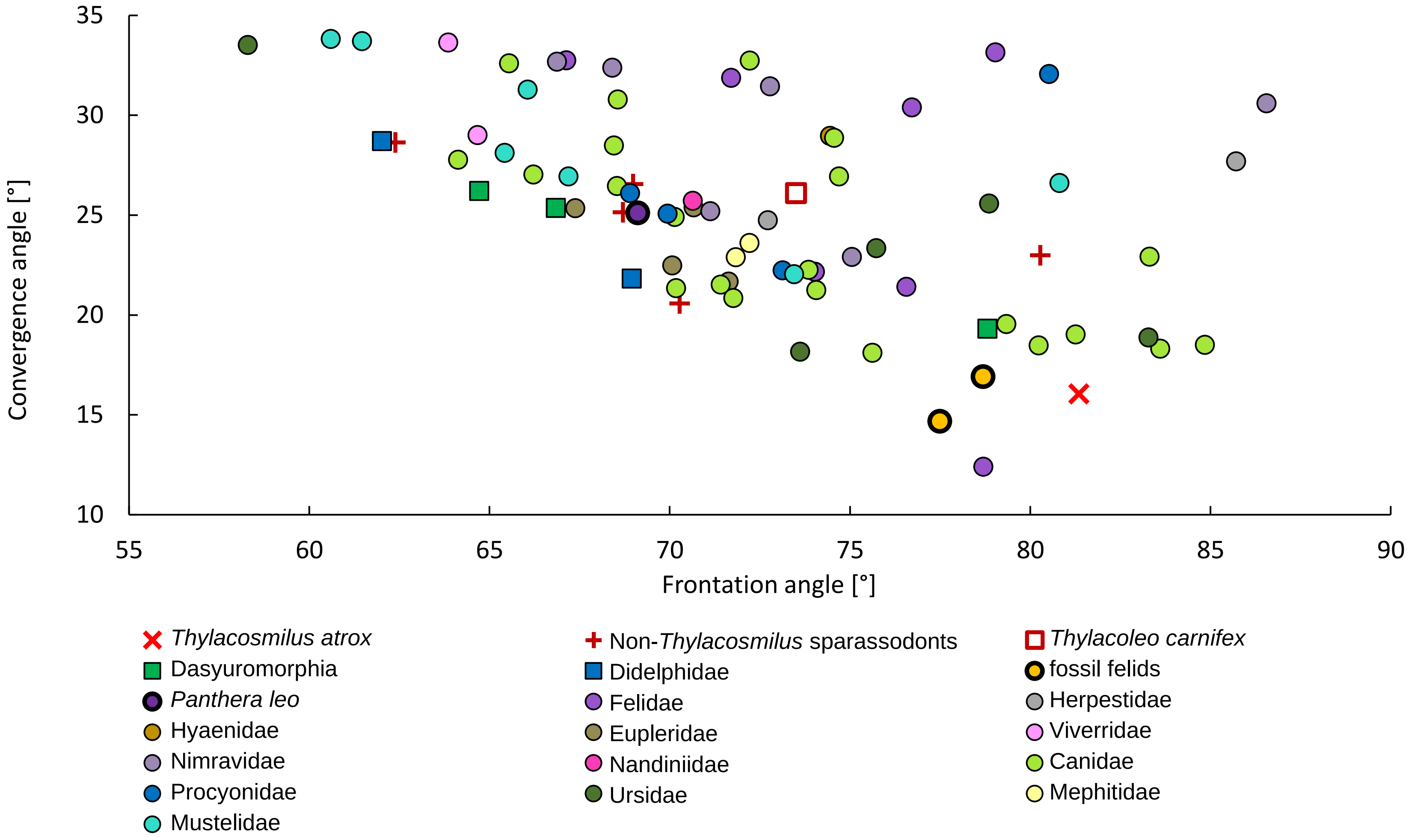

Figure S8. Orbital convergence and frontation relationship in carnivorans and metatherians (carnivoran data from Finarelli and Goswami, 2009).

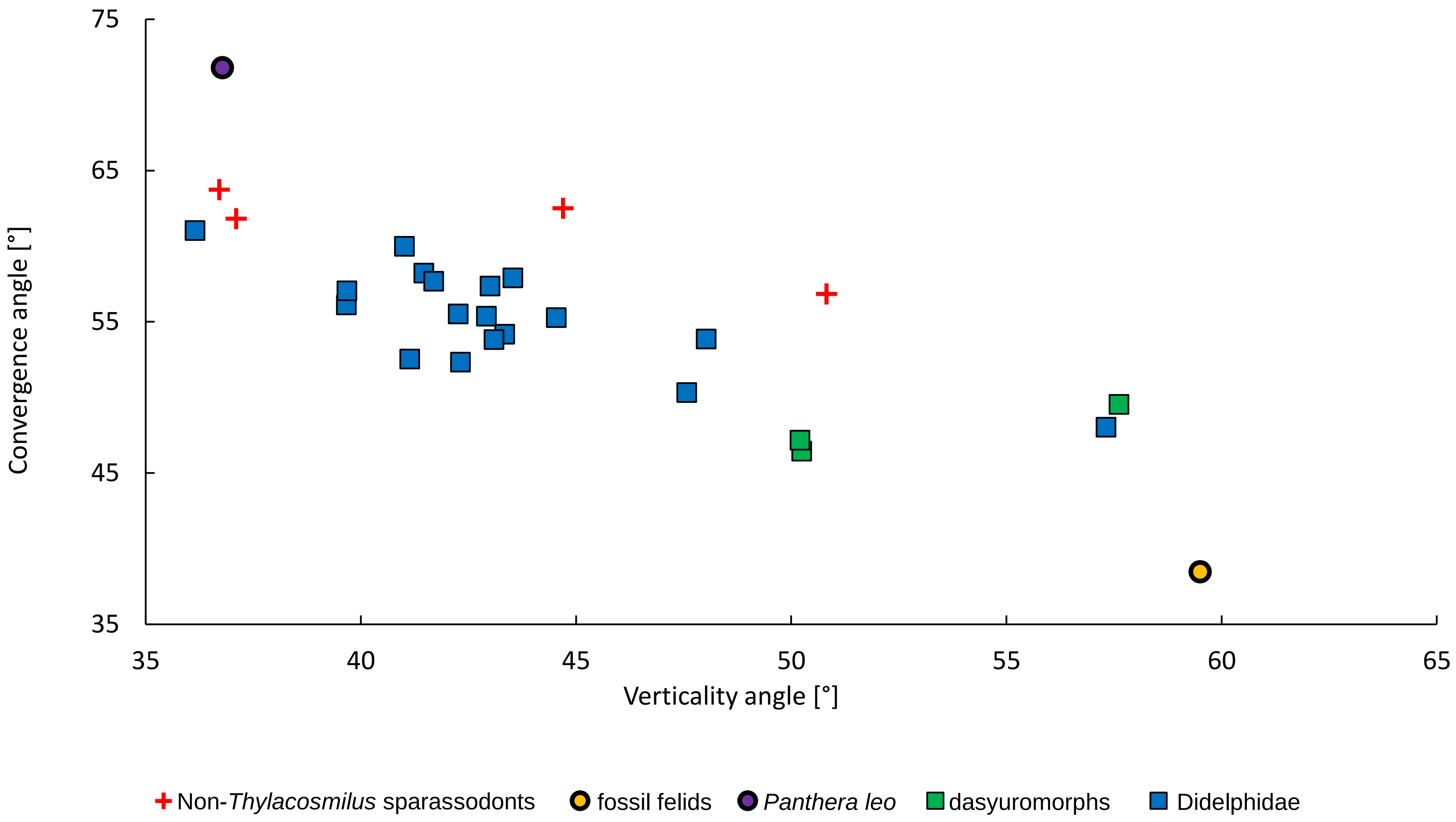

Figure S9. Orbital convergence and frontation relationship in carnivorans and metatherians (didelphid data from Pilattia and Astúa, 2017).

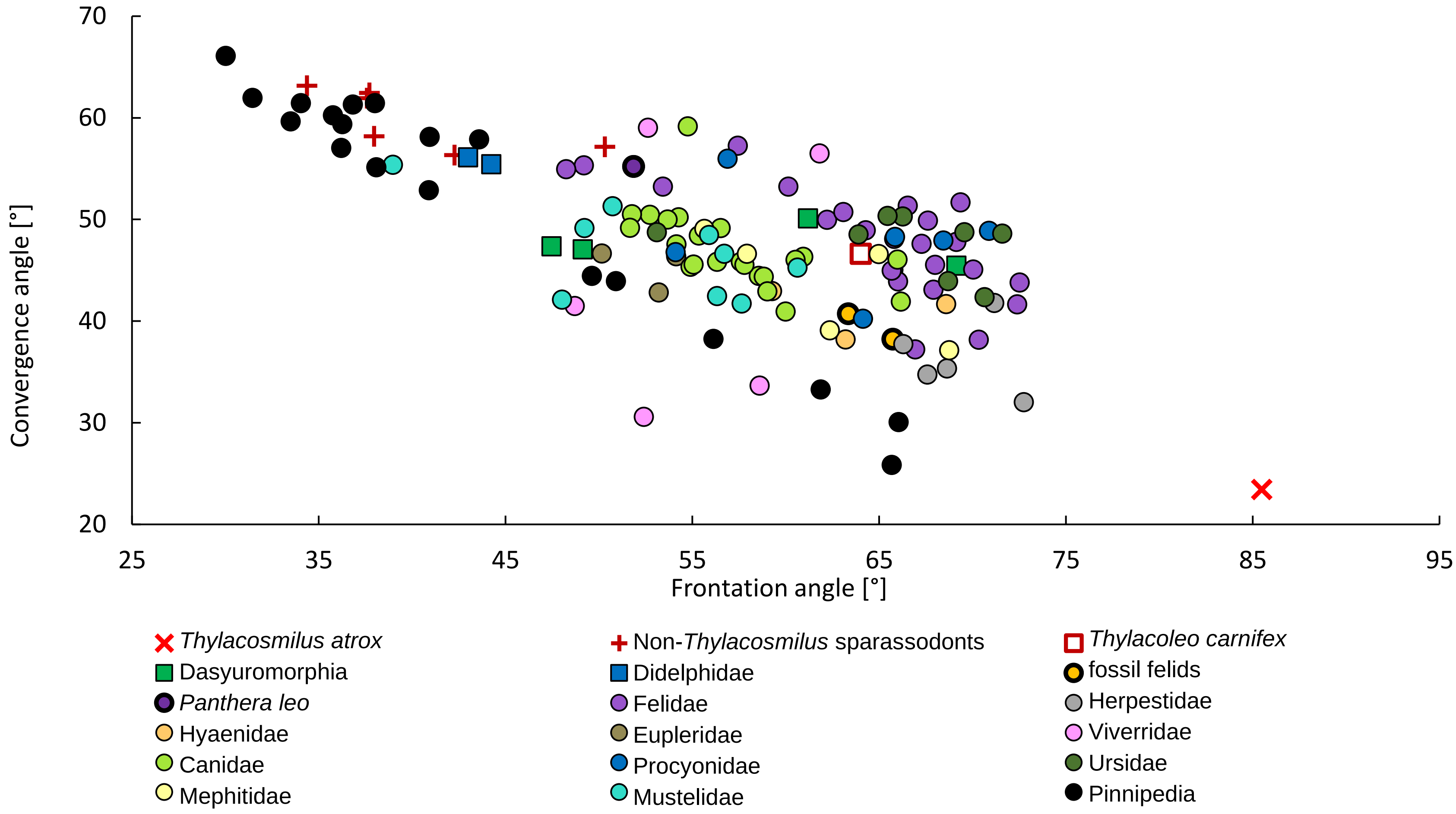

Figure S10. Orbital convergence and frontation relationship in carnivorans and metatherians (carnivoran data from Casares-Hidalgo et al., 2019).

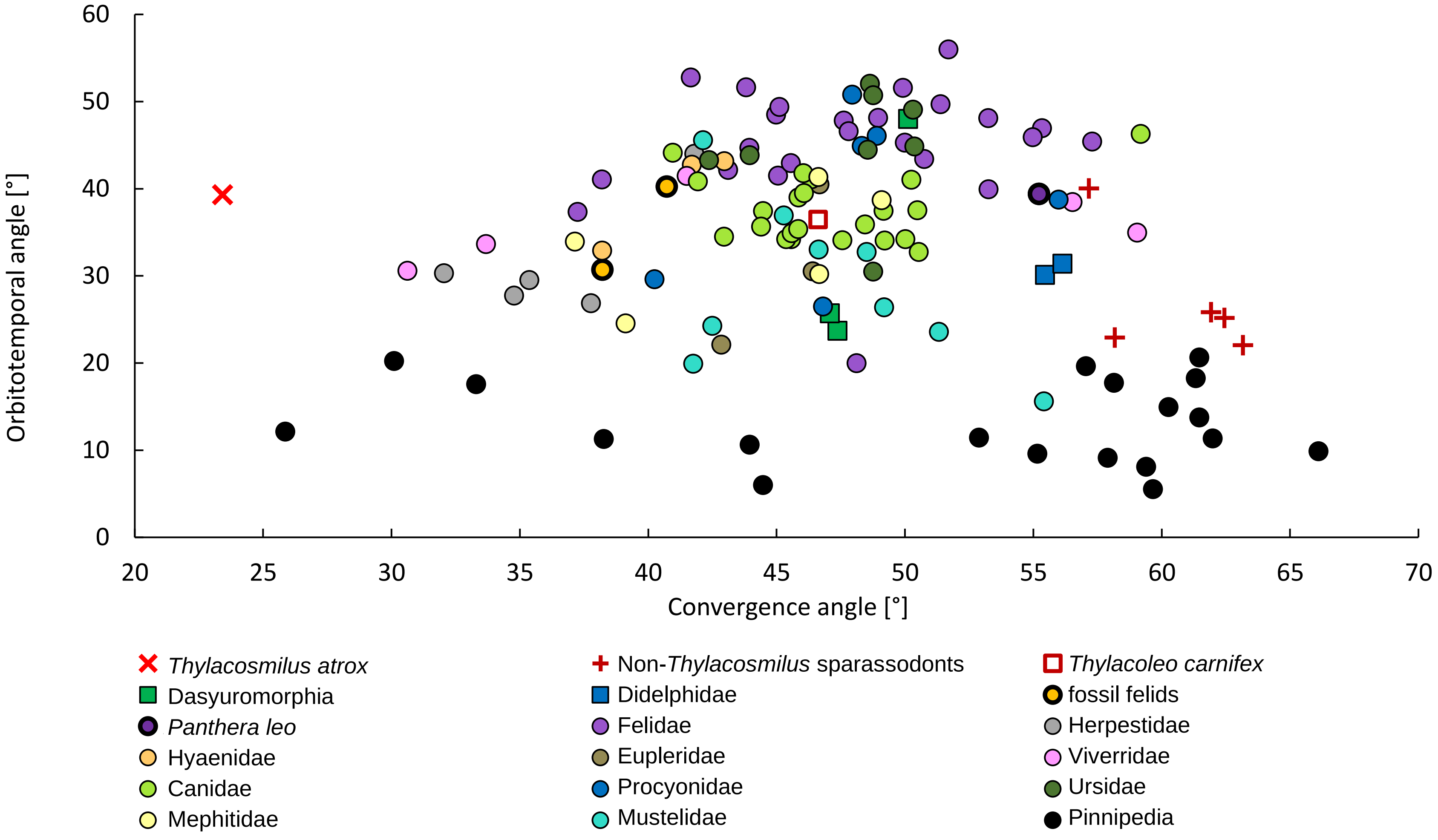

Figure S11. Orbital convergence and orbitotemporal relationship in carnivorans and metatherians (carnivoran data from Casares-Hidalgo et al., 2019).

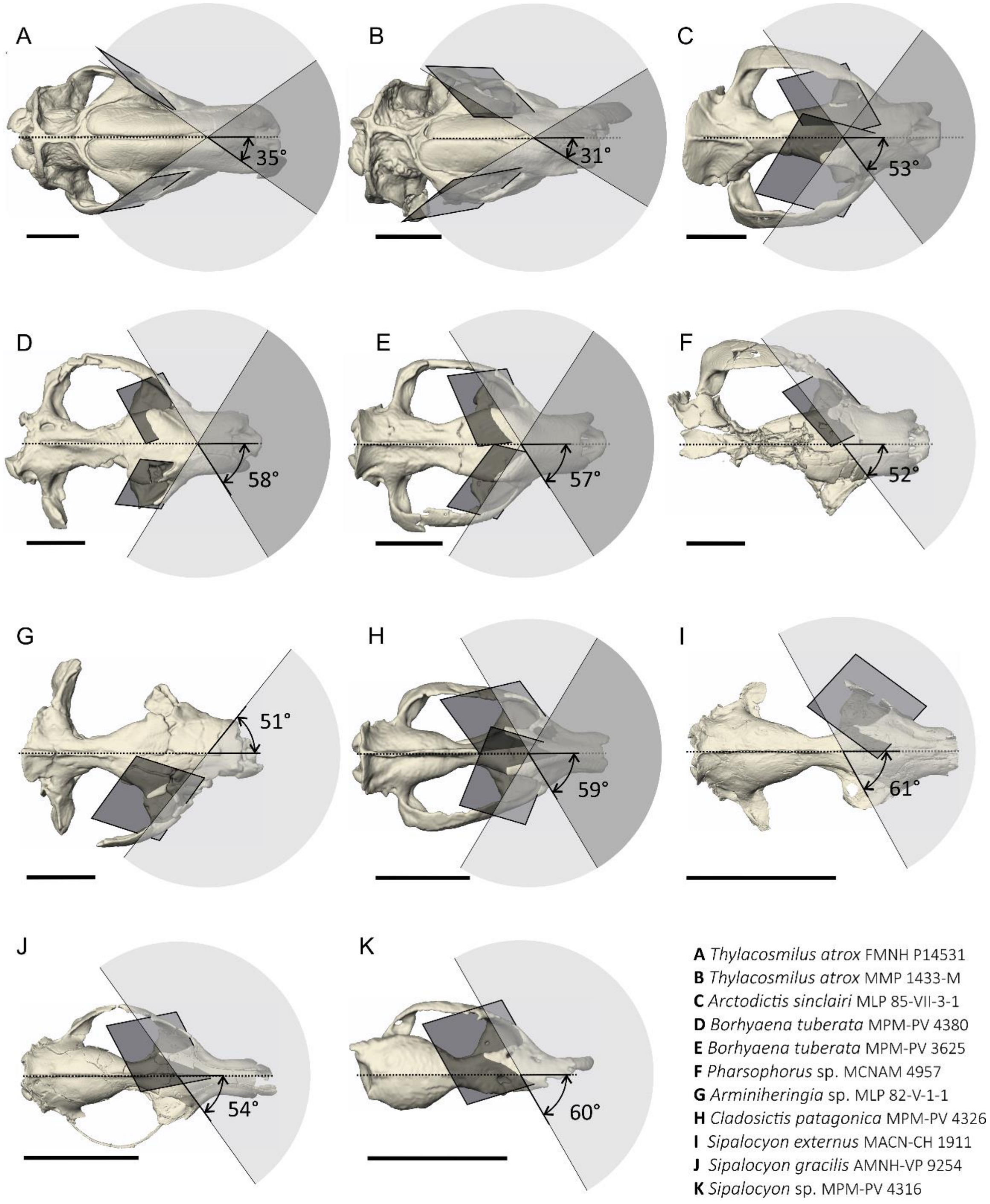

Figure S12. Orbital convergence in sparassodonts, following method of Heesy (2005). Scale bar is 5 cm. Dotted line represents the sagittal plane.

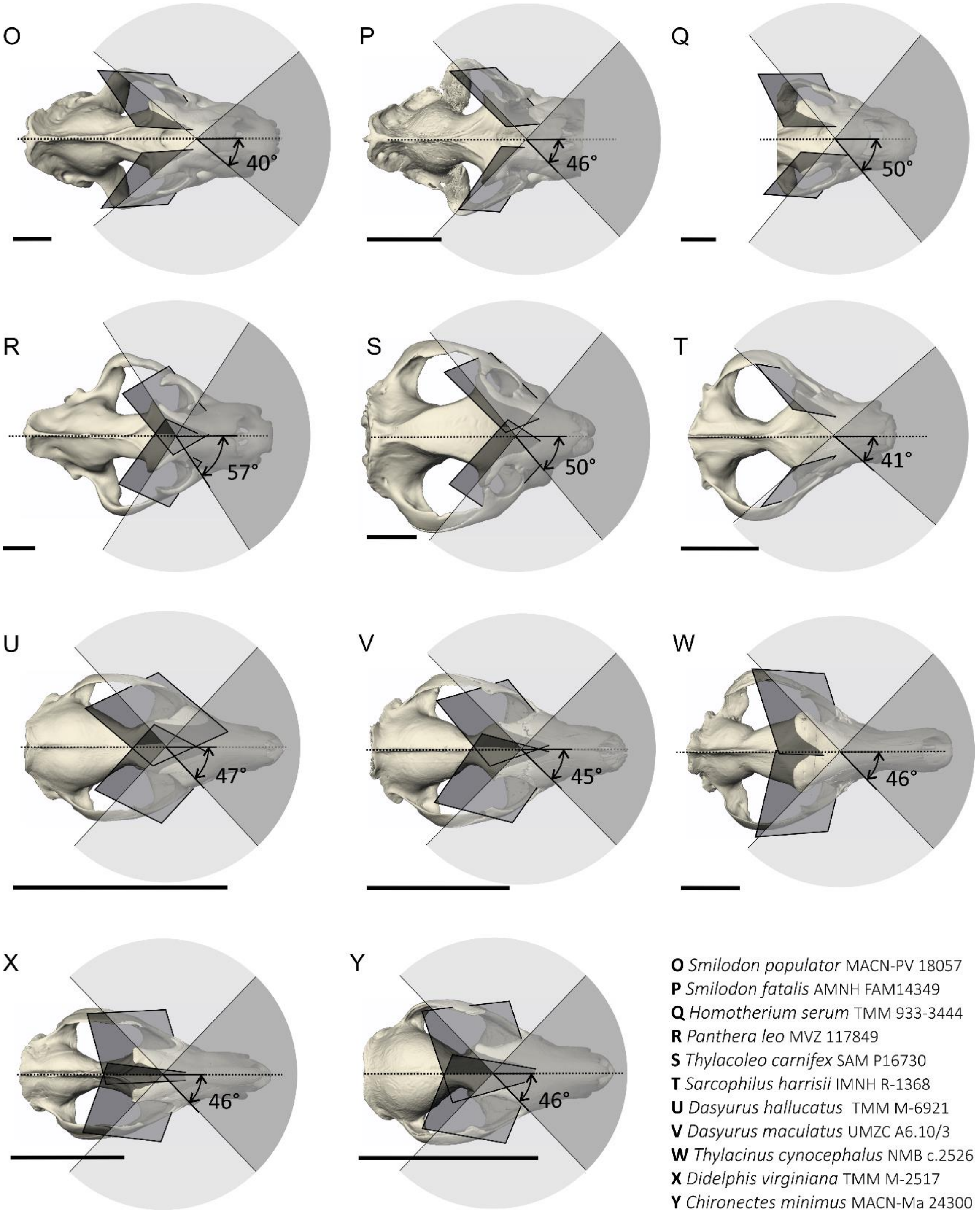

Figure S13. Orbital convergence in comparative sample, following the method of Heesy (2005). Scale bar is 5 cm. Dotted line represents the sagittal plane.

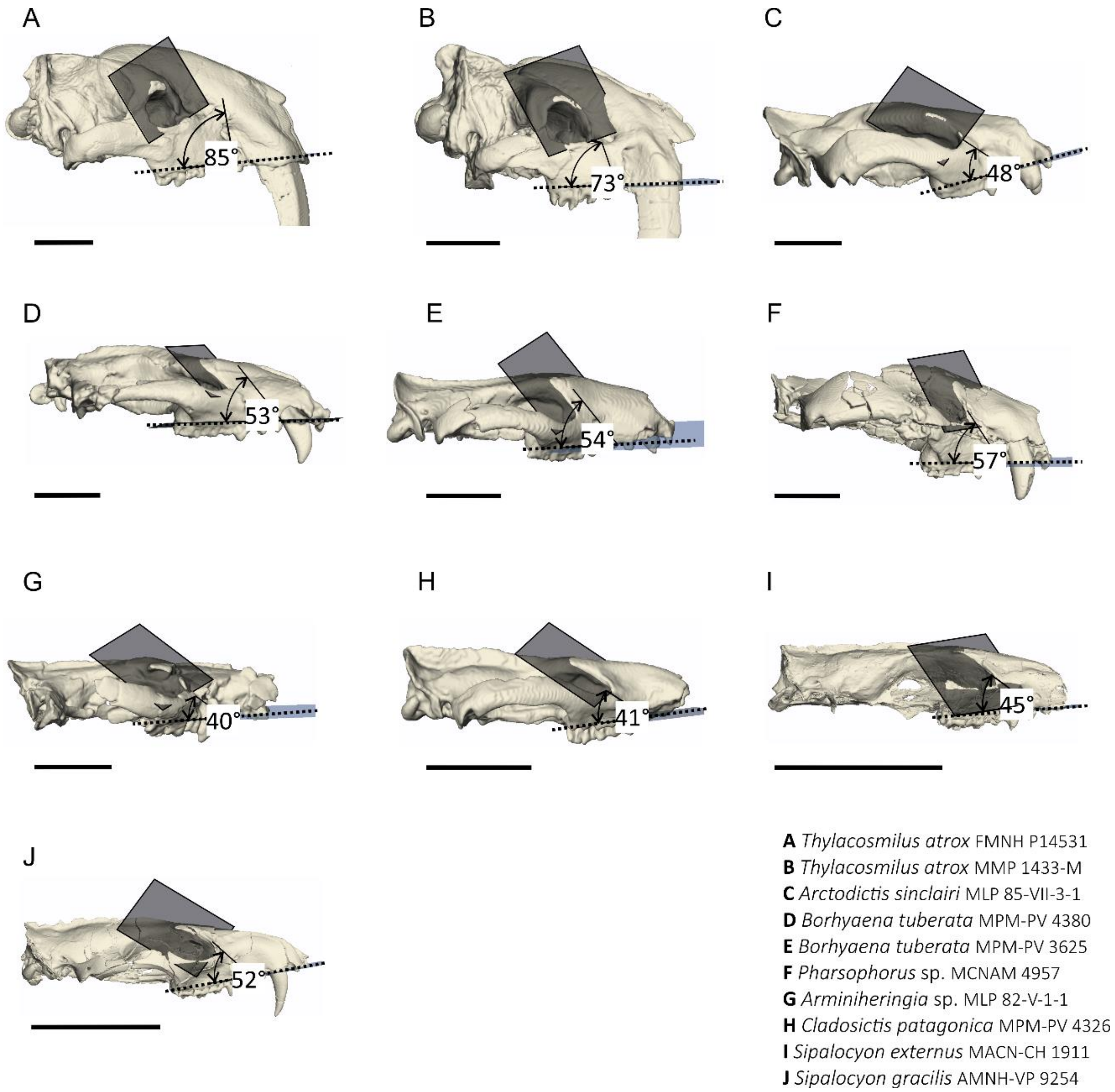

Figure S14. Orbital verticality in sparassodonts, following the method of Heesy (2005). Scale bar is 5 cm. MMP 1433-M, MLP 85-VII-3-1, MPM-PV 4380, MPM-PV 3645, MCNAM 4957, MACN-CH 1911, and AMNH-VP 9254 are left side (reversed). Dotted line represents graphic simplification of palatal plane (visible in blue).

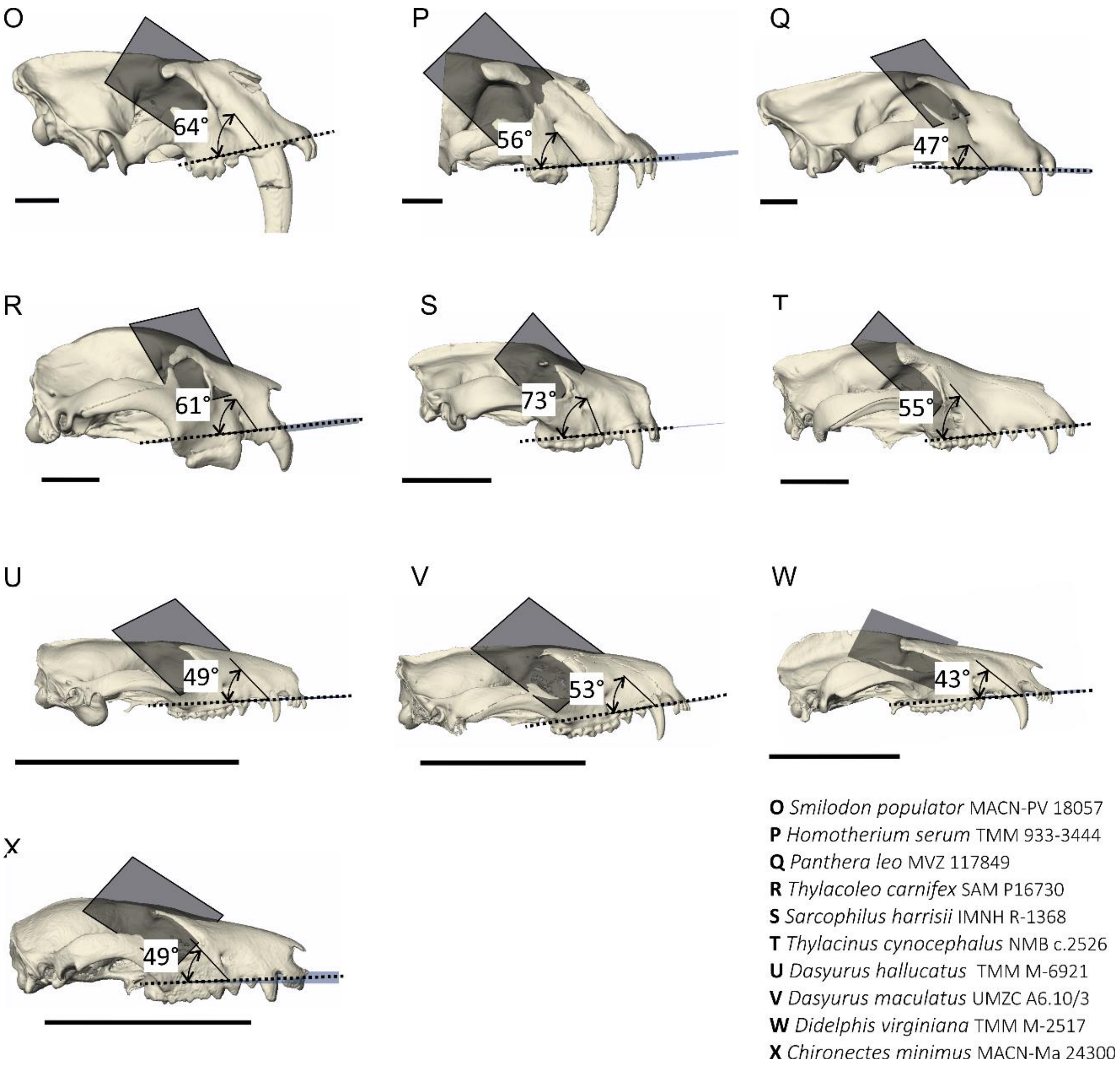

Figure S15. Orbital verticality in comparative sample following method of Heesy (2005). Scale bar is 5 cm. Dotted line represents graphic simplification of palatal plane (visible in blue).

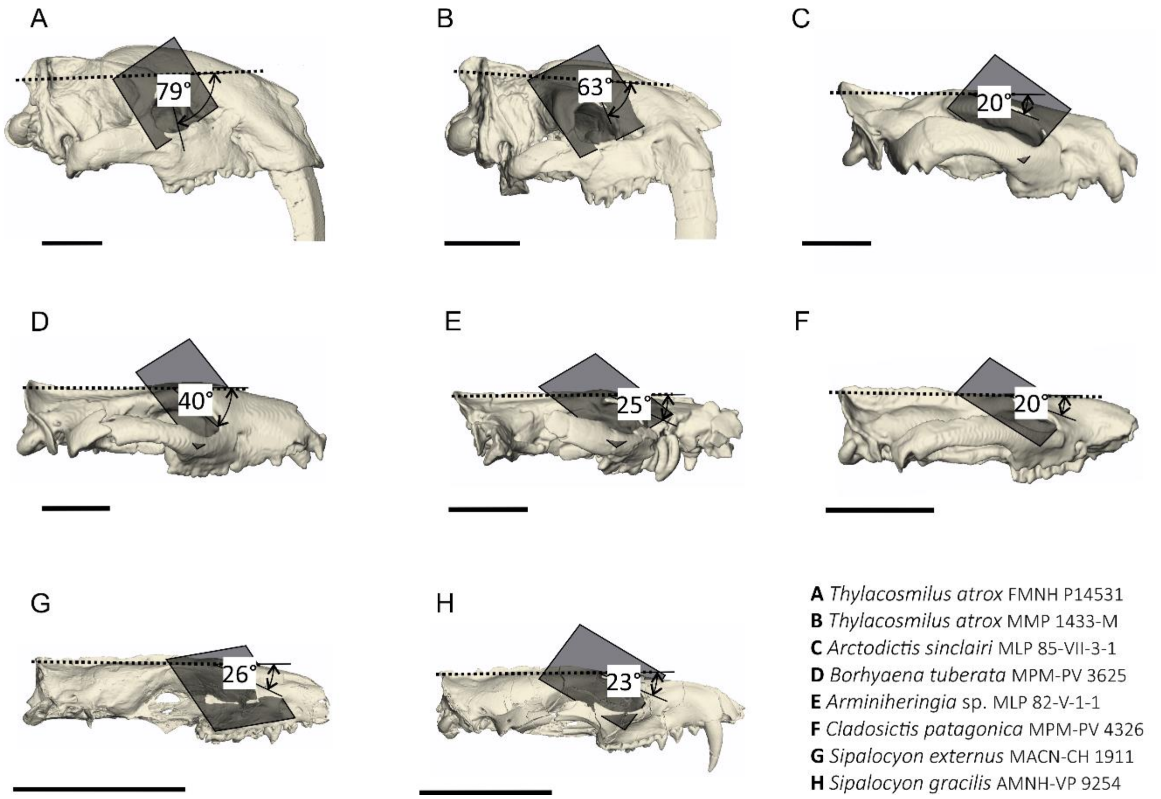

Figure S16. Orbital frontation in sparassodonts, following method of Heesy (2005). Scale bar is 5 cm. MMP 1433-M, MLP 85-VII-3-1, MCNAM 4957, MPM-PV 3645, MACN-CH 1911, and AMNH-VP 9254 are left side (reversed). Dotted line represents graphic simplification of inion-nasion line.

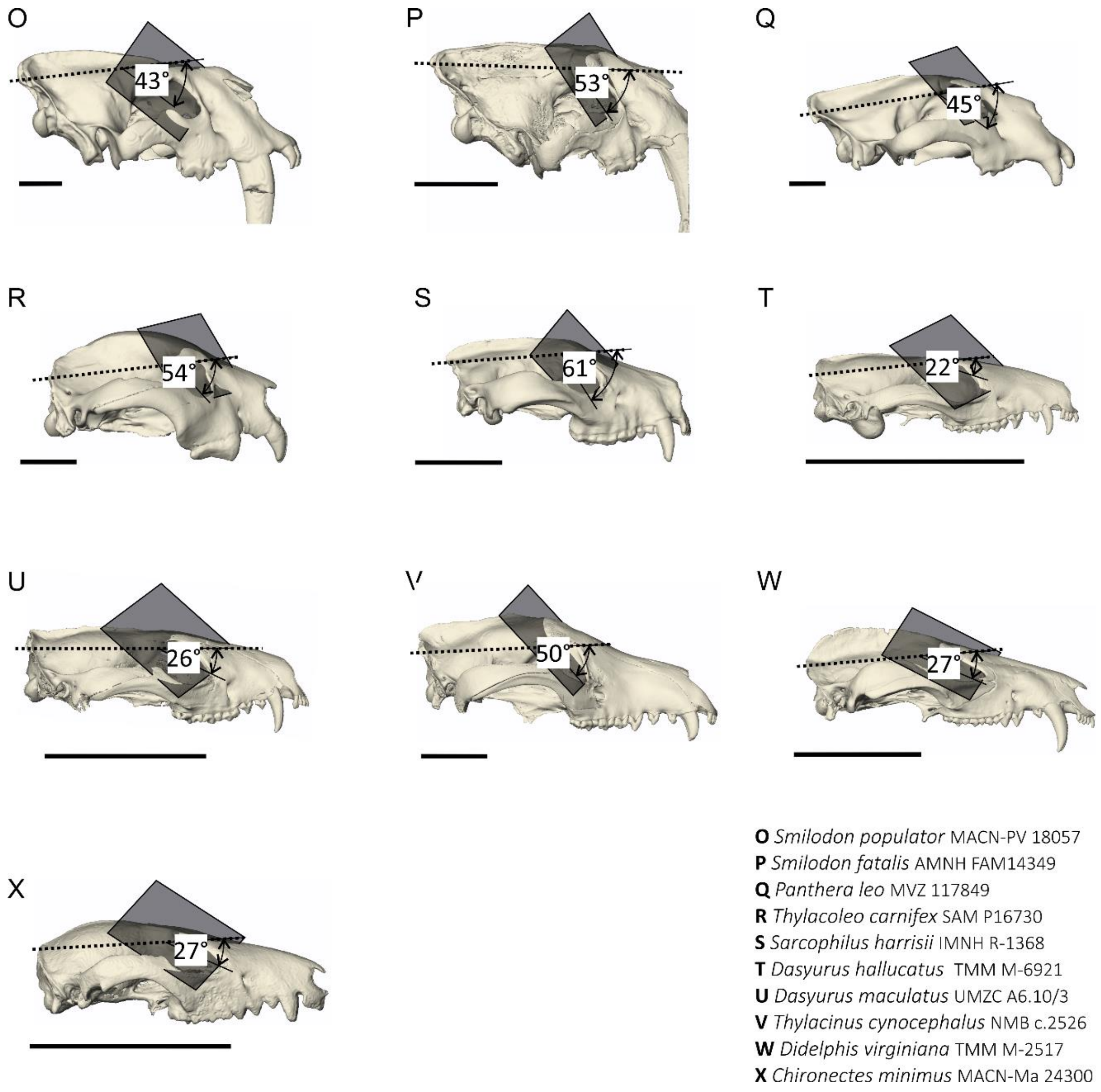

Figure S17. Orbital frontation for comparative sample, following method of Heesy (2005). Scale bar is 5 cm. Dotted line represents graphic simplification of inion-nasion line.

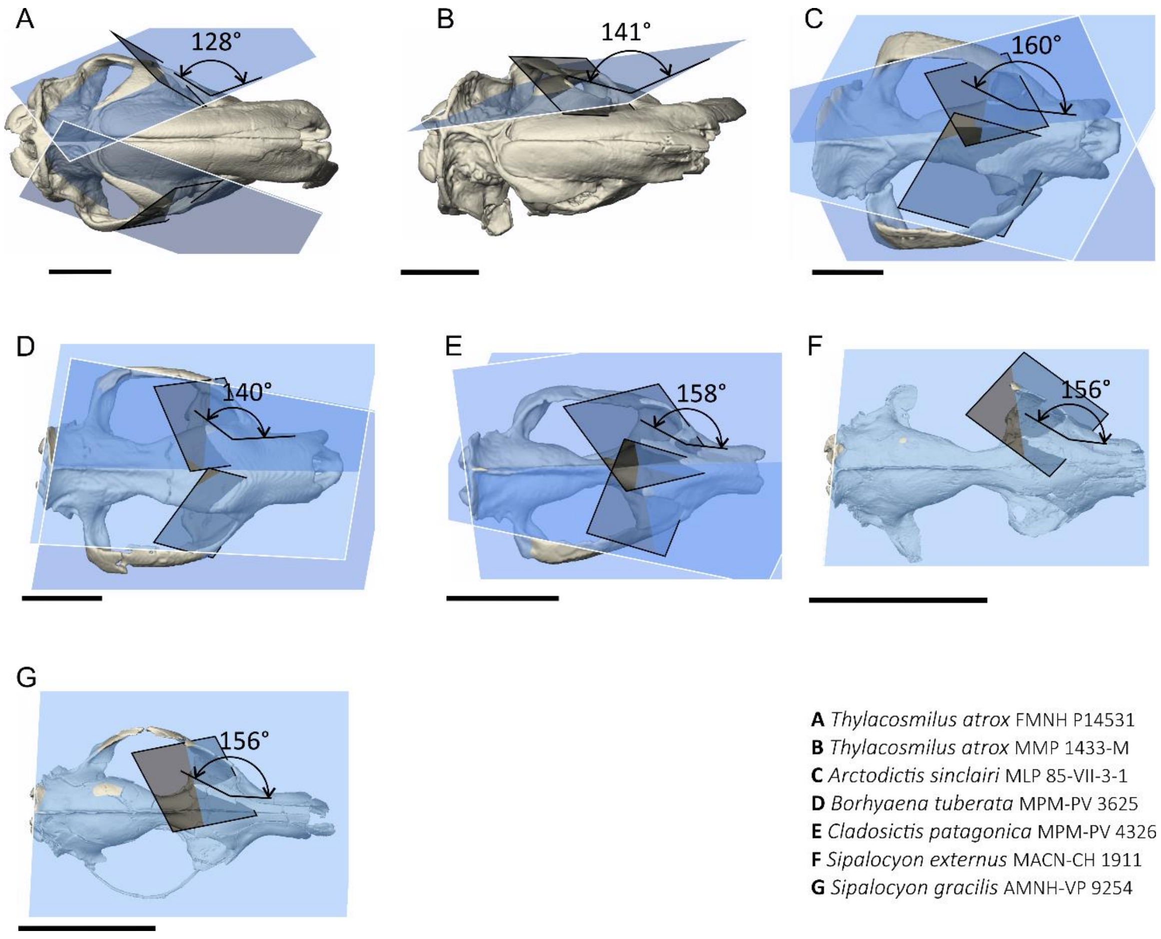

Figure S18. Orbitotemporal angle for sparassodonts, following method of Heesy (2005). Scale bar is 5 cm.

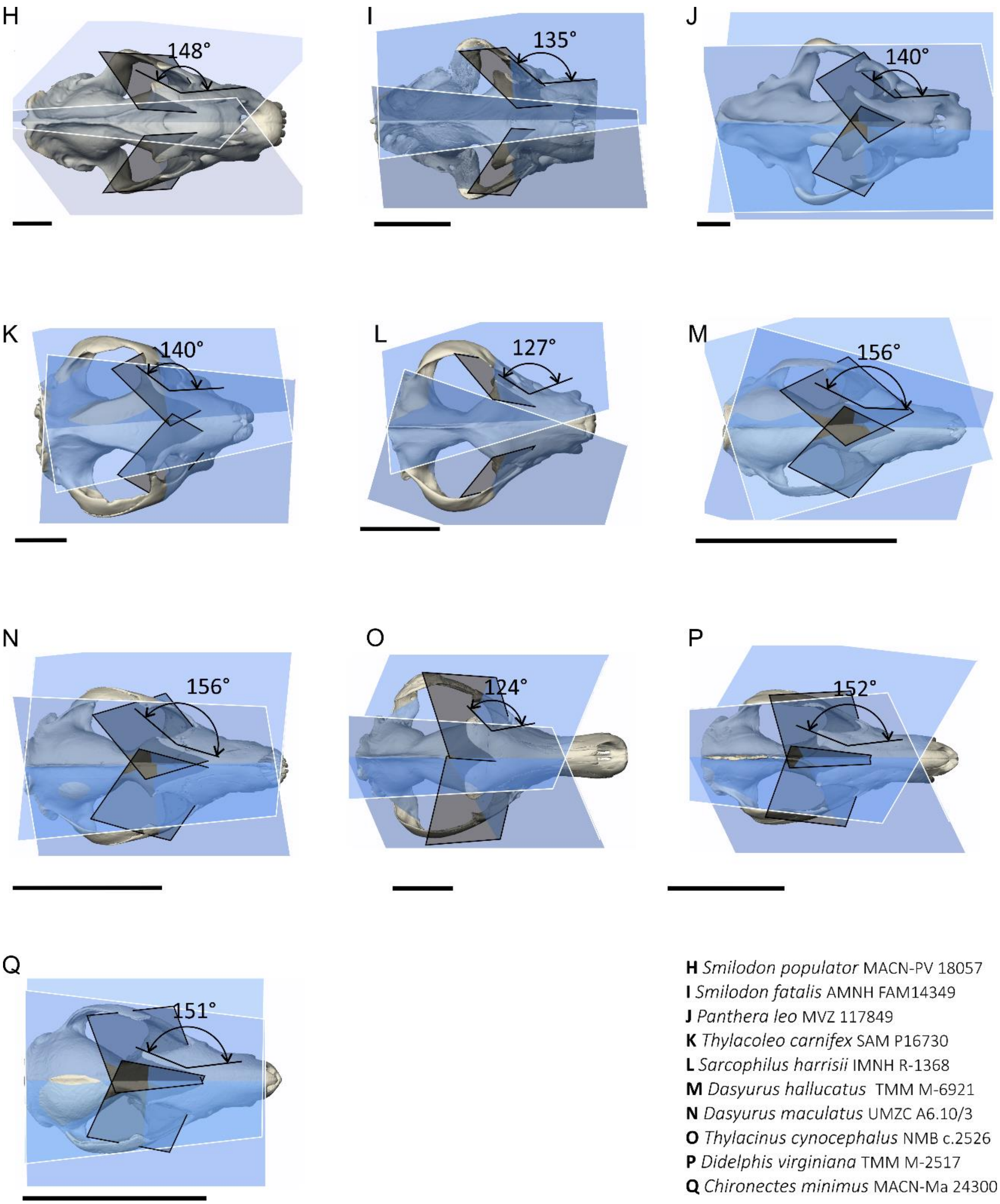

H *Smilodon populator* MACN-PV 18057
I *Smilodon fatalis* AMNH FAM14349
J *Panthera leo* MVZ 117849
K *Thylacoleo carnifex* SAM P16730
L *Sarcophilus harrisii* IMNH R-1368
M *Dasyurus hallucatus* TMM M-6921
N *Dasyurus maculatus* UMZC A6.10/3
O *Thylacinus cynocephalus* NMB c.2526
P *Didelphis virginiana* TMM M-2517
Q *Chironectes minimus* MACN-Ma 24300

Figure S19. Orbitotemporal angle for comparative sample, following method of Heesy (2005). Scale bar is 5 cm.

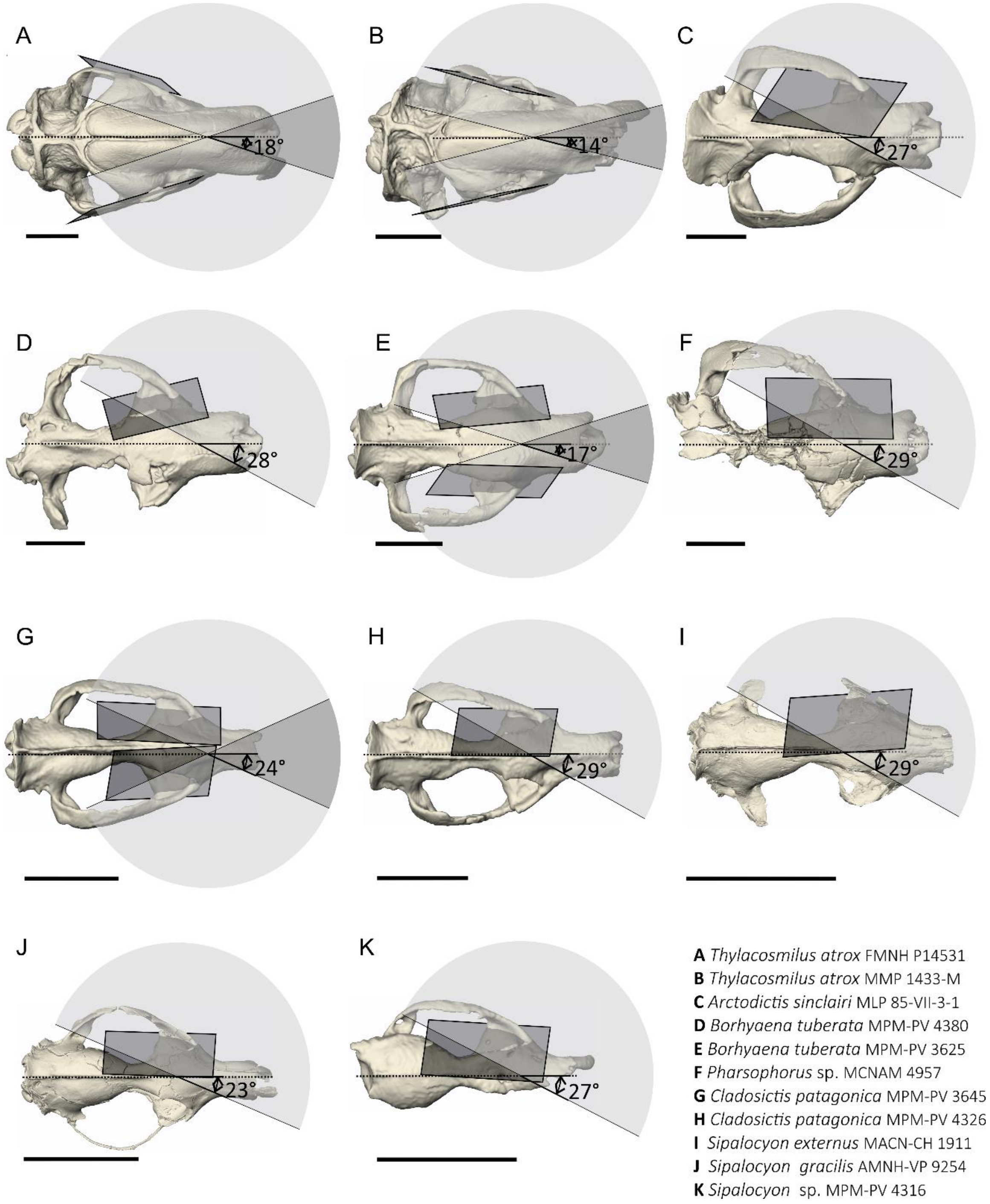

Figure S20. Orbital convergence for sparassodonts, following method of Finarelli and Goswami (2009). Scale bar is 5 cm. Dotted line represents sagittal plane.

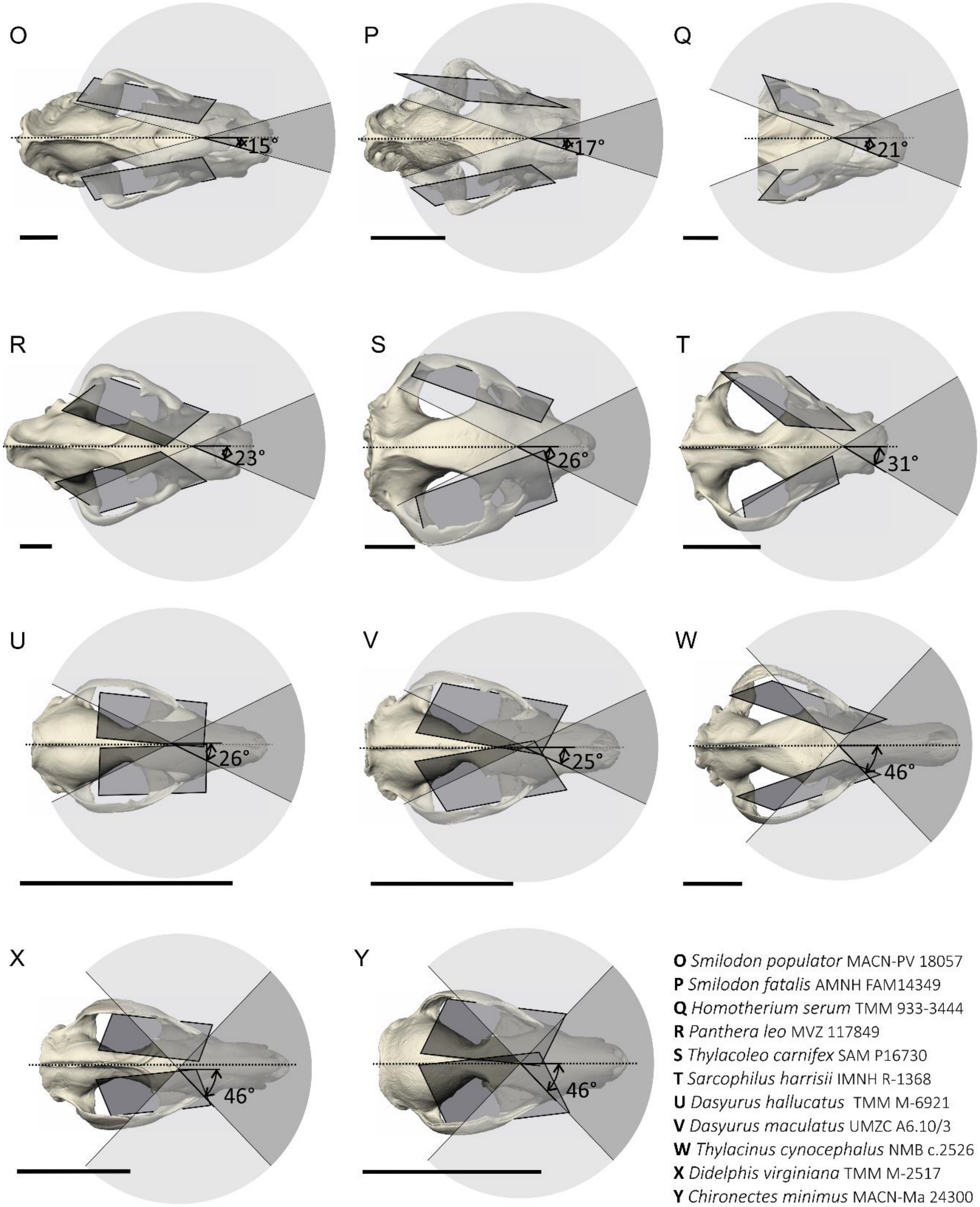

Figure S21. Orbital convergence for comparative sample, following method of Finarelli and Goswami (2009). Scale bar is 5 cm. Dotted line represents sagittal plane.

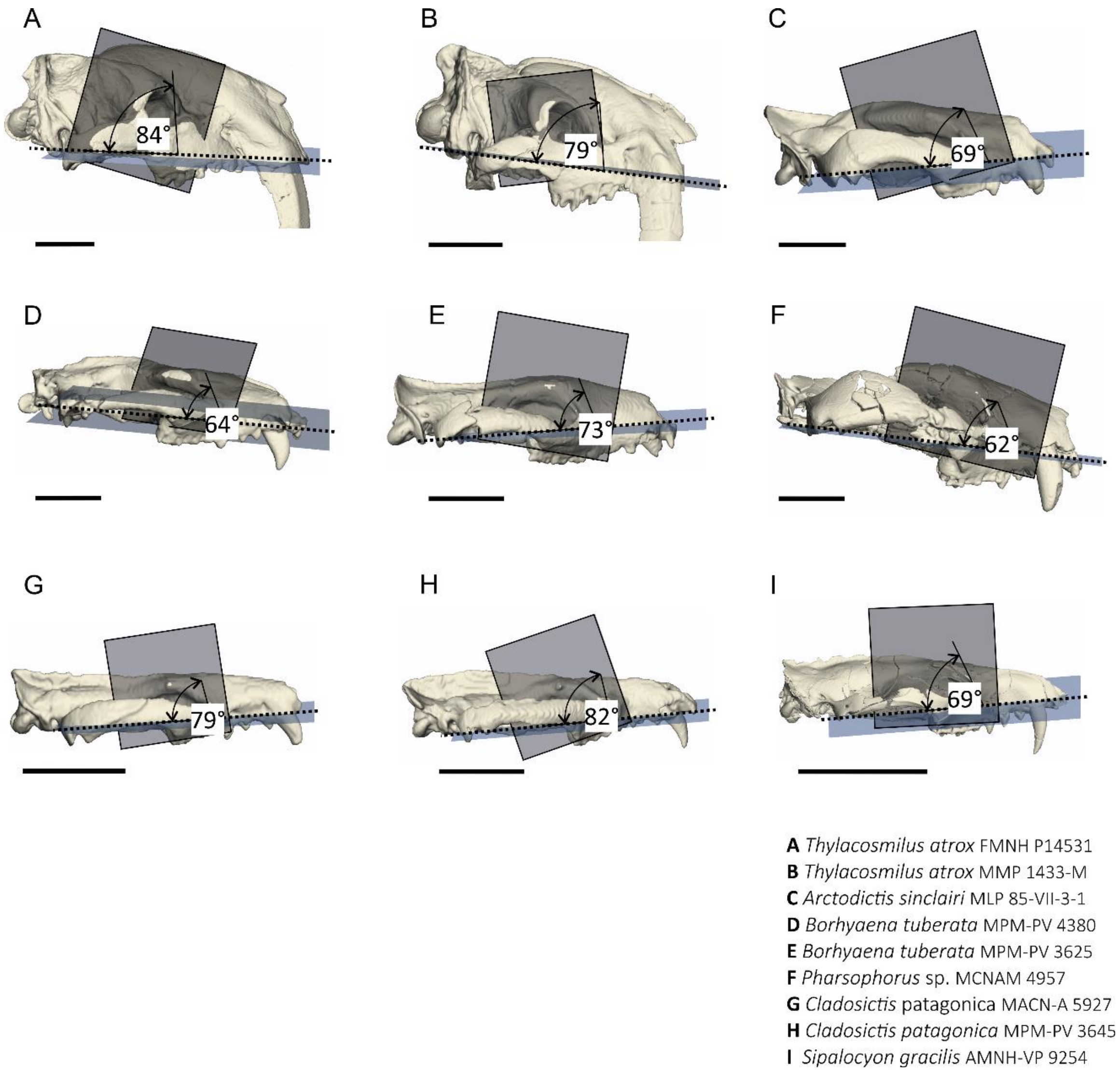

Figure S22. Orbital frontation for sparassodonts, following method of Finarelli and Goswami (2009). Scale bar is 5 cm. MLP 85-VII-3-1, MPM-PV 4380, MCNAM 4957, MPM-PV 3645, AMNH-VP 9254 are left side (reversed). Dotted line represents graphic simplification of basal plane (in blue).

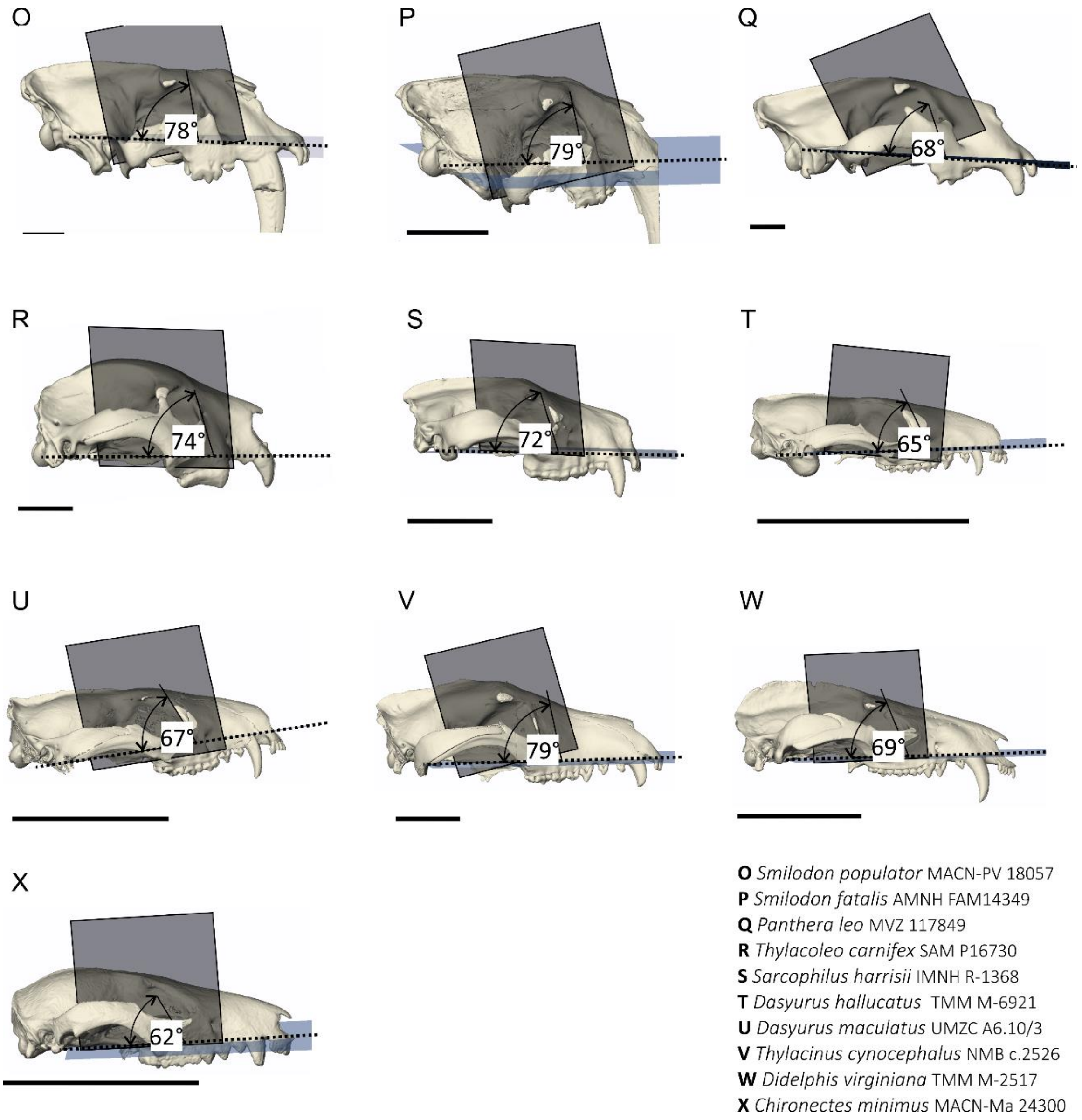

Figure S23. Orbital frontation for comparative sample, following method of Finarelli and Goswami (2009). Scale bar is 5 cm. Dotted line represents graphic simplification of basal plane (in blue).

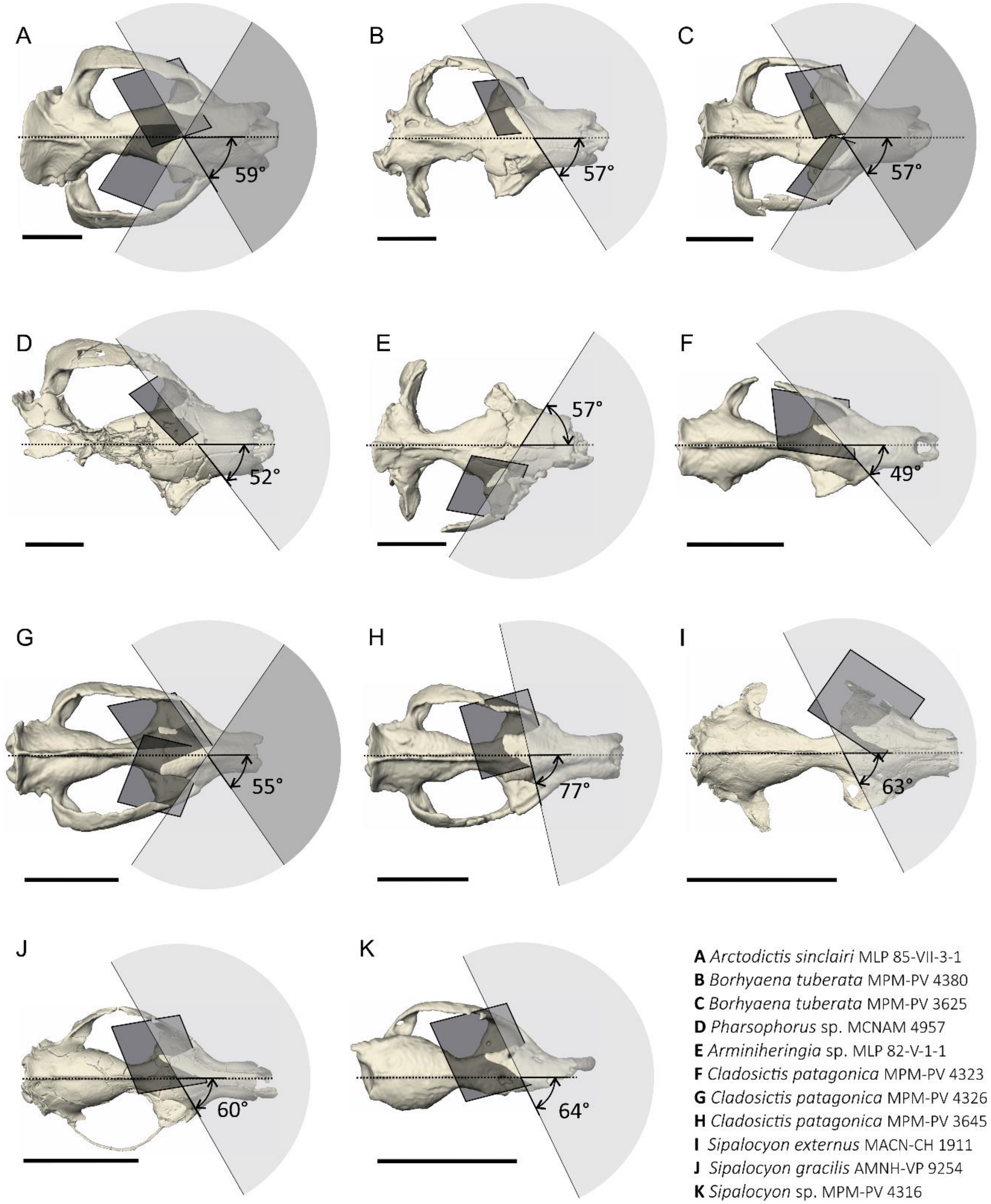

Figure S24. Orbital convergence for sparassodonts, following method of Pilatti and Astúa (2017). Scale bar is 5 cm. Dotted line represents sagittal plane.

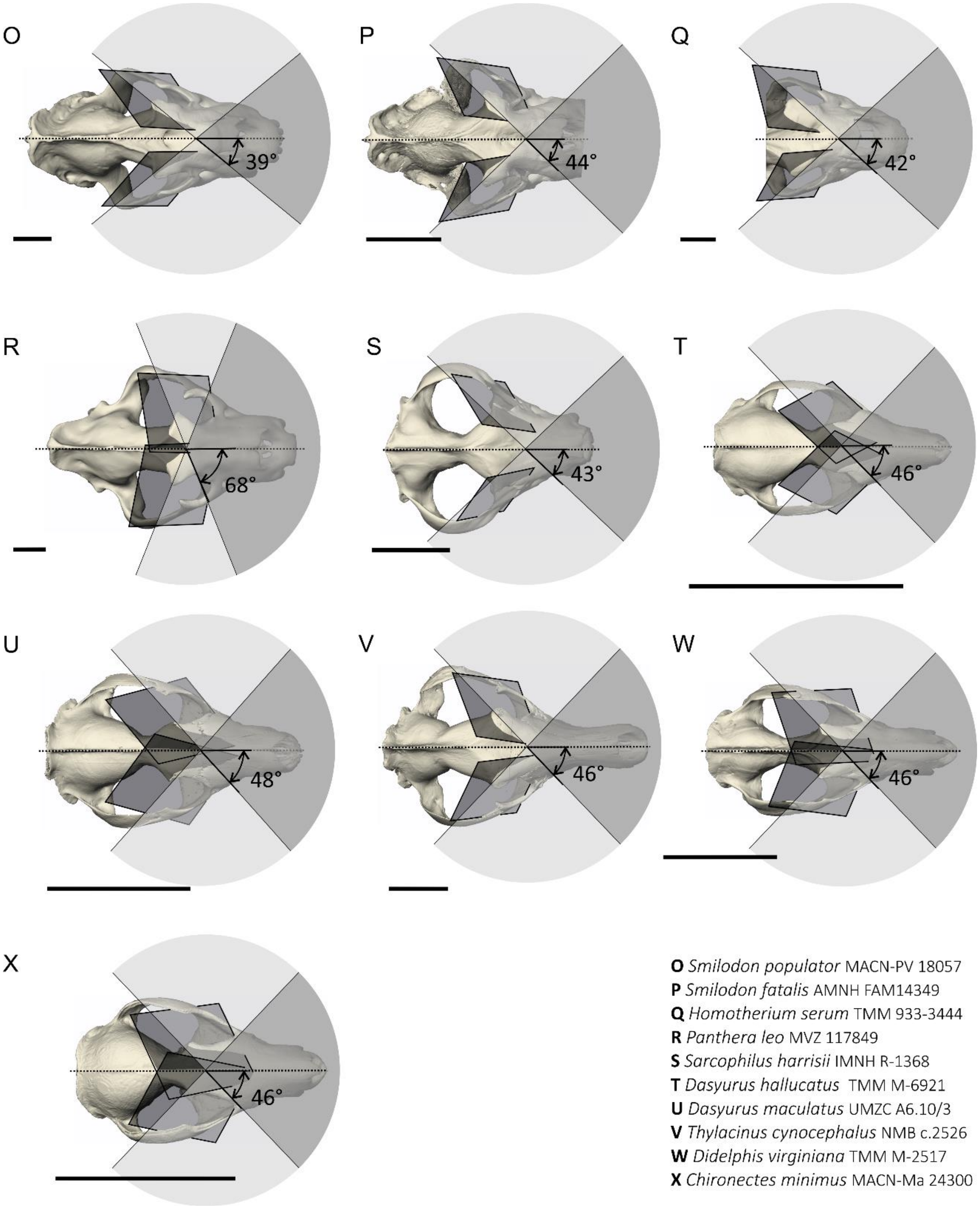

Figure S25. Orbital convergence for comparative sample, following method of Pilatti and Astúa (2017). Scale bar is 5 cm. Dotted line represents sagittal plane.

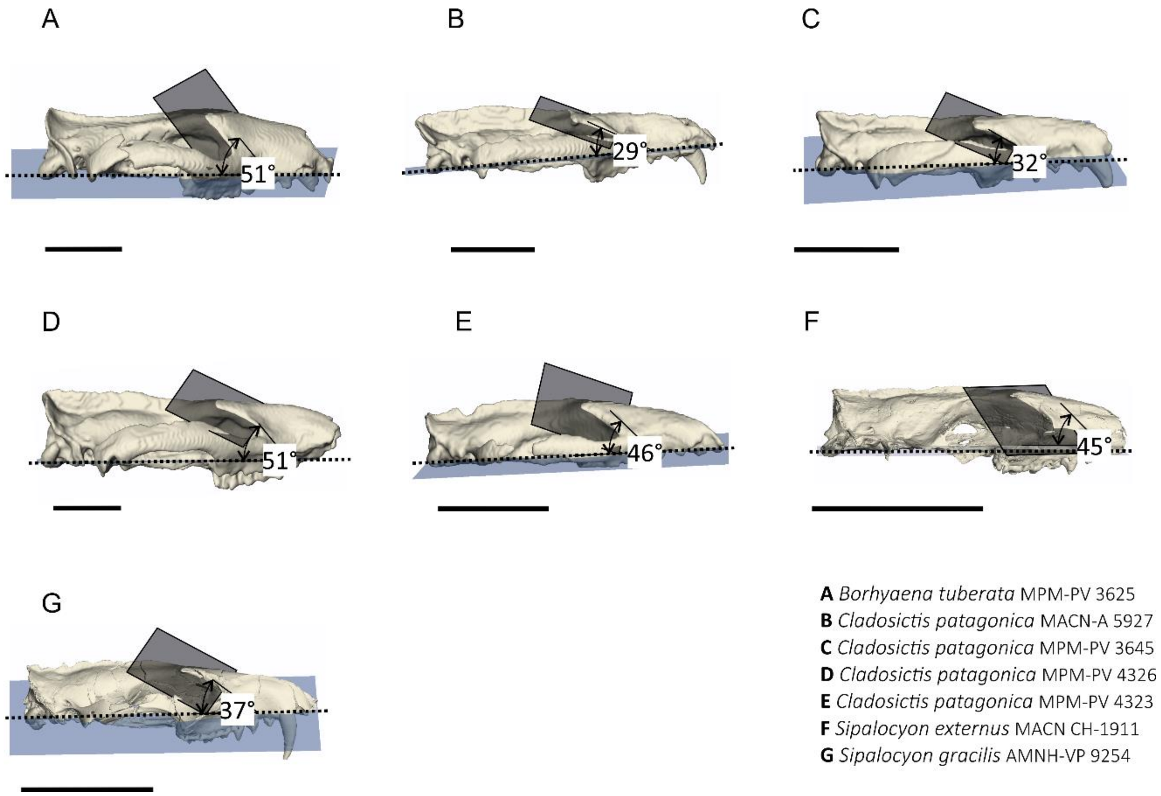

Figure S26. Orbital verticality for sparassodonts, following method of Pilatti and Astúa (2017). Scale bar is 5 cm. MPM-PV 3645, MPM-PV 4323, MACN Ch-1911, and AMNH-VP 9254 is left side (reversed). Dotted line represents graphic simplification of frontal plane (in blue).

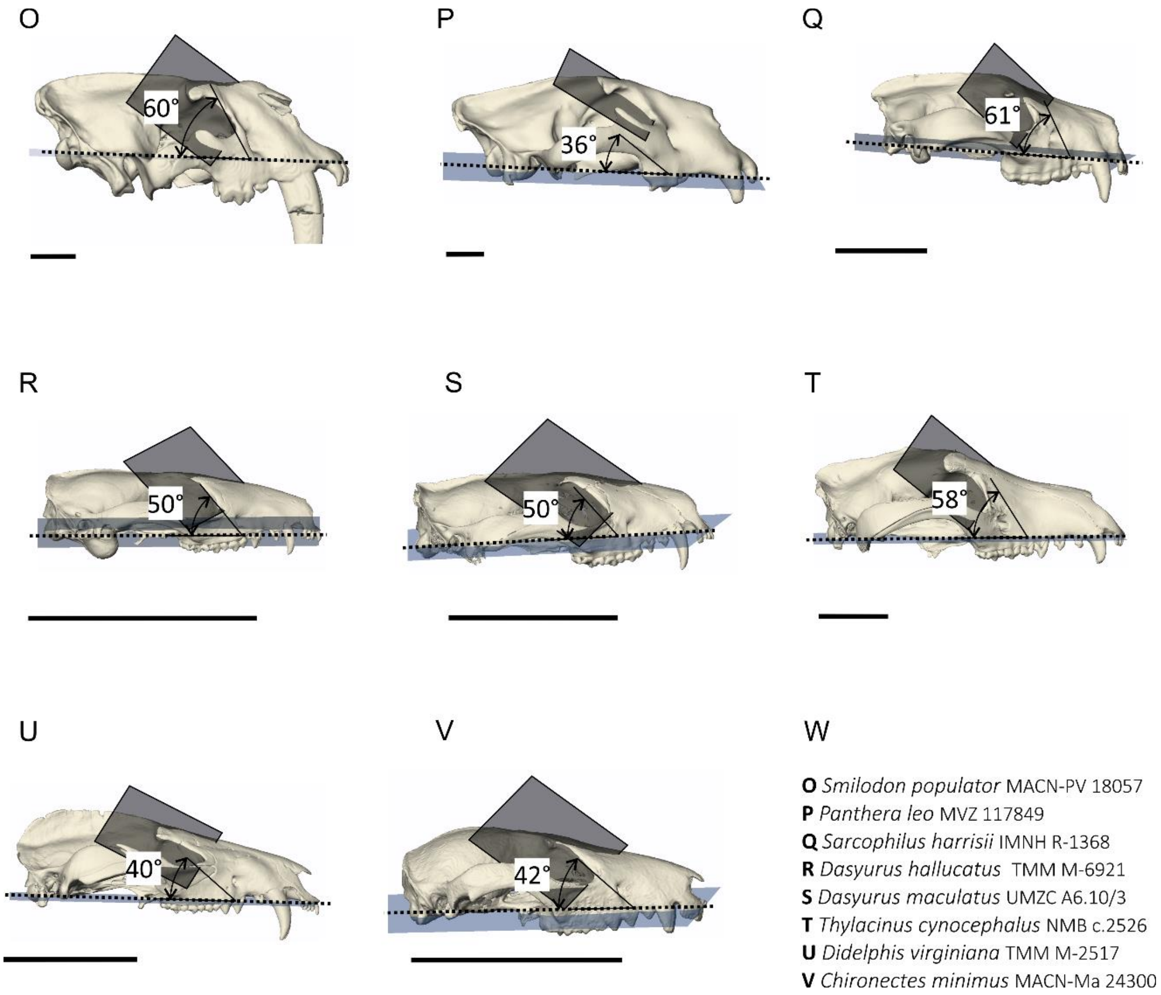

Figure S27. Orbital verticality for comparative sample, following method of Pilatti and Astúa (2017). Scale bar is 5 cm. Dotted line represents graphic simplification of frontal plane (in blue).

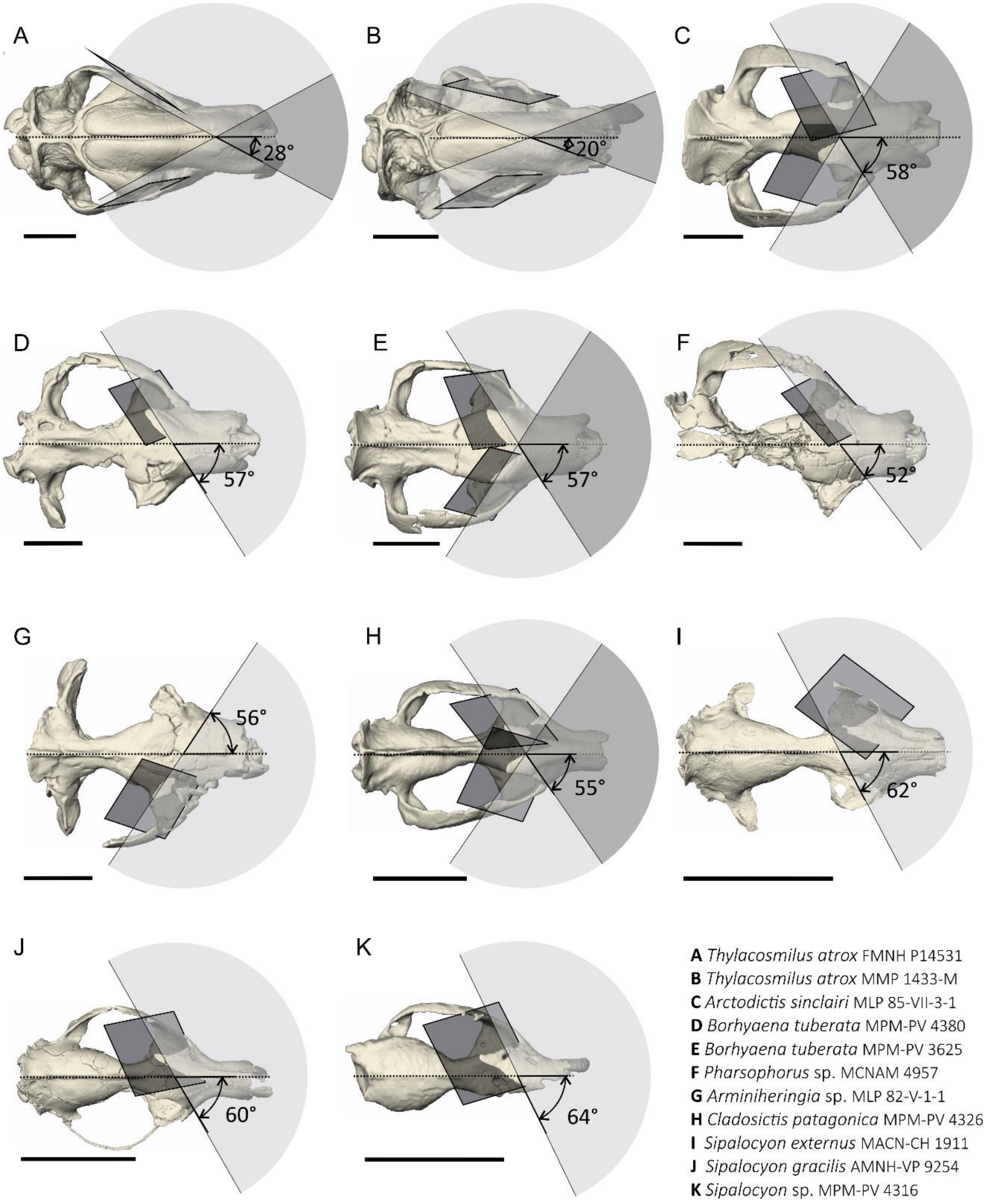

Figure S28. Orbital convergence for sparassodonts, following method of Casares-Hidalgo et al. (2019). Scale bar is 5 cm. Dotted line represents sagittal plane.

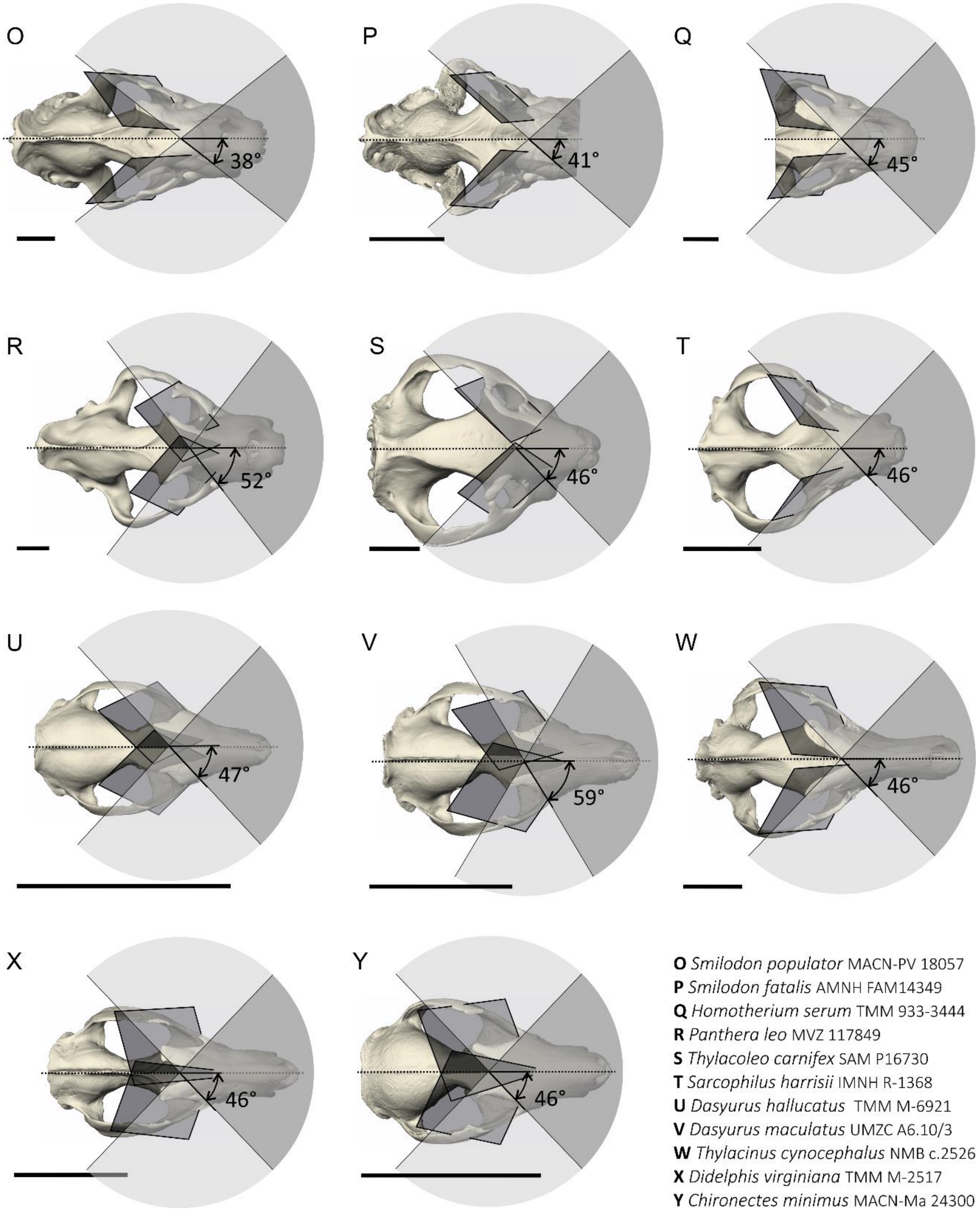

Figure S29. Orbital convergence for comparative sample, following method of Casares-Hidalgo et al. (2019). Scale bar is 5 cm. Dotted line represents sagittal plane.

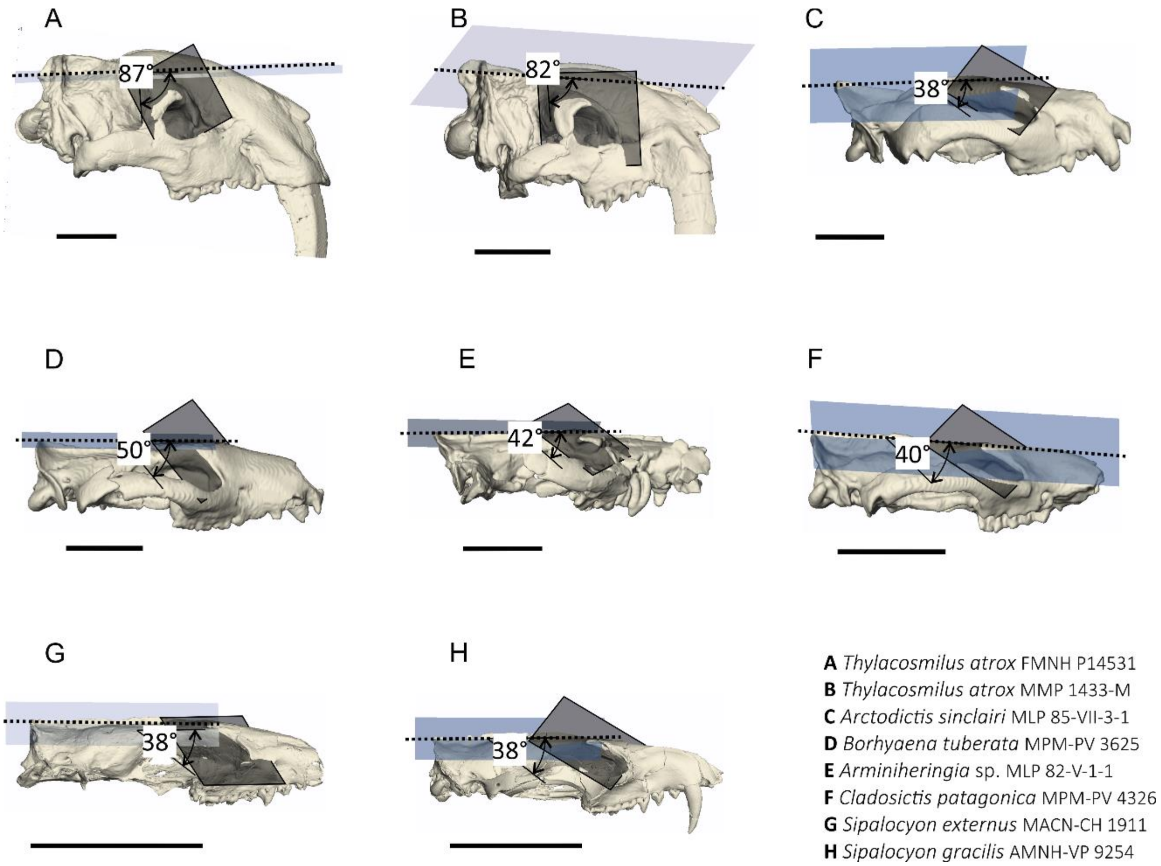

Figure S30. Orbital frontation for sparassodonts, following method of Casares-Hidalgo et al. (2019). Scale bar is 5 cm. MMP 1433-M, MLP 85-VII-3-1, MPM-PV 4323, MPM-PV 3645, MACN-CH 1911, and AMNH-VP 9254 is left side (reversed). Dotted line represents graphic simplification of dorsal plane (in blue).

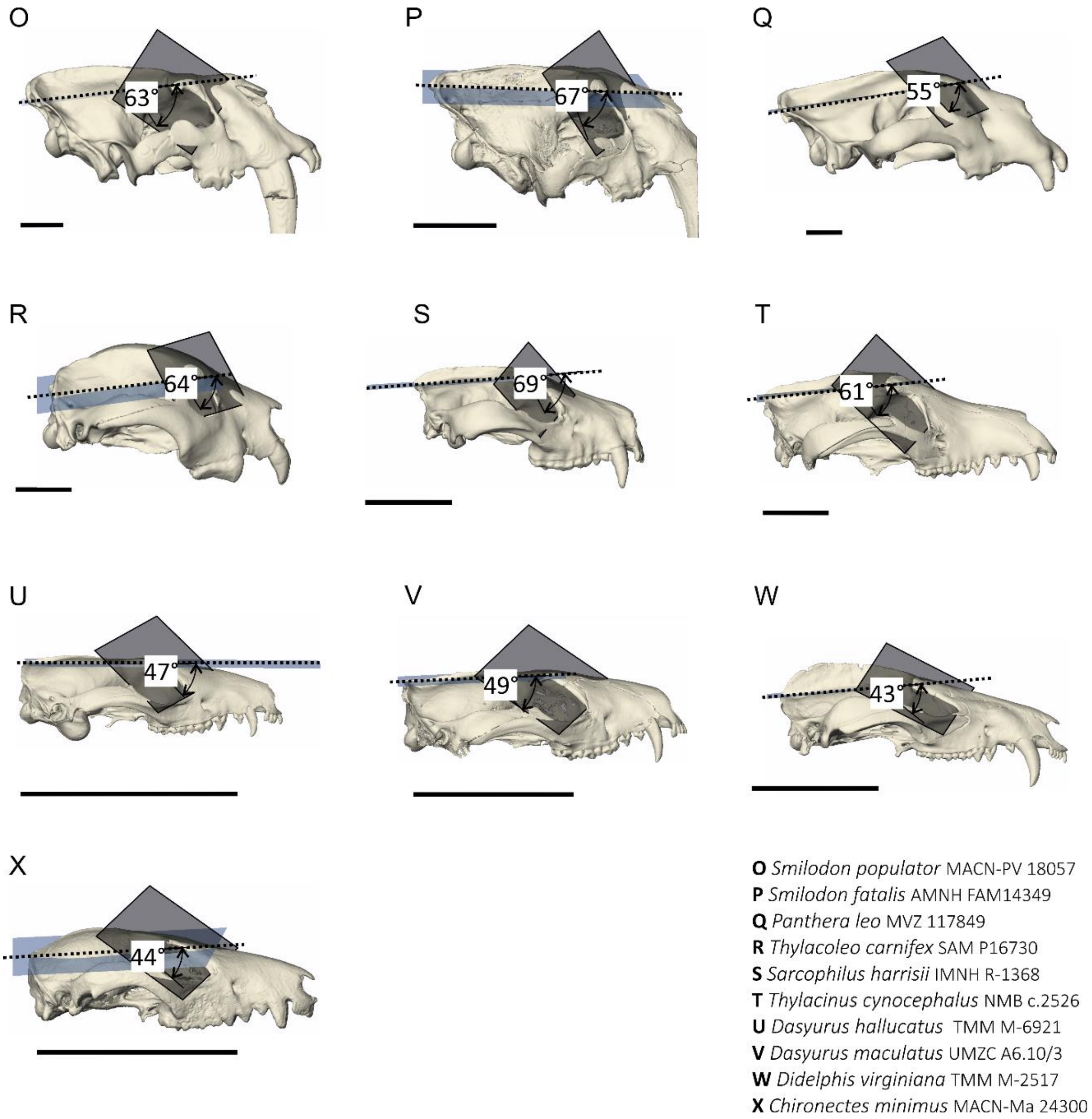

Figure S31. Orbital frontation for comparative sample, following method of Casares-Hidalgo et al. (2019). Scale bar is 5 cm. Dotted line represents graphic simplification of dorsal plane (in blue).

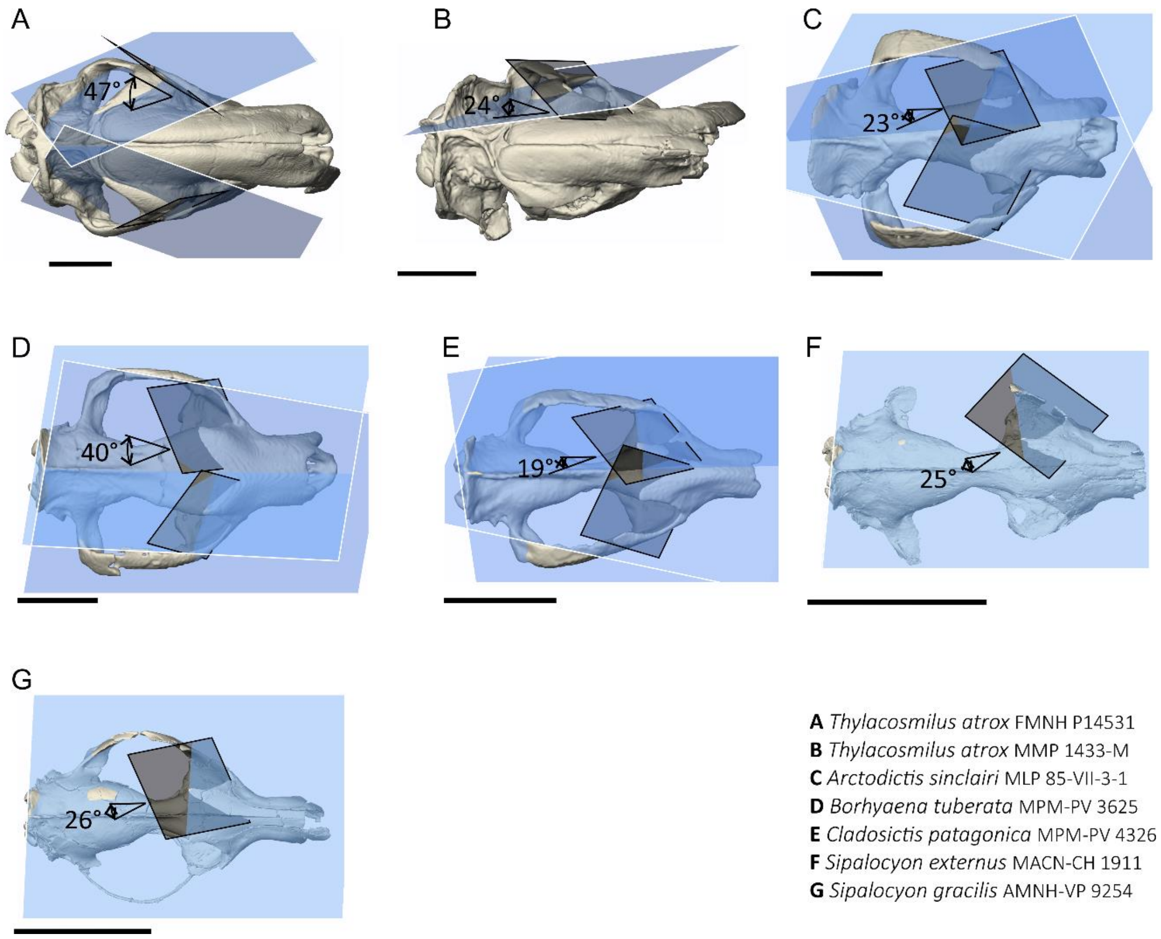

Figure S32. Orbitotemporal angle for sparassodonts, following method of Casares-Hidalgo et al. (2019). Scale bar is 5 cm.

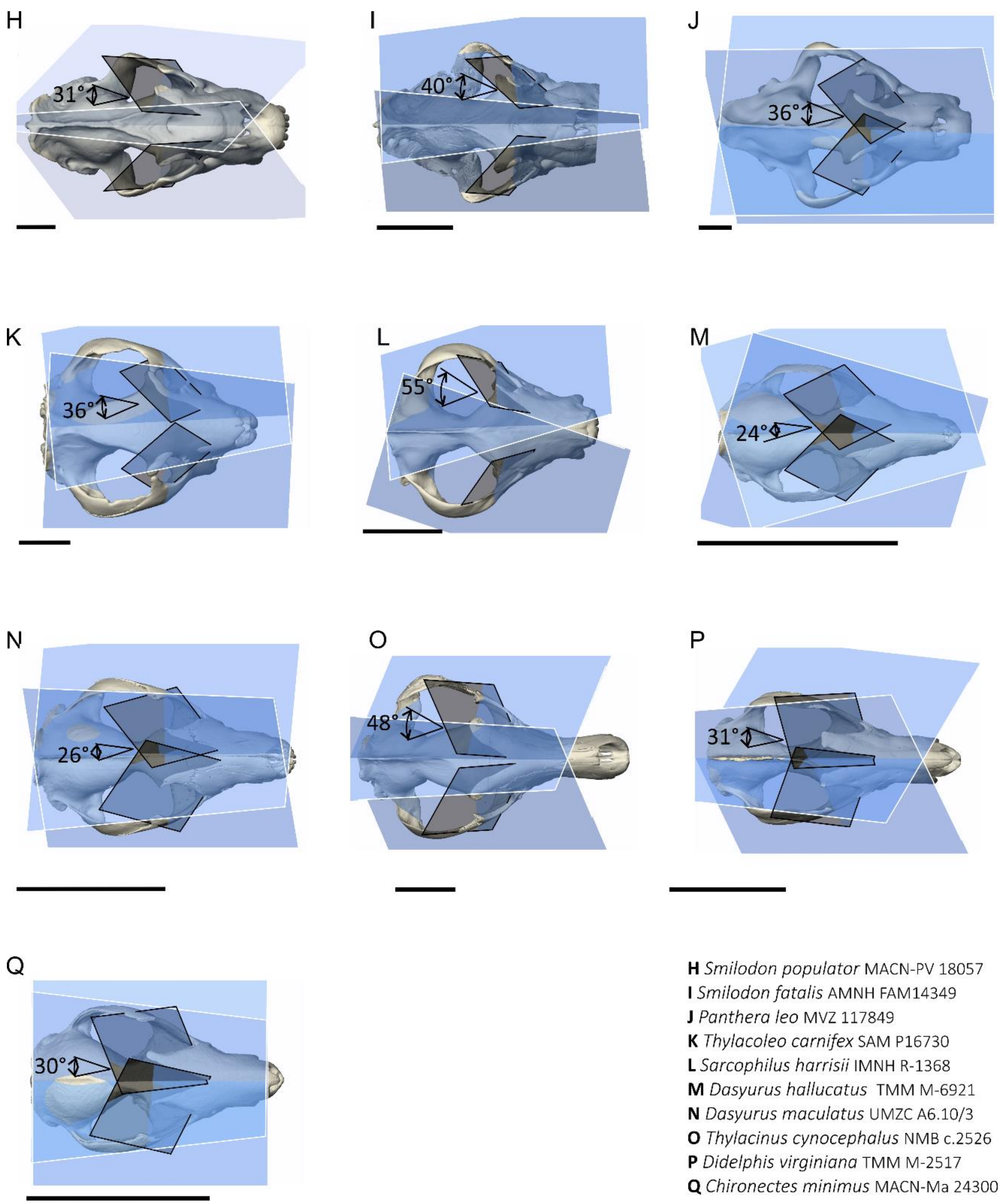

Figure S33. Orbitotemporal angle for comparative sample, following method of Casares-Hidalgo et al. (2019). Scale bar is 5 cm.

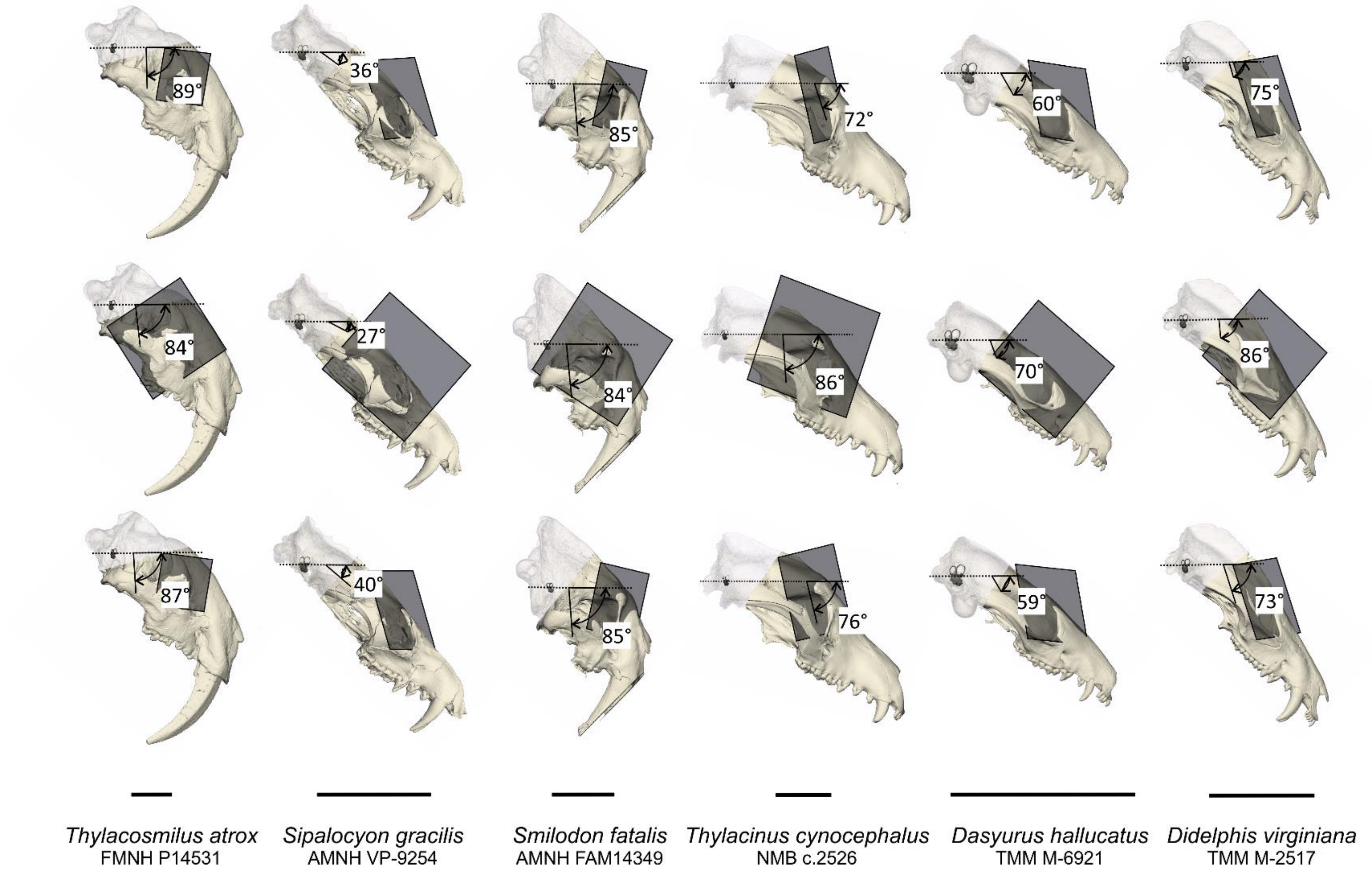

Figure S34. Gaze orientation in relation to head posture, as indicated by orbitolabyrinth angle. Scale bar is 5 cm. AMNH-VP 9254 is left side reversed.

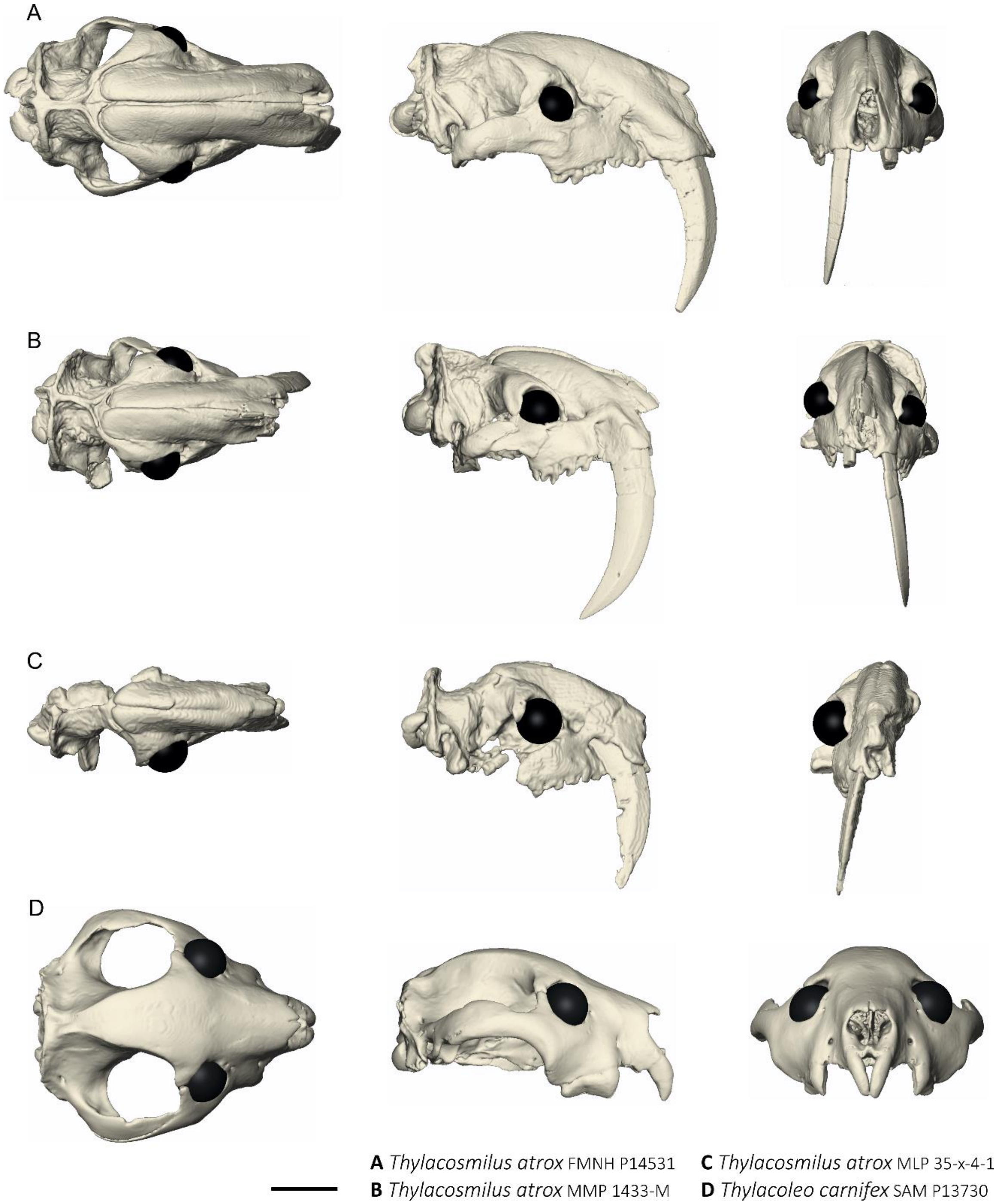

Figure S35. Virtual eye reconstruction for three specimens of *Thylacosmilus atrox* and one of *Thylacoleo carnifex*. Scale bar is 5 cm. MMP 1433-M and SAM P13730 are left side reversed.

Table S1. Scan parameters for specimens. The total slices correspond to stack in transverse axis. Reference to the corresponding scanning institution and scan provider for third party specimens is listed here (see full reference in the section SI references).

Taxon	Specimen	Type	Slice thickness [mm]	Voltage [kV]	Amper [μA]	Slice resolution [pxl]	Total slices	Reference
Thylacosmilus atrox	FMNH P14531	CT	0.625	120	200	307 × 471	433	Wroe et al. [80]
Thylacosmilus atrox	FMNH P14344	CT	0.070	210	190	1493 × 1180	2022	Forasiepi et al. [51]
Thylacosmilus atrox	MMP 1433-M	CT	0.450	120	460	648 × 428	546	
Thylacosmilus atrox	MLP 35-X-4-1	CT	0.400	120	271	209 × 104	1072	
Cladosictis patagonica	MPM-PV 3645	CT	0.400	120	271	105 × 64	393	
Cladosictis patagonica	MACN-A 5927	CT	0.400	120	271	194 × 72	468	
Cladosictis patagonica	MPM-PV 4323	CT	0.400	120	271	177 × 79	370	
Cladosictis patagonica	MPM-PV 4326	CT	0.400	120	313	240 × 88	404	
Sipalocyon externus	MACN-CH 1911	CT	0.049	135	300	1086 × 743	1994	
Sipalocyon gracilis	AMNH VP 9254	CT	0.069	170	190	1037 × 593	1648	Forasiepi et al. [51]
Sipalocyon sp.	MPM-PV 4316	CT	0.400	120	313	77 × 78	263	
Pharsophorus sp.	MCNAM 4957	CT	0.625	120	170	512 × 512	475	
Arminiheringia sp.	MLP 82-V-1-1	CT	0.625	120	271	512 × 512	359	
Arctodictis sinclairi	MLP 85-VII-3-1	CT	0.400	120	271	182 × 101	587	
Borhyaena tuberata	MPM-PV 4380	CT	0.400	120	271	258 × 109	638	
Borhyaena tuberata	MPM-PV 3625	CT	0.400	120	271	163 × 103	540	
Didelphis virginiana	TMM M-2517	CT	0.132	180	133	1024 × 1024	859	Macrini [108]
Chironectes minimus	MACN-Ma 24300	CT	0.194	30	240	285 × 285	571	
Dasyurus hallucatus	TMM M-6921	CT	0.078	180	133	1024 × 1024	781	Macrini [109]
Dasyurus maculatus	UMZC A6.10/3	CT	0.104	170	150	1578 × 892	2726	Martín-Serra and Benson [112]
Sarcophilus harrisii	IMNH R-1368	3D model	-	-	-	-	-	http://morphosource.org
Thylacinus cynocephalus	NMB c.2526	CT	0.072	180	90	3052 × 3058	3612	
Thylacoleo carnifex	SAM P16730	3D model	-	-	-	-	-	Wroe [114]
Panthera leo	MVZ 117849	3D model	-	-	-	-	-	Owen [111]
Panthera leo	PRICT 1434	CT	1.000	-	-	512 × 512	725	http://dmm.pri.kyoto-u.ac.jp
Panthera leo	PRICT 1435	CT	1.000	-	-	512 × 512	745	http://dmm.pri.kyoto-u.ac.jp
Smilodon populator	MACN-PV 18057	CT	0.500	120	271	250 × 288	816	
Smilodon fatalis	AMNH FAM14349	CT	0.142	170	-	994 × 994	1170	Tseng et al. [113]
Homotherium serum	TMM 933-3444	CT	0.650	419	1800	1024 × 1024	495	van Valkenburgh [110]

Table S2. Landmark definition and illustration of angle measurements according to the four methods followed in the study (exemplified by the dasyuromorph *Thylacinus cynocephalus* NMB c.2526).

	Heesy, 2005	Finarelli and Goswami, 2009	Pilatti and Astúa, 2017	Casares-Hidalgo et al., 2019
Orbital plane	orbitale superius<br><br>orbitale anterius<br><br>orbitale inferius	postorbital process of the frontal bone<br><br>dorsal suture of the jugal and maxilla<br><br>ventral suture of the jugal and maxilla	postorbital process of the frontal bone<br><br>between the two lacrimal foramina of the lacrimal bone<br><br>tip of the frontal process of the jugal	orbitale superius<br><br>orbitale anterius<br><br>orbitale posterius
	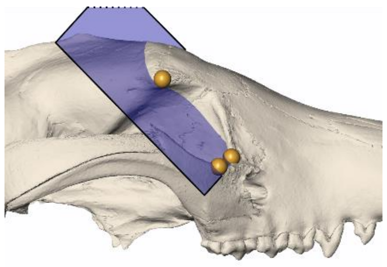	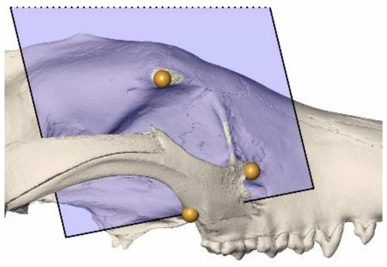	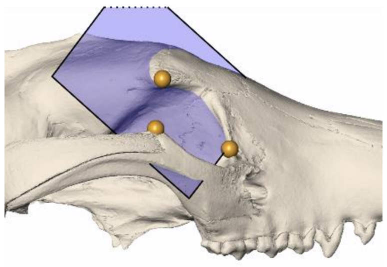	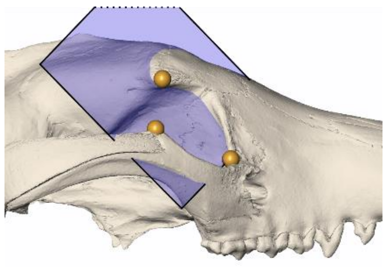
Convergence	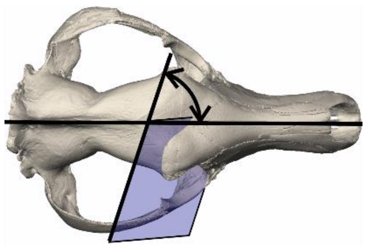	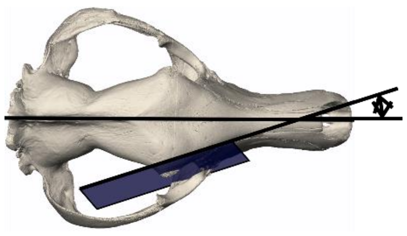	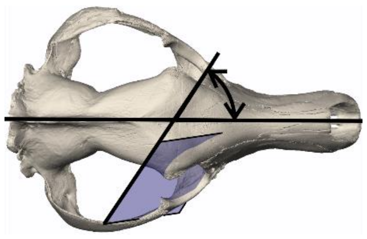	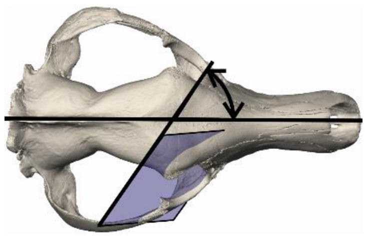
Plane definition used for convergence (sagittal plane)	**midsagittal plane**<br>prosthion<br><br>nasion<br><br>inion	**midsagittal plane**<br>maxilla-premaxilla midline ventral suture<br>nasal anterior<br><br>maxilla-palatine midline suture<br>nasal-frontal suture<br>parietal-frontal suture<br>parietal-occipital suture	**sagittal plane**<br>anterior extremity of the internasal suture<br>posterior edge of sagittal crest on the midline of interparietal bone	**sagittal plane**<br>prosthion<br><br>nasion<br><br>inion<br>basion
Verticality	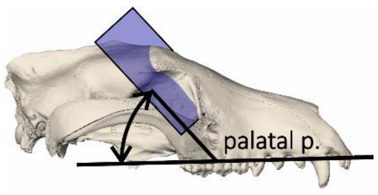		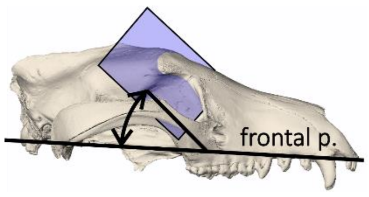	
Plane definition used for verticality	**Palatal plane**<br><br>alveolar margin directly above the middle of the left first upper molar<br><br>alveolar margin directly above the middle of the right first upper molar<br>prosthion		**Frontal plane**<br><br>maximum curvature of the posterolateral surface of the occipital condyle<br>margin of the alveolar process of first upper incisor in premaxilla	
Frontation<br><br>Plane definition used for frontation	nasion-inion chord – intersection of orbital plane and sagittal plane	**Basal plane**<br><br>premaxilla-maxilla lateral sutures<br><br>basioccipital-basisphenoid sutures		**Dorsal plane**<br><br>perpendicular to sagittal plane
	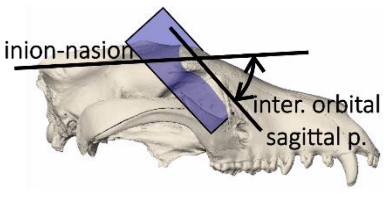	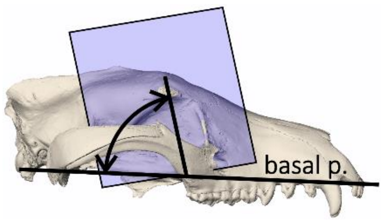		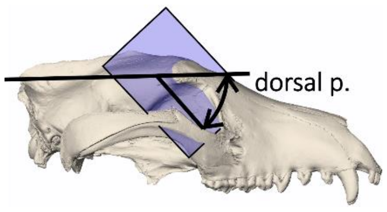
Orbitotemporal angle<br>Plane definition used for orbitotemporal angle	**Temporal plane**<br><br>orbital superius<br><br>orbital lateralis<br>most posterior point of the temporal fossa			**Temporal plane**<br><br>complementary angle of Heesy (2005)
	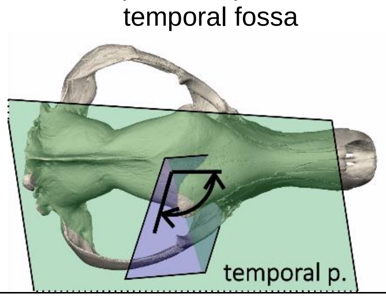			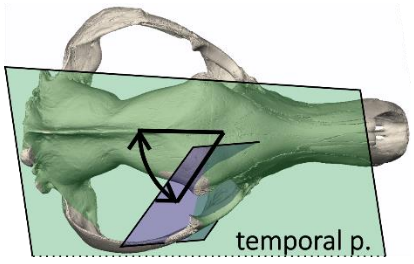

Table S3. Results of measurements for the specimens studied. Abbreviations: **CH19**, Casares-Hidalgo et al. (2019); **H05**, Heesy (2005); **F&G09**, Finarelli and Goswami (2009); **L**, left; **P&A17**, Pilatti and Astúa (2017); **R**, right; **1**, Ross and Kirk (2007); **2**, Howland et al. (2004).

Taxonomy	Taxon	Collection number	Convergence angle [°]								Verticality angle [°]				Frontation angle [°]						Orbitotemporal angle [°]				Postorbital gap [mm]	
			H05		F&G09		P&A17		CH19		H05		P&A17		H05		F&G09		CH19		H05		CH19		H05	
			L	R	L	R	L	R	L	R	L	R	L	R	L	R	L	R	L	R	L	R	L	R	L	R
Thylacosmilidae	*Thylacosmilus atrox*	FMNH P-14531	39.6	30.0	18.9	17.7	/	/	33.7	21.2	88.2	82.3	/	/	85.8	72.6	81.6	85.7	88.4	85.5	121.2	135.3	55.5	39.0	0.0	0.0
Thylacosmilidae	*Thylacosmilus atrox*	MMP 1433-M	33.7	27.7	13.1	14.5	/	/	14.6	24.3	62.9	83.1	/	/	55.2	69.8	79.9	78.3	81.9	83.0	141.0	-	23.4	-	0.0	0.0
Borhyaenidae	*Arctodictis sinclairi*	MLP 85-VII-3-1	53.0	53.1	26.6	-	60.0	58.4	59.2	57.2	55.2	41.2	-	-	19.7	20.6	69.0	-	37.1	38.9	157.9	162.9	26.2	19.7	23.5	39.0
Borhyaenidae	*Borhyaena tuberata*	MPM-PV 4380	65.0	51.2	28.0	-	56.8	-	57.2	-	43.8	62.2	-	-	-	-	64.0	-	-	-	-	-	-	-	30.2	-
Borhyaenidae	*Borhyaena tuberata*	MPM-PV 3625	61.9	51.3	18.7	15.0	62.4	51.3	63.1	51.2	52.6	55.5	46.7	54.9	37.8	43.2	71.8	75.0	45.3	55.4	140.9	139.6	39.7	40.3	30.6	30.3
Borhyaenoidea	*Pharsophorus* sp.	MCNAM 4957	52.4	-	28.6	-	51.7	-	51.7	-	57.3	-	-	-	-	-	62.4	-	-	-	-	-	-	-	32.3	-
Borhyaenoidea	*Arminiheringia* sp.	MLP 82-V-1-1	-	51.2	-	-	-	56.5	-	56.3	-	40.0	-	-	-	25.1	-	-	-	42.3	-	-	-	-	-	26.4
Hathliacynidae	*Cladosictis patagonica*	MPM-PV 3645	76.9	-	28.6	-	77.1	-	76.6	-	44.2	-	31.8	-	17.6	-	78.5	-	21.9	-	161.4	-	18.4	-	12.4	-
Hathliacynidae	*Cladosictis patagonica*	MACN-A 5927	-	86.1	-	16.2	-	82.7	-	81.4	-	30.8	-	29.3	-	20.3	-	82.1	-	20.3	-	141.0	-	28.4	-	17.7
Hathliacynidae	*Cladosictis patagonica*	MPM-PV 4323	49.4	-	-	-	48.6	-	48.4	-	-	-	45.9	-	28.7	-	-	-	48.8	-	154.2	-	25.2	-	18.6	-
Hathliacynidae	*Cladosictis patagonica*	MPM-PV 4326	47.0	71.7	18.1	29.0	43.4	67.0	43.9	65.6	36.1	46.6	37.9	38.7	16.7	23.9	-	-	48.1	32.7	163.7	153.2	14.5	23.8	21.3	15.5
Hathliacynidae	*Sipalocyon externus*	MACN-CH 1911	60.7	-	28.9	-	62.5	-	62.4	-	44.6	-	44.7	-	26.3	-	-	-	37.7	-	155.9	-	25.2	-	28.8	-
Hathliacynidae	*Sipalocyon gracilis*	AMNH-VP 9254	54.3	-	23.0	-	60.1	-	60.2	-	51.5	-	37.1	-	22.7	-	68.7	-	37.6	-	156.4	-	25.8	-	15.7	-
Hathliacynidae	*Sipalocyon* sp.	MPM-PV 4316	59.5	-	27.3	-	63.6	-	63.6	-	-	-	-	-	-	-	-	-	-	-	-	-	-	-	13.3	-
Felidae	*Smilodon populator*	MACN-PV 18057	38.9	40.3	14.8	14.6	38.6	38.4	37.7	38.7	61.1	66.7	58.4	60.6	41.2	45.4	75.9	79.1	62.6	64.1	149.6	147.2	29.6	31.8	39.5	38.4
Felidae	*Smilodon fatalis*	AMNH FAM14349	40.6	52.2	16.7	17.0	43.3	44.9	38.1	43.3	-	-	-	-	51.5	54.3	80.4	76.9	67.3	66.2	140.2	130.5	38.0	42.5	19.8	22.8
Felidae	*Homotherium serum*	TMM 933-3444	53.4	46.4	17.5	25.0	42.5	41.0	46.8	42.4	51.0	60.8	-	-	-	-	-	-	-	-	-	-	-	-	36.6	41.3
Felidae	*Panthera leo*	MVZ 117849	56.0	57.9	25.1	21.6	69.4	67.2	53.4	50.7	48.9	45.6	37.9	34.8	44.8	46.0	66.1	70.2	54.3	55.4	142.3	138.7	35.6	35.8	29.7	32.2
Felidae	*Panthera leo*	PRICT 1434	54.6	59.6	23.9	26.7	71.5	75.2	54.8	59.6	48.8	47.1	38.0	36.7	41.8	37.6	69.9	70.4	52.5	46.9	138.6	139.6	41.6	40.4	30.5	29.3
Felidae	*Panthera leo*	PRICT 1435	65.4	66.1	24.8	28.3	72.4	75.1	55.1	57.7	48.1	46.7	36.8	36.5	47.1	43.7	69.8	68.2	54.1	48.0	128.6	133.1	44.4	38.6	30.7	29.1
Diprotodontia	*Thylacoleo carnifex*	SAM P16730	45.1	54.5	21.9	30.3	/	/	43.9	47.4	60.1	62.3	/	/	49.9	57.2	73.2	73.9	67.6	60.4	139.0	141.6	40.1	32.9	0.0	0.0
Dasyuridae	*Dasyurus hallucatus*	TMM M-6921	41.8	51.4	25.9	26.1	45.9	47.0	47.0	47.7	50.8	47.6	47.1	53.4	18.8	25.7	65.3	64.1	47.8	47.1	158.6	154.1	23.6	23.8	10.8	10.8
Dasyuridae	*Dasyurus maculatus*	UMZC A6.10/3	43.5	46.0	24.9	25.9	45.1	49.3	46.0	49.1	52.5	53.6	54.8	45.6	27.0	24.4	66.1	67.6	51.2	47.0	154.2	156.9	26.7	24.8	13.3	12.9
Dasyuridae	*Sarcophilus harrisii*	IMNH R-1368	42.9	39.0	32.4	29.8	45.4	40.3	48.7	42.3	72.4	73.1	59.3	63.2	60.5	62.3	69.9	73.7	67.3	70.9	127.4	127.5	55.8	53.8	14.0	17.3
Thylacinidae	*Thylacinus cynocephalus*	NMB c.2526	61.4	66.2	19.3	19.4	52.7	46.4	53.4	46.9	57.6	52.8	57.9	57.4	50.5	49.3	81.7	75.9	59.8	62.6	125.5	121.8	49.6	46.5	20.3	19.5
Didelphidae	*Didelphis virginiana*	TMM M-2517	49.8	52.0	22.0	21.7	56.6	55.6	56.6	55.6	44.5	42.4	39.3	40.0	27.7	27.6	68.9	69.0	42.7	43.3	152.0	151.6	32.2	30.6	11.5	11.6
Didelphidae	*Chironectes minimus*	MACN-Ma 24300	43.5	50.0	27.7	29.7	54.9	56.1	54.9	56.0	48.2	49.6	40.6	44.0	26.1	28.7	61.5	62.5	44.1	44.4	151.6	150.9	29.4	30.9	9.6	9.1

Table S3. (Continued).

Taxonomy	Taxon	Collection number	Interorbital width [mm] [119]	Skull length [mm] [119]	Snout length [mm] [119]	Snout breadth [mm] [119]	Snout width [mm]	Rostrum relative length [119]	Rostrum proportional width [119]	Eyeball diameter [mm]	Eye-skull length ratio	Eye-rostrum relative length ratio	Eye-snout width ratio	residual sagittal-palatal angle [°]	residual sagittal-frontal angle [°]	residual sagittal-basal angle [°]
Thylacosmilidae	*Thylacosmilus atrox*	FMNH P14531	79.8	214.1	91.2	79.3	45.4	0.43	0.87	33.4	0.16	0.37	1.47	0.1	1.6	1.7
Thylacosmilidae	*Thylacosmilus atrox*	MMP 1433-M	56.7	178.1	75.3	59.6	42.6	0.42	0.79	33.3	0.19	0.44	1.56	14.4	22.0	10.1
Thylacosmilidae	*Thylacosmilus atrox*	MLP 35-X-4-1	-	172.1	-	-	-	-	-	35.3	0.20	-	-	-	-	-
Borhyaenidae	*Arctodictis sinclairi*	MLP 85-VII-3-1	52.8	185.1	42.2	74.8	48.8	0.23	1.8	-	-	-	-	9.0	-	5.5
Borhyaenidae	*Borhyaena tuberata*	MPM-PV 4380	-	-	-	-	54.4	-	-	-	-	-	-	7.1	-	0.1
Borhyaenidae	*Borhyaena tuberata*	MPM-PV 3625	32.2	167.9	50.4	63.5	47.4	0.30	1.26	-	-	-	-	4.2	2.3	0.0
Borhyaenoidea	*Pharsophorus* sp.	MCNAM 4957	-	-	-	-	-	-	-	-	-	-	-	0.9	-	0.6
Borhyaenoidea	*Arminiheringia* sp.	MLP 82-V-1-1	-	-	-	-	-	-	-	-	-	-	-	8.7	-	-
Hathliacynidae	*Cladosictis patagonica*	MPM-PV 3645	25.9	135.1	39.1	35.9	-	0.29	0.92	-	-	-	-	22.1	12.9	16.9
Hathliacynidae	*Cladosictis patagonica*	MACN-A 5927	-	-	-	-	-	-	-	-	-	-	-	7.8	10.1	8.1
Hathliacynidae	*Cladosictis patagonica*	MPM-PV 4323	25.4	123.0	35.4	33.2	22.4	0.29	0.94	-	-	-	-	-	3.0	-
Hathliacynidae	*Cladosictis patagonica*	MPM-PV 4326	-	-	-	-	21.8	-	-	-	-	-	-	14.4	10.7	-
Hathliacynidae	*Sipalocyon externus*	MACN-CH 1911	16.4	82.1	17.6	27.9	16.7	0.21	1.58	-	-	-	-	4.4	8.1	-
Hathliacynidae	*Sipalocyon gracilis*	AMNH-VP 9254	19.0	100.9	27.7	26.2	15.4	0.27	0.95	-	-	-	-	6.0	0.1	1.6
Hathliacynidae	*Sipalocyon* sp.	MPM-PV 4316	-	-	-	-	-	-	-	-	-	-	-	-	-	-
Felidae	*Smilodon populator*	MACN-PV 18057	88.3	290.2	70.5	102.5	73.9	0.24	1.45	-	-	-	-	2.5	0.6	1.3
Felidae	*Smilodon fatalis*	AMNH FAM14349	51.7	-	-	57.6	43.2	-	-	-	-	-	-	-	-	1.7
Felidae	*Homotherium serum*	TMM 933-3444	92.1	-	-	114.1	85.3	-	-	-	-	-	-	1.5	-	-
Felidae	*Panthera leo*	MVZ 117849	76.3	275.5	54.5	109.8	97.8	0.21	1.56		0.11	0.56	0.80	2.0	3.6	0.4
Felidae	*Panthera leo*	PRICT 1434	71.5	275.2	54.8	109.8	99.3	0.20	2.00	39.0[1,2]	0.14	0.71	0.78	2.8	1.6	2.2
Felidae	*Panthera leo*	PRICT 1435	72.6	334.1	69.0	107.4	99.2	0.20	2.01		0.14	0.71	0.78	1.6	1.6	1.4
Diprotodontia	*Thylacoleo carnifex*	SAM P16730	64.3	195.2	52.3	72.7	58.0	0.27	1.40	19.4	0.10	0.37	0.67	6.6	6.0	6.8
Dasyuridae	*Dasyurus hallucatus*	TMM M-6921	12.9	54.0	18.0	19.5	13.9	0.33	1.09		0.21	0.62	1.60	1.0	4.6	0.0
Dasyuridae	*Dasyurus maculatus*	UMZC A6.10/3	18.3	78.9	23.0	27.4	19.7	0.29	1.19	11.2[1]	0.14	0.49	1.13	2.4	2.9	1.5
Dasyuridae	*Sarcophilus harrisii*	IMNH R-1368	48.2	127.2	40.5	52.6	41.3	0.32	1.30	14.5[1]	0.11	0.36	0.70	1.1	0.2	0.6
Thylacinidae	*Thylacinus cynocephalus*	NMB c.2526	50.2	200.8	177.0	59.8	49.1	0.88	0.34	-	-	-	-	1.9	2.5	2.9
Didelphidae	*Didelphis virginiana*	TMM M-2517	22.8	106.9	38.1	27.5	19.6	0.36	0.72	9.9	0.09	0.26	1.00	0.1	0.0	0.1
Didelphidae	*Chironectes minimus*	MACN-Ma 24300	13.6	68.1	24.4	18.7	15.8	0.36	0.77	9.9	0.14	0.40	1.25	3.0	2.1	1.7

Table S4. Orbitolabyrinth angles for the specimens studied. Abbreviations: **CH19**, Casares-Hidalgo et al. (2019); **H05**, Heesy (2005); **F&G09**, Finarelli and Goswami (2009); **L**, left; **P&A17**, Pilatti and Astúa (2017); **R**, right.

Family	Taxon	Collection number	Orbitolabyrinth angle [°]							
			H05		F&G09		P&A17		CH19	
			L	R	L	R	L	R	L	R
Thylacosmilidae	*Thylacosmilus atrox*	FMNH P-14531	87.8	89.4	80.3	87.4	/	/	84.5	90.0
Hathliacynidae	*Sipalocyon gracilis*	AMNH-VP 9254	36.0	-	26.6	-	38.8	-	40.1	-
Felidae	*Smilodon fatalis*	AMNH FAM14349	82.4	87.5	80.3	88.1	87.2	77.6	82.1	87.9
Dasyuridae	*Dasyurus hallucatus*	TMM M-6921	60.0	59.5	69.8	68.5	62.9	61.9	59.0	59.8
Thylacinidae	*Thylacinus cynocephalus*	NMB c.2526	73.5	70.0	84.8	87.4	76.6	77.8	77.4	78.5
Didelphidae	*Didelphis virginiana*	TMM M-2517	77.8	71.7	88.8	83.5	75.1	70.6	75.1	70.6

Supplementary Data 1 (separate file). Selected data on orbital orientation of Carnivora and Marsupialia from the literature. Values in purple are our measurements (see Supplementary Tables S3) taken according to the landmarks given by the authors of the corresponding column. Abbreviations: **CH19**, Casares-Hidalgo et al. (2019); **H05**, Heesy (2005); **F&G09**, Finarelli and Goswami (2009); **P&A17**, Pilatti and Astúa (2017).

Supplementary Data 2 (separate file). Landmark coordinates for the specimen studied.

References

1. Prevosti, F. J., Forasiepi, A. M. & Zimicz, N. The evolution of the Cenozoic terrestrial mammalian predator guild in South America: competition or replacement? *J Mammal Evol* **20**, 3–21 (2013).
2. Prevosti, F. J. & Forasiepi, A. M. *Evolution of South American mammalian predators during the Cenozoic: Paleobiogeographic and paleoenvironmental contingencies*. (Springer International Publishing, 2018).

3. Zimicz, N. Avoiding Competition: the Ecological History of Late Cenozoic Metatherian Carnivores in South America. *J Mammal Evol* **21**, 383–393 (2014).
4. Argot, C. Functional adaptations of the postcranial skeleton of two Miocene borhyaenoids (Mammalia, Metatheria), *Borhyaena* and *Prothylacinus*, from South America. *Palaeontology* **46**, 1213–1267 (2003).
5. Argot, C. Postcranial functional adaptations in the South American Miocene borhyaenoids (Mammalia, Metatheria): *Cladosictis, Pseudonotictis* and *Sipalocyon*. *Alcheringa* **27**, 303–356 (2003).
6. Argot, C. Functional-adaptive features and palaeobiologic implications of the postcranial skeleton of the late Miocene sabretooth borhyaenoid *Thylacosmilus atrox* (Metatheria). *Alcheringa* **28**, 229–266 (2004).
7. Argot, C. Evolution of South American mammalian predators (Borhyaenoidea): anatomical and palaeobiological implications. *Zool J Linn Soc* **140**, 487–521 (2004).
8. Argot, C. Functional-adaptive analysis of the postcranial skeleton of a Laventan borhyaenoid, *Lycopsis longirostris* (Marsupialia, Mammalia). *J Vertebr Paleontol* **24**, 689–708 (2004).
9. Argot, C. & Babot, J. Postcranial morphology, functional adaptations and palaeobiology of *Callistoe vincei*, a predaceous metatherian from the Eocene of Salta, north-western Argentina: morphology and palaeobiology of *Callistoe vincei*. *Palaeontology* **54**, 447–480 (2011).

10. Ercoli, M. D., Prevosti, F. J. & Álvarez, A. Form and function within a phylogenetic framework: locomotory habits of extant predators and some Miocene Sparassodonta (Metatheria). *Zool J Linn Soc* **165**, 224–251 (2012).
11. Marshall, L. G. Evolution of the Thylacosmilidae, extinct saber-tooth marsupials of South Amercia. *PaleoBios* **23**, 1–3 (1976).
12. Marshall, L. G. *Evolution of the Borhyaenidae, extinct South American predaceous marsupials*. vol. 117 (1978).
13. Marshall, L. G. Review of the Prothylacyninae, an extinct subfamily of South American ‘dog-like’ marsupials. *Fieldiana Geol* **3**, 1–50 (1979).
14. Marshall, L. G. Review of the Hathlyacyninae, an extinct subfamily of South American ‘dog-like’ marsupials. *Fieldiana Geol* **7**, 1–120 (1981).
15. Wroe, S., Argot, C. & Dickman, C. On the rarity of big fierce carnivores and primacy of isolation and area: tracking large mammalian carnivore diversity on two isolated continents. *Proc R Soc Lond B* **271**, 1203–1211 (2004).
16. Tarquini, S. D., Ladevèze, S. & Prevosti, F. J. The multicausal twilight of South American native mammalian predators (Metatheria, Sparassodonta). *Sci Rep* **12**, 1224 (2022).
17. Degrange, F. J., Tambussi, C. P., Moreno, K., Witmer, L. M. & Wroe, S. Mechanical Analysis of Feeding Behavior in the Extinct “Terror Bird” *Andalgalornis steulleti* (Gruiformes: Phorusrhacidae). *PLoS ONE* **5**, e11856 (2010).
18. Ameghino, F. Enumeración sistemática de las especies de mamíferos fósiles coleccionados por Carlos Ameghino en los terrenos eocenos de la

Patagonia Austral y depositados en el Museo de La Plata. *Bol Mus la Plata* **1**, 1–26 (1887).

19. Prevosti, F. J., Forasiepi, A. M., Ercoli, M. D. & Turazzini, G. F. Paleoecology of the mammalian carnivores (Metatheria, Sparassodonta) of the Santa Cruz Formation (late Early Miocene). in *Early Miocene Paleobiology in Patagonia* (eds. Vizcaino, S. F., Kay, R. F. & Bargo, M. S.) 173–193 (Cambridge University Press, 2012). doi:10.1017/CBO9780511667381.012.
20. Echarri, S., Ulloa-Guaiquin, K. S., Aguirrezabala, G. & Forasiepi, A. M. Cladosictis patagonica (Metatheria, Sparassodonta) from the Collón Cura Formation (Middle Miocene), Río Negro, Argentina. *Ameghiniana* **58**, 485–491 (2021).
21. Ercoli, M. D., Prevosti, F. J. & Forasiepi, A. M. The Structure of the Mammalian Predator Guild in the Santa Cruz Formation (Late Early Miocene). *J Mammal Evol* **21**, 369–381 (2013).
22. Marshall, L. G. A New Species of Lycopsis (Borhyaenidae: Marsupialia) from the La Venta Fauna (Late Miocene) of Colombia, South America. *J Paleontol* **51**, 633–642 (1977).
23. Forasiepi, A. M., Francisco, J. G. & Tauber, A. A. Las especies de *Arctodictis* Mercerat, 1891 (Metatheria, Borhyaenidae), grandes carnívoros del mioceno de América del Sur. *Rev Esp Paleontol* **19**, 1–22 (2004).

24. de Muizon, C. Marsupial skulls from the Deseadan (late Oligocene) of Bolivia and phylogenetic analysis of the Borhyaenoidea (Marsupialia, Mammalia). *Geobios* **32**, 483–509 (1999).
25. Engelman, R. K., Anaya, F. & Croft, D. A. New Specimens of Acyon myctoderos (Metatheria, Sparassodonta) from Quebrada Honda, Bolivia. *Ameghiniana* **52**, 204–225 (2015).
26. Ameghino, F. Enumération synoptique des espèces de mammifères fossiles des formations éocènes de Patagonie. *Bol Acad Nac Cienc* **13**, 259–452 (1894).
27. Sinclair, W. J. Mammalia of the Santa Cruz beds. Marsupialia. in *Reports of the Princeton University Expeditions to Patagonia, 1896-1899* (ed. Scott, W. B.) vol. 4 333–460 (Schweizerbart'sche Verlagshandlung, 1906).
28. Wood, H. E. The position of the "sparassodonts": with notes on the relationships and history of the Marsupialia. *Bull Am Mus Nat Hist* **51**, 77–101 (1924).
29. Simpson, G. G. The development of marsupials in South America. *Physis* **14**, 373–398 (1939).
30. Simpson, G. G. The principles of classification and a classification of mammals. *Bull Am Mus Nat Hist* **85**, 1–350 (1945).
31. Matthew, W. D. The Relationships of the 'Sparassodonta.' *Geol Mag* **4**, 531–535 (1907).
32. Gregory, W. K. The orders of mammals. *Bull Am Mus Nat Hist* **27**, 1–524 (1910).

33. Simpson, G. G. The affinities of Borhyaenidae. *Am Mus Novit* **1130**, 1–12 (1941).

34. Archer, M. The basicranial region of marsupicarnivores (Marsupialia), interrelationships of carnivorous marsupials, and affinities of the insectivorous marsupial peramelids. *Zool J Linn Soc* **59**, 217–322 (1976).

35. de Muizon, C., Cifelli, R. L. & Paz, R. C. The origin of the dog-like borhyaenoid marsupials of South America. *Nature* **389**, 486–489 (1997).

36. de Muizon, C. *Mayulestes ferox*, a borhyanoid (Metatheria, Mammalia) from the early Palaeocene of Bolivia. Phylogenetic and palaeobiologic implications. *Geodiversitas* **20**, 19–142 (1998).

37. Szalay, F. S. Phylogenetic relationships of the marsupials. *Geobios* **15**, 177–190 (1982).

38. Marshall, L. G. & Kielan-Jaworowska, Z. Relationships of the dog-like marsupials, deltatheroidans and early tribosphenic mammals. *Lethaia* **25**, 361–374 (1992).

39. Rougier, G. W., Wible, J. R. & Novacek, M. J. Implications of *Deltatheridium* specimens for early marsupial history. *Nature* **396**, 459–463 (1998).

40. Babot, M. J. Los Borhyaenoidea (Mammalia, Metatheria) del Terciario Inferior del Noroeste Argentino. Aspectos filogenéticos, paleobiológicos y bioestratigráficos. (Universidad Nacional de Tucumán, 2005).

41. Forasiepi, A. M. Osteology of *Arctodictis sinclairi* (Mammalia, Metatheria, Sparassodonta) and phylogeny of Cenozoic metatherian carnivores from

South America. *Monografías Mus Arg Sci Nat Bernardino Rivadavia [ns]* **6**, 1–174 (2009).

42. Engelman, R. K. & Croft, D. A. A new species of small-bodied sparassodont (Mammalia, Metatheria) from the middle Miocene locality of Quebrada Honda, Bolivia. *J Vertebr Paleontol* **34**, 672–688 (2014).
43. Forasiepi, A. M., Judith Babot, M. & Zimicz, N. *Australohyaena antiqua* (Mammalia, Metatheria, Sparassodonta), a large predator from the Late Oligocene of Patagonia. *J Syst Palaeontol* **13**, 503–525 (2015).
44. Suarez, C., Forasiepi, A. M., Goin, F. J. & Jaramillo, C. Insights into the Neotropics prior to the Great American Biotic Interchange: new evidence of mammalian predators from the Miocene of Northern Colombia. *J Vertebr Paleontol* **36**, e1029581 (2016).
45. de Muizon, C., Ladevèze, S., Selva, C., Vignaud, R. & Goussard, F. *Allqokirus australis* (Sparassodonta, Metatheria) from the early Paleocene of Tiumpampa (Bolivia) and the rise of the metatherian carnivorous radiation in South America. *Geodiversitas* **40**, 363–459 (2018).
46. de Muizon, C. & Ladevèze, S. Cranial anatomy of *Andinodelphys cochabambensis*, a stem metatherian from the early Palaeocene of Bolivia. *Geodiversitas* **42**, 597–739 (2020).
47. Simpson, G. G. The beginning of the age of mammals in South America. *Bull Am Mus Nat Hist* **91**, 1–232 (1948).

48. Couto, C. de P. Fossil mammals from the beginning of the Cenozoic in Brazil. Marsupialia: Polydolopidae and Borhyaenidae. *Am Mus Novit* **1559**, 1–27 (1959).
49. Goin, F. J., Palma, R. M., Pascual, R. & Powell, J. E. Persistencia de un primitivo Borhyaenidae (Mammalia, Marsupialia) en el Eoceno temprano de Salta (Fm. Lumbrera, Argentina). Aspectos geologicos y paleoambientales relacionados. *Ameghiniana* **23**, 47–56 (1986).
50. Goin, F. J. & Candela, A. New Paleogene marsupials from the Amazon Basin of Eastern Peru. in *The Paleogene mammalian fauna of Santa Rosa, Amazonian Peru* (ed. Campbell) 15–60 (2004).
51. Forasiepi, A. M., MacPhee, R. D. E. & Pino, S. H. del. Caudal Cranium of *Thylacosmilus atrox* (Mammalia, Metatheria, Sparassodonta), a South American Predaceous Sabertooth. *Bull Am Mus Nat Hist* **433**, 1–64 (2019).
52. de Muizon, C. A new carnivorous marsupial from the Palaeocene of Bolivia and the problem of marsupial monophyly. *Nature* **370**, 208–211 (1994).
53. Goin, F. J. Early marsupial radiation in South America. in *Predators with pouches: The biology of carnivorous marsupials* (eds. Jones, M., Dickman, C. & Archer, M.) 30–42 (2003).
54. Wilson, G. P., Ekdale, E. G., Hoganson, J. W., Calede, J. J. & Vander Linden, A. A large carnivorous mammal from the Late Cretaceous and the North American origin of marsupials. *Nat Commun* **7**, 13734 (2016).
55. Engelman, R. K., Flynn, J. J., Wyss, A. R. & Croft, D. A. *Eomakhaira molossus*, a new saber-toothed sparassodont (Metatheria: Thylacosmilinae)

from the Early Oligocene (?Tinguiririrican) Cachapoal Locality, Andean Main Range, Chile. *Am Mus Novit* 1–79 (2020) doi:10.1206/3957.1.

56. Carneiro, L. M. A new species of *Varalphadon* (Mammalia, Metatheria, Sparassodonta) from the upper Cenomanian of southern Utah, North America: Phylogenetic and biogeographic insights. *Cretac Res* **84**, 88–96 (2018).
57. Johanson, Z. Revision of the Late Cretaceous North American marsupial genus *Alphadon*. *Palaeontogr Abt A* **242**, 127–184 (1996).
58. Williamson, T. E., Brusatte, S. L. & Wilson, G. P. The origin and early evolution of metatherian mammals: the Cretaceous record. *ZK* **465**, 1–76 (2014).
59. Rowe, T. Definition and Diagnosis in the Phylogenetic System. *Syst Zool* **36**, 208–211 (1987).
60. Rowe, T. Definition, diagnosis, and origin of Mammalia. *J Vertebr Paleontol* **8**, 241–264 (1988).
61. Rowe, T. & Gauthier, J. Ancestry, paleontology, and definition of the name Mammalia. *Syst Biol* **41**, 372–378 (1992).
62. Simpson, G. G. History of the fauna of latin America. *American Scientist* **38**, 361–389 (1950).
63. Simpson, G. G. *Splendid isolation: the curious history of South American mammals*. (Yale University Press, 1980).
64. Babot, J. & Forasiepi, A. M. Mamíferos predadores nativos del Cenozoico sudamericano: evidencias filogenetics y paleoecológicas. in *Contribuciones*

Científicas del Museo Argentino de Ciencias Naturales 'Bernardino Rivadavia' vol. 6 219–230 (2016).

65. Goin, F. J. & Pascual, R. News on the biology and taxonomy of the marsupials Thylacosmilidae (Late Tertiary of Argentina). *Anales Acad Cienc Exactas Fis Nat* **39**, 219–246 (1987).
66. Prevosti, F. J., Turazzini, G. F. & Amelia Chemisquy, M. Morfología craneana en tigres dientes de sable: alometría, función y filogenia. *Ameghiniana* **47**, 239–256 (2010).
67. Riggs, E. S. Preliminary description of a new marsupial sabertooth from the Pliocene of Argentina. *Field Mus Nat Hist Geol Ser* **6**, 61–66 (1933).
68. Riggs, E. S. A new marsupial saber-tooth from the Pliocene of Argentina and its relationships to other South American predacious marsupials. *Trans Am Philos Soc [ns]* **24**, 1–32 (1934).
69. Simpson, G. G. The evolution of marsupials in South America. *An Acad Bras Cienc* **43**, 103–118 (1971).
70. Turnbull, W. D. Another look at dental specialization in the extinct saber-toothed marsupial, *Thylacosmilus*, compared with its placental counterparts. in *Development, function and evolution of teeth* (eds. Butler, P. M. & Joysey, K. A.) 399–414 (1978).
71. Turnbull, W. D. & Segall, W. The ear region of the marsupial sabertooth, *Thylacosmilus*: Influence of the sabertooth lifestyle upon it, and convergence with placental sabertooths. *J Morphol* **181**, 239–270 (1984).

72. Churcher, C. S. Dental functional morphology in the marsupial sabre-tooth *Thylacosmilus atrox* (Thylacosmilidae) compared to that of felid saber-tooths. *Aust Mammal* **8**, 201–220 (1985).

73. Janis, C. M., Figueirido, B., DeSantis, L. & Lautenschlager, S. An eye for a tooth: Thylacosmilus was not a marsupial 'saber-tooth predator'. *PeerJ* **8**, e9346 (2020).

74. Goin, F. J. New clues for understanding Neogene marsupials radiations. in *A history of the Neotropical fauna. Veterbrate paleobiology of the Miocene of Colombia* (eds. Kay, R. F., Madden, R. H., Cifelli, R. L. & Flynn, J. J.) 185–204 (1997).

75. Mones, A. & Rinderknecht, A. Primer registro de Thylacosmilidae en el Uruguay (Mammalia: Marsupiali: Sparassodonta). *Comunicaciones Paleontologicas Mus Nac Hist Nat Antropol* **2**, 193–200 (2004).

76. Forasiepi, A. M. & Carlini, A. A. A new thylacosmilid (Mammalia, Metatheria, Sparassodonta) from the Miocene of Patagonia, Argentina. *Zootaxa* **2552**, 55–68 (2010).

77. van Valkenburgh, B. & Koepfli, K.-P. Cranial and dental adaptations to predation in canids. *Symp Zool Soc Lond* 15–37 (1993).

78. van Valkenburgh, B. Iterative evolution of hypercarnivory in canids (Mammalia: Carnivora): evolutionary interactions among sympatric predators. *Paleobiology* **17**, 340–362 (1991).

79. Echarri, S., Ercoli, M. D., Chemisquy, M. A., Turazzini, G. & Prevosti, F. J. Mandible morphology and diet of the South American extinct metatherian

predators (Mammalia, Metatheria, Sparassodonta). *Earth Environ Sci Trans R Soc Edinb* **106**, 277–288 (2017).

80. Wroe, S. *et al.* Comparative Biomechanical Modeling of Metatherian and Placental Saber-Tooths: A Different Kind of Bite for an Extreme Pouched Predator. *PLoS ONE* **8**, e66888 (2013).

81. Wroe, S., McHenry, C. & Thomason, J. Bite club: comparative bite force in big biting mammals and the prediction of predatory behaviour in fossil taxa. *Proc R Soc B* **272**, 619–625 (2005).

82. Blanco, R. E., Jones, W. W. & Grinspan, G. A. Fossil marsupial predators of South America (Marsupialia, Borhyaenoidea): bite mechanics and palaeobiological implications. *Alcheringa* **35**, 377–387 (2011).

83. Sorkin, B. A biomechanical constraint on body mass in terrestrial mammalian predators. *Lethaia* **41**, 333–347 (2008).

84. Wroe, S. *et al.* An alternative method for predicting body mass: the case of the Pleistocene marsupial lion. *Paleobiology* **29**, 403–411 (2003).

85. Ercoli, M. D. & Prevosti, F. J. Estimación de Masa de las Especies de Sparassodonta (Mammalia, Metatheria) de Edad Santacrucense (Mioceno Temprano) a Partir del Tamaño del Centroide de los Elementos Apendiculares: Inferencias Paleoecológicas. *Ameghiniana* **48**, 462–479 (2011).

86. Forasiepi, A. M., Martinelli, A. G. & Goin, F. J. Revisión taxonómica de *Parahyaenodon argentinus* Ameghino y sus implicancias en el conocimiento

de los grandes mamíferos carnívoros del Mio-Plioceno de América de Sur. *Ameghiniana* **44**, 143–159 (2007).

87. Álvarez, D. & Tauber, A. Vertebrados de la Formación Brochero (Mioceno Tardío-Plioceno) de Córdoba, Argentina. in *Ameghiniana* vol. 41 (4) 32R-33R (2004).

88. Goin, F. J., Montalvo, C. I. & Visconti, G. Los marsupiales (Mammalia) del Mioceno superior de la Formación Cerro Azul (Provincia de La Pampa, Argentina). *Estud geol* **56**, 101–126 (2000).

89. Cione, A. L. *et al.* Miocene vertebrates from Entre Ríos province, eastern Argentina. in *El Neógeno de Argentina* (eds. Aceñolaza, F. G. & Herbst, R.) 191–237 (2000).

90. Kraglievich, J. L. Marsupiales tilacosmilinos de la fauna de Chapadmalal. *Rev Mus Munic Cienc Nat Tradic Mar del Plata* **1**, 53–72 (1960).

91. Riggs, E. S. New family of South American Pliocene mammals. *Bull Geol Soc Am* **40**, 117 (1929).

92. Suárez Gómez, S. C. Estudios taxonómicos y paleobiológicos sobre los Metatheria (Mammalia) del Mioceno medio de La Venta, Colombia. (Universidad Nacional de La Plata, 2019).

93. Goin, F. J., Abello, A., Bellosi, E., Kay, R. & Madden, R. Los Metatheria sudamericanos de comienzos del Neógeno (Mioceno Temprano, Edad-mamífero Colhuehuapense). Parte I: Introducción, Didelphimorphia y Sparassodonta. *Ameghiniana* **44**, 29–71 (2007).

94. Babot, M. J., Powell, J. E. & de Muizon, C. *Callistoe vincei*, a new Proborhyaenidae (Borhyaenoidea, Metatheria, Mammalia) from the Early Eocene of Argentina. *Geobios* **35**, 615–629 (2002).
95. Ameghino, F. Première contribution à la connaissance de la faune mammalogique des couches à *Colpodon*. *Bol Acad Cienc Córdoba* **17**, 71–141 (1902).
96. Ameghino, F. Mammifères crétacés de l'Argentine. Deuxième contribution à la connaissance de la faune mammalogique des conches à *Pyrotherium*. *Bol Inst Geogr Arg* **18**, 406–521 (1897).
97. Ameghino, F. Notice préliminaire sur les mammifères nouveaux des terrains Crétacés de Patagonie. *Bol Acad Cienc Córdoba* **17**, 5–70 (1902).
98. Kerr, R. *The animal kingdom or zoological system of the celebrated Sir Charles Linnaeus. Class I. Mammalia*. (W. Creech, 1792).
99. Zimmermann, E. A. W. *Geographische Geschichte des Menschen, und der vierfüssigen Thiere*. vol. 2 (Weygandschen Buchhandlung, 1780).
100. Gould, J. Characters of a new species of *Perameles*, and a new species of *Dasyurus*. *Proc Zool Soc Lond* 41–42 (1842).
101. Boitard, M. *Le jardin des plantes: description et moeurs des mammifères de la ménagerie et du musem d'histoire naturelle*. (Gustave Barba, 1841).
102. Harris, G. P. Description of two new species of *Didelphis* from Van Diemens Land. *Trans Linn Soc Lond* **9**, 4–8 (1808).
103. Owen, R. On the fossil mammals of Australia.— Part I. Description of a mutilated skull of a large marsupial carnivore (*Thylacoleo carnifex*, Owen),

from a calcareous conglomerate stratum, eighty miles S. W. of Melbourne, Victoria. *Phil Trans R Soc B* **149**, 309–322 (1859).

104. Linnaeus, C. *Systema Naturae per regna tria naturae, secundum classes, ordines, genera, species, cum characteribus, differentiis, synonymis, locis*. vol. I (Laurentius Salvius: Holmiae, 1758).

105. Lund, P. W. Blik paa Brasiliens Dyreverden för sidste Jordomvaeltning. Fierde Afhandling: Forstsaettelse af Pattedyrene. *Det Kongelige Danske Videnskabernes Selskab Naturvidenskabelige og Mathermatiske Afhandlinger* **9**, 137–208 (1842).

106. Cope, E. D. A new Plistocene sabre-tooth. *Am Nat* **27**, 896–897 (1893).

107. Leidy, J. The extinct mammalian fauna of Dakota and Nebraska, including an account of some allied forms from other localities, together with a synopsis of the mammalian remains of North America. *J Acad Nat Sci Philadelphia* **7**, 1–473 (1869).

108. Macrini, T. E. *Didelphis virginiana*. (On-line), Digital Morphology. Accessed at http://digimorph.org/specimens/Didelphis_virginiana/ http://digimorph.org/specimens/Didelphis_virginiana/ (2005).

109. Macrini, T. E. *Dasyurus hallucatus*. (On-line), Digital Morphology. Accessed at http://digimorph.org/specimens/Dasyurus_hallucatus/ http://digimorph.org/specimens/Dasyurus_hallucatus/ (2005).

110. van Valkenburgh, B. *Homotherium serum*. (On-line), Digital Morphology. Accessed at http://digimorph.org/specimens/Homotherium_serum/ http://digimorph.org/specimens/Homotherium_serum/ (2007).

111. Owen, P. *Panthera leo*. (On-line), Digital Morphology. Accessed at http://digimorph.org/specimens/Panthera_leo/adult/ http://digimorph.org/specimens/Panthera_leo/adult/ (2002).
112. Martín-Serra, A. & Benson, R. B. J. Developmental constraints do not influence long-term phenotypic evolution of marsupial forelimbs as revealed by interspecific disparity and integration patterns. *Am Nat* **195**, 547–560 (2020).
113. Tseng, Z. J., Grohé, C. & Flynn, J. J. A unique feeding strategy of the extinct marine mammal *Kolponomos* : convergence on sabretooths and sea otters. *Proc R Soc B* **283**, 20160044 (2016).
114. Wroe, S. Cranial mechanics compared in extinct marsupial and extant African lions using a finite-element approach. *J Zool* **274**, 332–339 (2008).
115. Fedorov, A. *et al.* 3D Slicer as an image computing platform for the Quantitative Imaging Network. *Magn Reson Imaging* **30**, 1323–1341 (2012).
116. Heesy, C. P. The evolution of orbit orientation in mammals and the function of the primate postorbital bar. (Stony Brook University, 2003).
117. Heesy, C. P. Function of the mammalian postorbital bar. *J Morphol* **264**, 363–380 (2005).
118. Finarelli, J. A. & Goswami, A. The evolution of orbit orientation and encephalization in the Carnivora (Mammalia). *J Anat* **214**, 671–678 (2009).
119. Pilatti, P. & Astúa, D. Orbit orientation in didelphid marsupials (Didelphimorphia: Didelphidae). *Curr Zool* **63**, 403–415 (2017).

120. Casares-Hidalgo, C., Pérez-Ramos, A., Forner-Gumbau, M., Pastor, J. F. & Figueirido, B. Taking a look into the orbit of mammalian carnivorans. *J. Anat.* **234**, 622–636 (2019).
121. Heesy, C. P. On the relationship between orbit orientation and binocular visual field overlap in mammals. *Anat Rec* **281A**, 1104–1110 (2004).
122. Cox, P. G. A quantitative analysis of the Eutherian orbit: correlations with masticatory apparatus. *Biol Rev* **83**, 35–69 (2008).
123. Rosenberger, A. L. *et al.* Eye size and set in small-bodied fossil primates: A three-dimensional method: Determining eye size and set in fossil primates. *Anat Rec* **299**, 1671–1689 (2016).
124. Ross, C. F. & Kirk, E. C. Evolution of eye size and shape in primates. *J Hum Evol* **52**, 294–313 (2007).
125. Howland, H. C., Merola, S. & Basarab, J. R. The allometry and scaling of the size of vertebrate eyes. *Vis Res* **44**, 2043–2065 (2004).
126. Kirk, E. C. Visual influences on primate encephalization. *J Hum Evol* **51**, 76–90 (2006).
127. Wible, J. R. On the cranial osteology of the short-tailed opossum Monodelphis brevicaudata (Didelphidae, Marsupialia). *Ann Carnegie Mus* **72**, 137–202 (2003).
128. Kuhn, H.-J. & Zeller, U. The cavum epiptericum in monotremes and therian mammals. in *Morphogenesis of the Mammalian Skull* (eds. kuhn, H.-J. & Zeller, U.) 51–70 (V. P. Parey, 1987).

129. Cartmill, M. The orbits of arboreal mammals: a reassessment of the arboreal theory of primate evolution. (University of Chicago, 1970).
130. Heesy, C. P. Ecomorphology of Orbit Orientation and the Adaptive Significance of Binocular Vision in Primates and Other Mammals. *Brain Behav Evol* **71**, 54–67 (2008).
131. Jeffery, N. & Cox, P. G. Do agility and skull architecture influence the geometry of the mammalian vestibulo-ocular reflex? *J Anat* **216**, 496–509 (2010).
132. Simpson, J. I. & Graf, W. Eye-muscle geometry and compensatory eye movements in lateral-eyed and frontal-eyed animals. *Ann N Y Acad Sci* **374**, 20–30 (1981).
133. Graf, W. & Brunken, W. J. Elasmobranch oculomotor organization: anatomical and theoretical aspects of the phylogenetic development of vestibulo-oculomotor connectivity. *J Comp Neurol* **227**, 569–581 (1984).
134. Noble, V. E., Kowalski, E. M. & Ravosa, M. J. Orbit orientation and the function of the mammalian postorbital bar. *J Zool* **250**, 405–418 (2000).
135. Bookstein, F. L. *Morphometric tools for landmark data geometric and biology*. (Cambridge University Press, 1992).
136. Zelditch, M. L., Swiderski, D. L., Sheets, H. D. & Fink. *Geometric morphometrics for biologists: a primer*. (Elsevier Academic Press, 2004).
137. Shearer, B. M. *et al.* Evaluating causes of error in landmark-based data collection using scanners. *PLoS ONE* **12**, e0187452 (2017).

138. Sánchez-Villagra, M. R. & Asher, R. J. Cranio-sensory adaptations in small faunivorous semiaquatic mammals, with special reference to olfaction and the trigeminal system. *Mammalia* **66**, 93–110 (2002).
