# Supplementary material for: Seeing through the eyes of the sabertooth Thylacosmilus atrox (Metatheria, Sparassodonta)
Source: Commun Biol. 2023 Mar 21;6:257. doi: 10.1038/s42003-023-04624-5 (PMC10030895; doi:10.1038/s42003-023-04624-5)
Supplement: Supplementary file 2 — Description of Additional Supplementary Files [file 42003_2023_4624_MOESM2_ESM.pdf]

Description of Additional Supplementary Files

File name: Supplementary Data 1
Description: Selected data on orbital orientation of Carnivora and Marsupialia from the literature. Values in purple are our measurements (see Supplementary Tables S3) taken according to the landmarks given by the authors of the corresponding column. Abbreviations: CH19, Casares-Hidalgo et al. (2019); H05, Heesy (2005); F&G09, Finarelli and Goswami (2009); P&A17, Pilatti and Astúa (2017).

File name: Supplementary Data 2
Description: Landmark coordinates.
