# Supplementary material for: Seeing through the eyes of the sabertooth Thylacosmilus atrox (Metatheria, Sparassodonta)
Source: Commun Biol. 2023 Mar 21;6:257. doi: 10.1038/s42003-023-04624-5 (PMC10030895; doi:10.1038/s42003-023-04624-5)
Supplement: Supplementary file 5 — Reporting Summary [file 42003_2023_4624_MOESM5_ESM.pdf]

nature portfolio

Corresponding author(s): Charlène Gaillard

Last updated by author(s): Feb 7, 2023

Reporting Summary

Nature Portfolio wishes to improve the reproducibility of the work that we publish. This form provides structure for consistency and transparency in reporting. For further information on Nature Portfolio policies, see our Editorial Policies and the Editorial Policy Checklist.

Statistics

For all statistical analyses, confirm that the following items are present in the figure legend, table legend, main text, or Methods section.

n/a	Confirmed	
☐	☒	The exact sample size (n) for each experimental group/condition, given as a discrete number and unit of measurement
☐	☒	A statement on whether measurements were taken from distinct samples or whether the same sample was measured repeatedly
☐	☒	The statistical test(s) used AND whether they are one- or two-sided<br>*Only common tests should be described solely by name; describe more complex techniques in the Methods section.*
☐	☒	A description of all covariates tested
☐	☒	A description of any assumptions or corrections, such as tests of normality and adjustment for multiple comparisons
☐	☒	A full description of the statistical parameters including central tendency (e.g. means) or other basic estimates (e.g. regression coefficient) AND variation (e.g. standard deviation) or associated estimates of uncertainty (e.g. confidence intervals)
☒	☐	For null hypothesis testing, the test statistic (e.g. F, t, r) with confidence intervals, effect sizes, degrees of freedom and P value noted<br>*Give P values as exact values whenever suitable.*
☒	☐	For Bayesian analysis, information on the choice of priors and Markov chain Monte Carlo settings
☒	☐	For hierarchical and complex designs, identification of the appropriate level for tests and full reporting of outcomes
☒	☐	Estimates of effect sizes (e.g. Cohen's d, Pearson's r), indicating how they were calculated

Our web collection on statistics for biologists contains articles on many of the points above.

Software and code

Policy information about availability of computer code

Data collection	3D Slicer 4.11.20200930
Data analysis	3D Slicer 4.11.20200930, 3-matic Research 13.0, Microsoft Excel 2016

For manuscripts utilizing custom algorithms or software that are central to the research but not yet described in published literature, software must be made available to editors and reviewers. We strongly encourage code deposition in a community repository (e.g. GitHub). See the Nature Portfolio guidelines for submitting code & software for further information.

Data

Policy information about availability of data

All manuscripts must include a data availability statement. This statement should provide the following information, where applicable:

- Accession codes, unique identifiers, or web links for publicly available datasets
- A description of any restrictions on data availability
- For clinical datasets or third party data, please ensure that the statement adheres to our policy

All data are given in the Dataset and Supplementary Data files attached to the manuscript

Human research participants

Policy information about studies involving human research participants and Sex and Gender in Research.

Reporting on sex and gender	This information has not been collected
Population characteristics	See above
Recruitment	No participants were recruited
Ethics oversight	No ethical protocol was required

Note that full information on the approval of the study protocol must also be provided in the manuscript.

Field-specific reporting

Please select the one below that is the best fit for your research. If you are not sure, read the appropriate sections before making your selection.

☐ Life sciences ☐ Behavioural & social sciences ☒ Ecological, evolutionary & environmental sciences

For a reference copy of the document with all sections, see nature.com/documents/nr-reporting-summary-flat.pdf

Ecological, evolutionary & environmental sciences study design

All studies must disclose on these points even when the disclosure is negative.

Study description	The study quantify the orbital orientation of the extinct metatherians Thylacosmilus atrox (Sparassodonta). Orbit orientation is quantified through equations using landmarks coordinates. Landmarks were taken on 3D digital models of specimens that were scanned for the study (information provided in Supplementary Data file). Angles of orbital orientation are compared between the specimens studied and data available in the litterature.
Research sample	The study involves 16 specimens of the non-marsupial metatherians Sparassodonta and 13 specimens of extant and extinct marsupials and carnivores.
Sampling strategy	Sample size was chosen based on the availability of fossil specimens and their preservation
Data collection	Co-authors collected the raw data (CT) and corresponding author collected the resulting data (landmarks). Data from other authors is given in the Dataset file.
Timing and spatial scale	Time scale is from early Paleocene to early Pliocene.
Data exclusions	Specimens that were too much deformed were not included in the study
Reproducibility	All attempt to repeat the study were successful
Randomization	This is not relevant to the study
Blinding	This is not relevant to the study

Did the study involve field work? ☐ Yes ☒ No

Reporting for specific materials, systems and methods

We require information from authors about some types of materials, experimental systems and methods used in many studies. Here, indicate whether each material, system or method listed is relevant to your study. If you are not sure if a list item applies to your research, read the appropriate section before selecting a response.

Materials & experimental systems

n/a	Involved in the study	
☒	☐	Antibodies
☒	☐	Eukaryotic cell lines
☐	☒	Palaeontology and archaeology
☐	☒	Animals and other organisms
☒	☐	Clinical data
☒	☐	Dual use research of concern

Methods

n/a	Involved in the study	
☒	☐	ChIP-seq
☒	☐	Flow cytometry
☒	☐	MRI-based neuroimaging

Palaeontology and Archaeology

Specimen provenance	Specimen are mainly from Argentina and are deposited in Museums (see below).
Specimen deposition	Specimens are deposited in the following institutions: AMNH (New York), FMNH (Chicago), IMNH, Poncatello, TMM (Austin), MVZ (Berkley), MACN (Buenos Aires), MCNAM (Mendoza), MLP (La Plata), MMP (Mar del Plata), MPM (Santa Cruz), NMB (Basel), UMYC (Cambridge), SAM (Adelaide), and PRICT (Inuyama). Full list of museums and their information is gien in the Supplementary Information attached to the manuscript.
Dating methods	No new dates are provided

☒ Tick this box to confirm that the raw and calibrated dates are available in the paper or in Supplementary Information.

Ethics oversight	No ethical protocol was required

Note that full information on the approval of the study protocol must also be provided in the manuscript.

Animals and other research organisms

Policy information about studies involving animals; ARRIVE guidelines recommended for reporting animal research, and Sex and Gender in Research

Laboratory animals	The study did not involve laboratory animals
Wild animals	The study involve skulls of wild extant animals deposited in the Museum described in the section "Palaeontology and Archaelogy".
Reporting on sex	This information has not been collected
Field-collected samples	The study did not involve new specimens collected in the field
Ethics oversight	No ethical protocol was required

Note that full information on the approval of the study protocol must also be provided in the manuscript.
